# Supplementary figures and images for: CDK-mediated phosphorylation of PNKP is required for end-processing of single-strand DNA gaps on Okazaki fragments and genome stability
Source: eLife. 2025 Mar 27;14:e99217. doi: 10.7554/eLife.99217 (PMC11949490; doi:10.7554/eLife.99217)

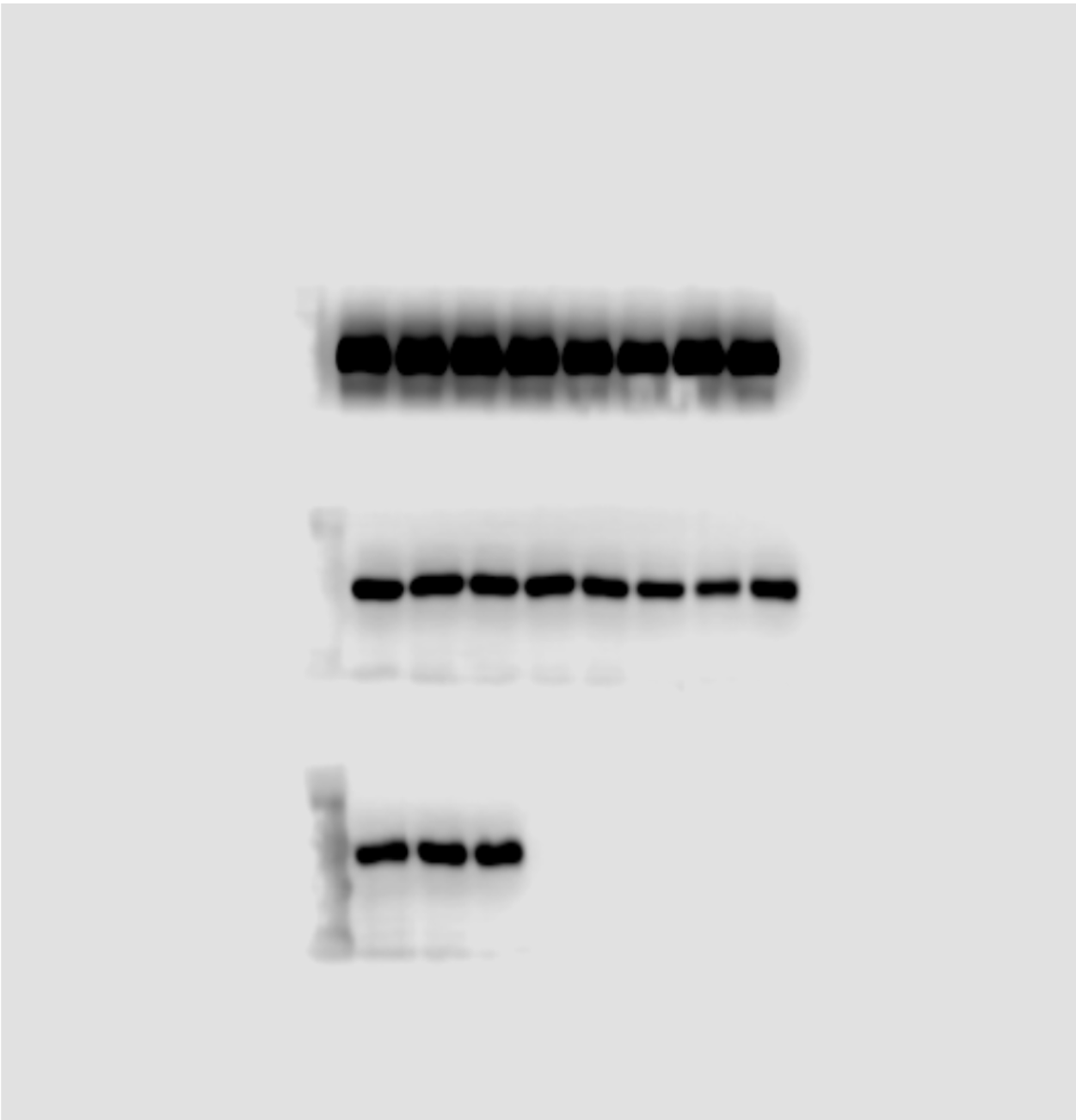

Supplement: Figure 1—source data 2. [file elife-99217-fig1-data2.zip › Figure 1, Source Data1/PCNA.tif]

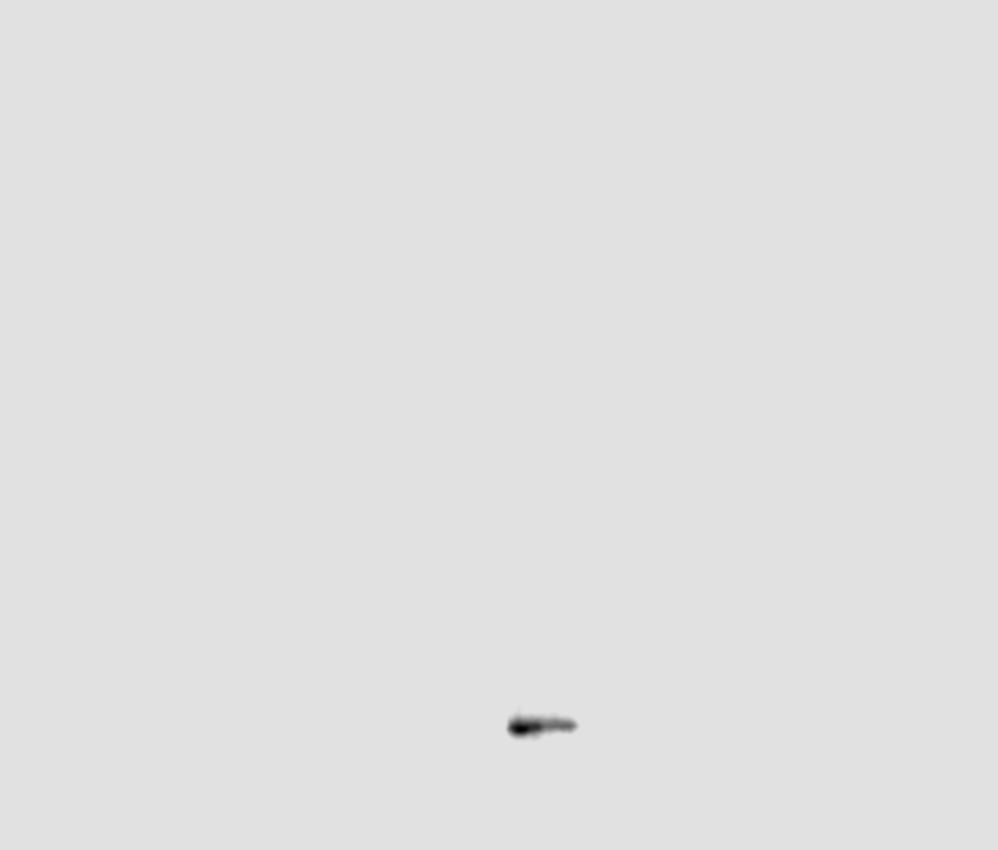

Supplement: Figure 1—source data 2. [file elife-99217-fig1-data2.zip › Figure 1, Source Data1/PNKP (C-terminal).tif]

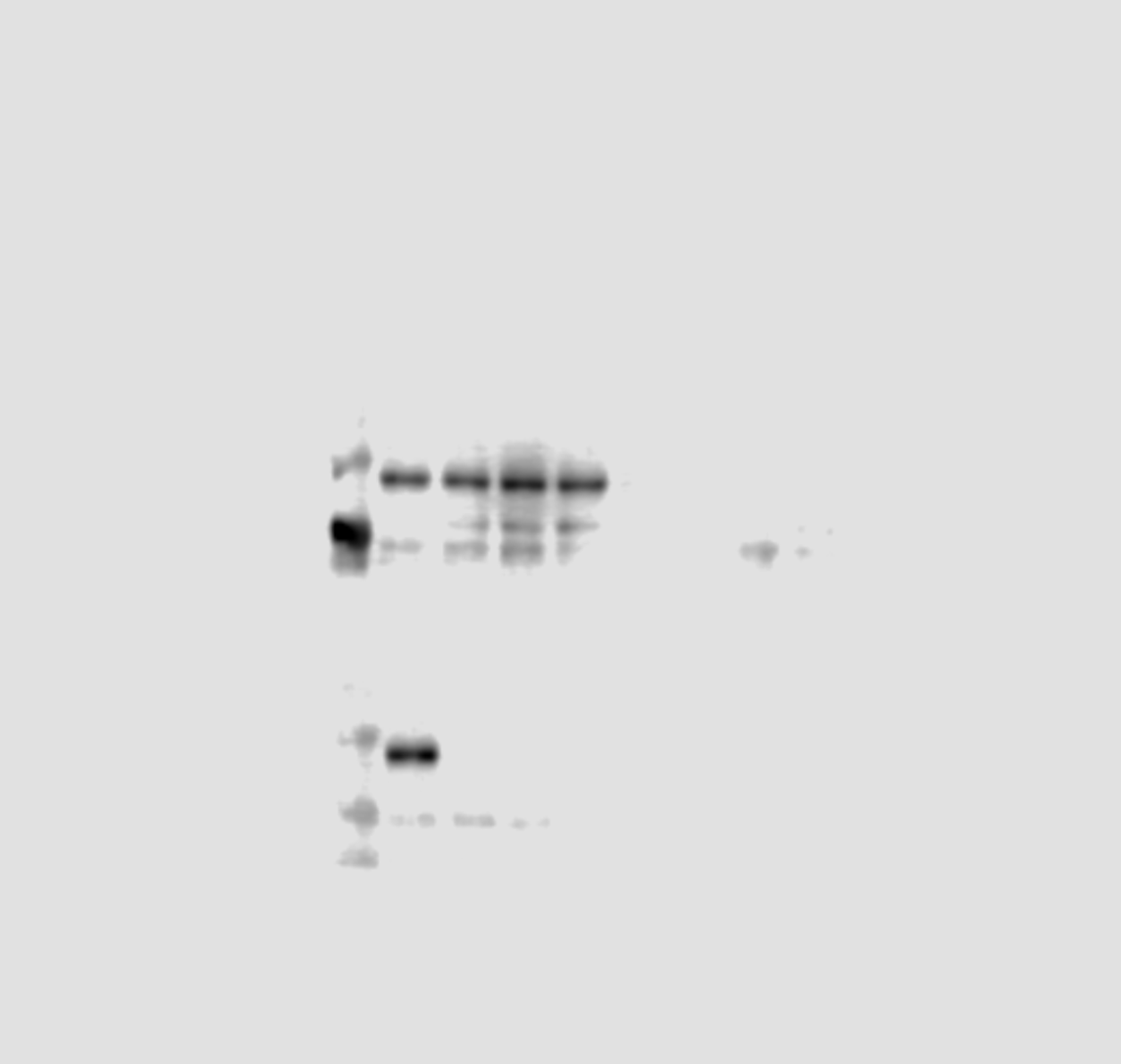

Supplement: Figure 1—source data 2. [file elife-99217-fig1-data2.zip › Figure 1, Source Data1/PNKP (N-terminal).tif]

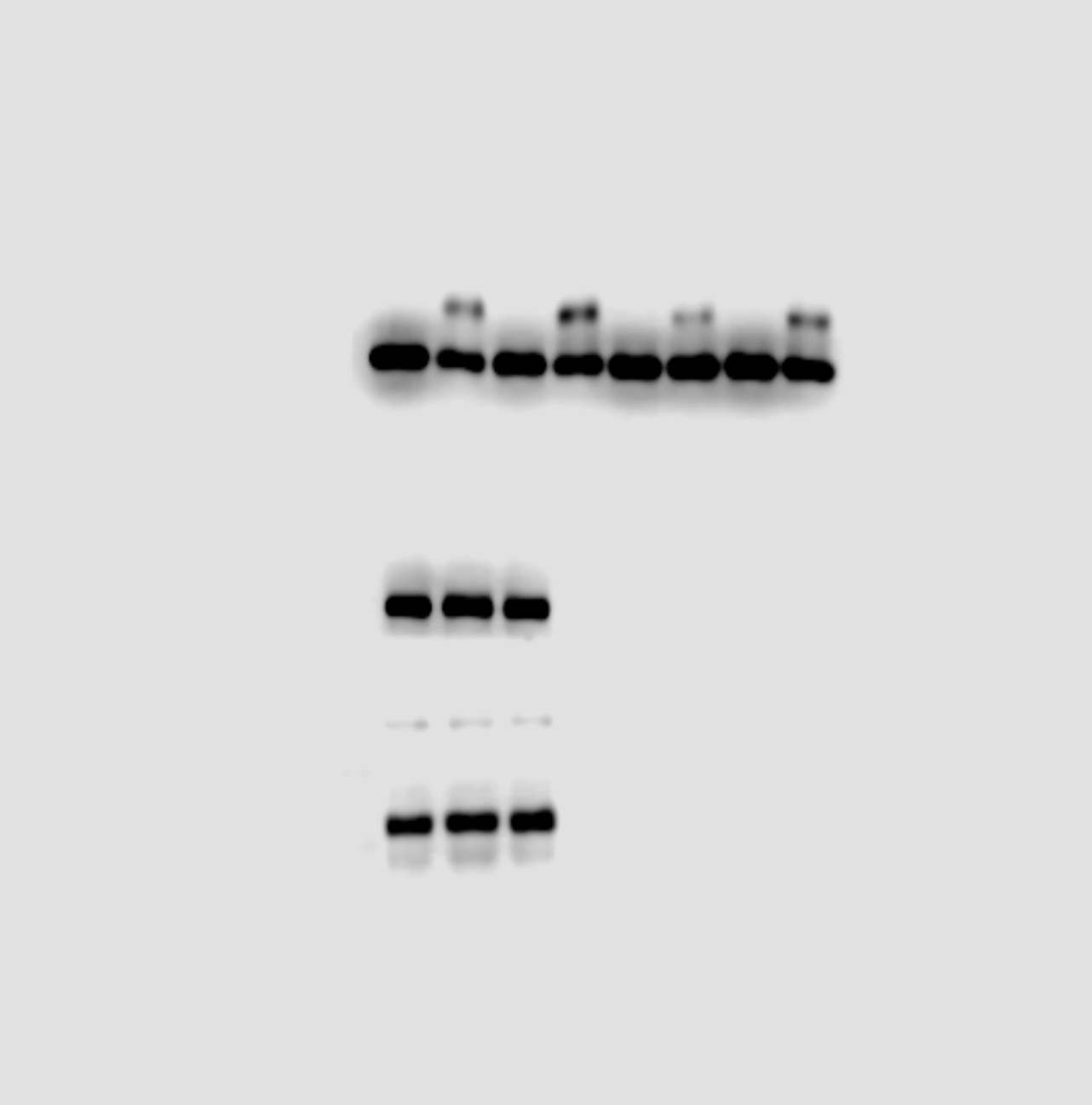

Supplement: Figure 1—source data 2. [file elife-99217-fig1-data2.zip › Figure 1, Source Data1/XRCC1 XRCC4.tif]

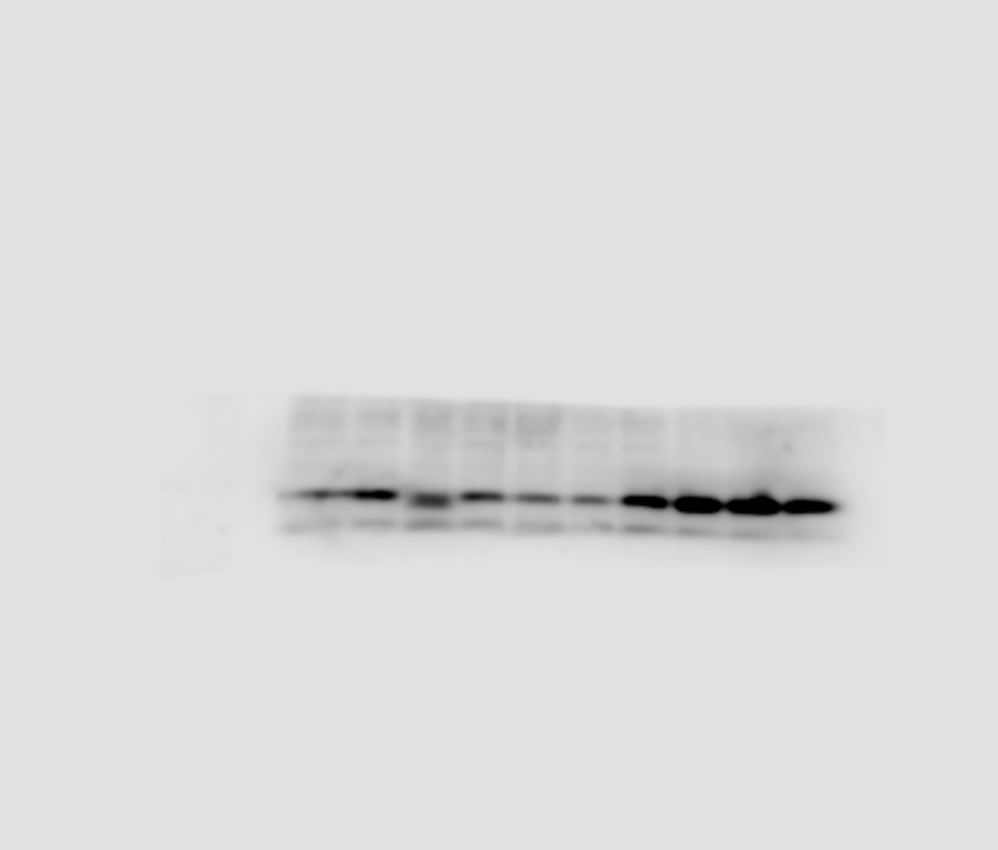

Supplement: Figure 1—figure supplement 2—source data 2. [file elife-99217-fig1-figsupp2-data2.zip › Figure 1-figure supplement 2A/gH2AX.tif]

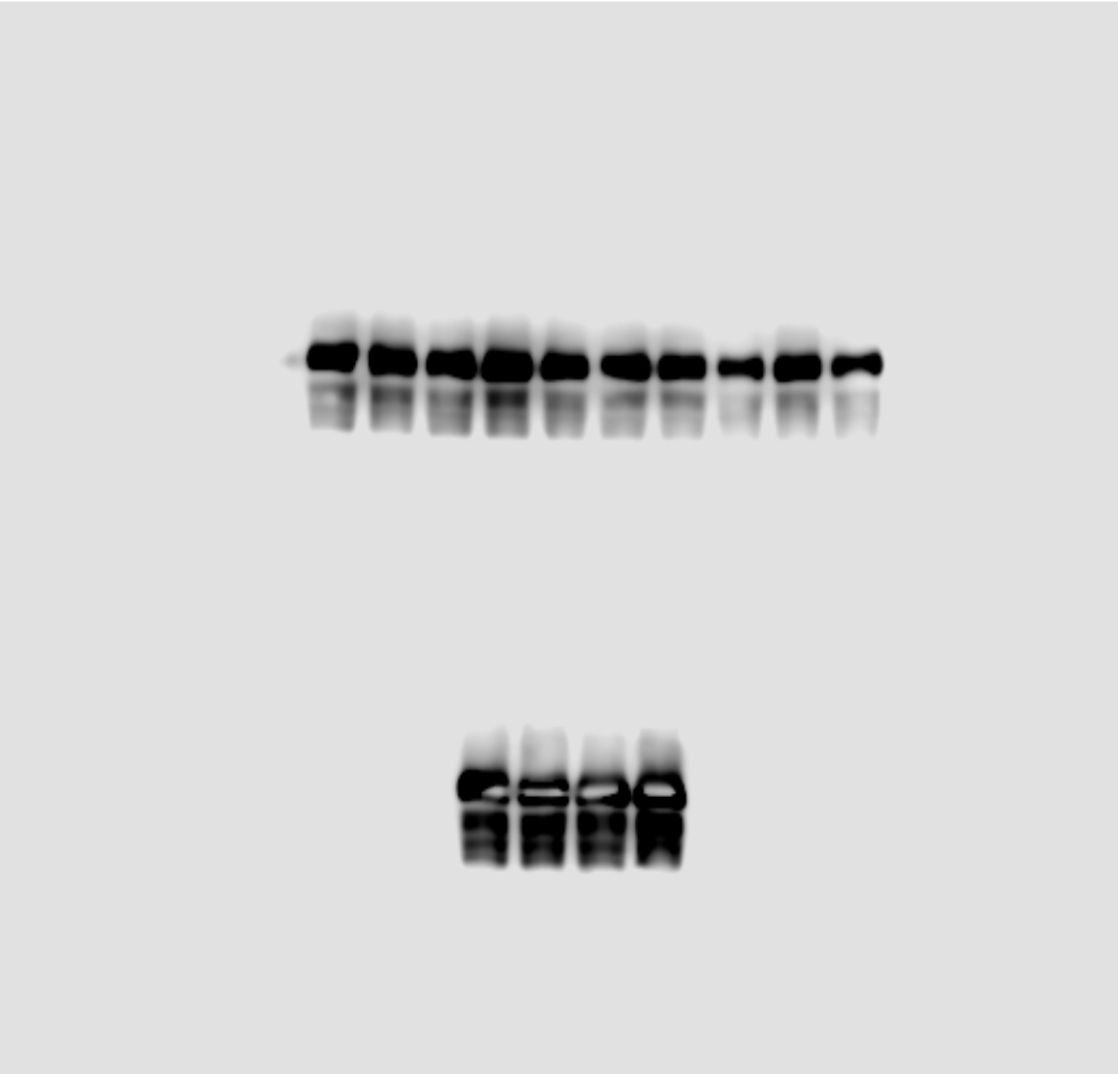

Supplement: Figure 1—figure supplement 2—source data 2. [file elife-99217-fig1-figsupp2-data2.zip › Figure 1-figure supplement 2A/KAP1.tif]

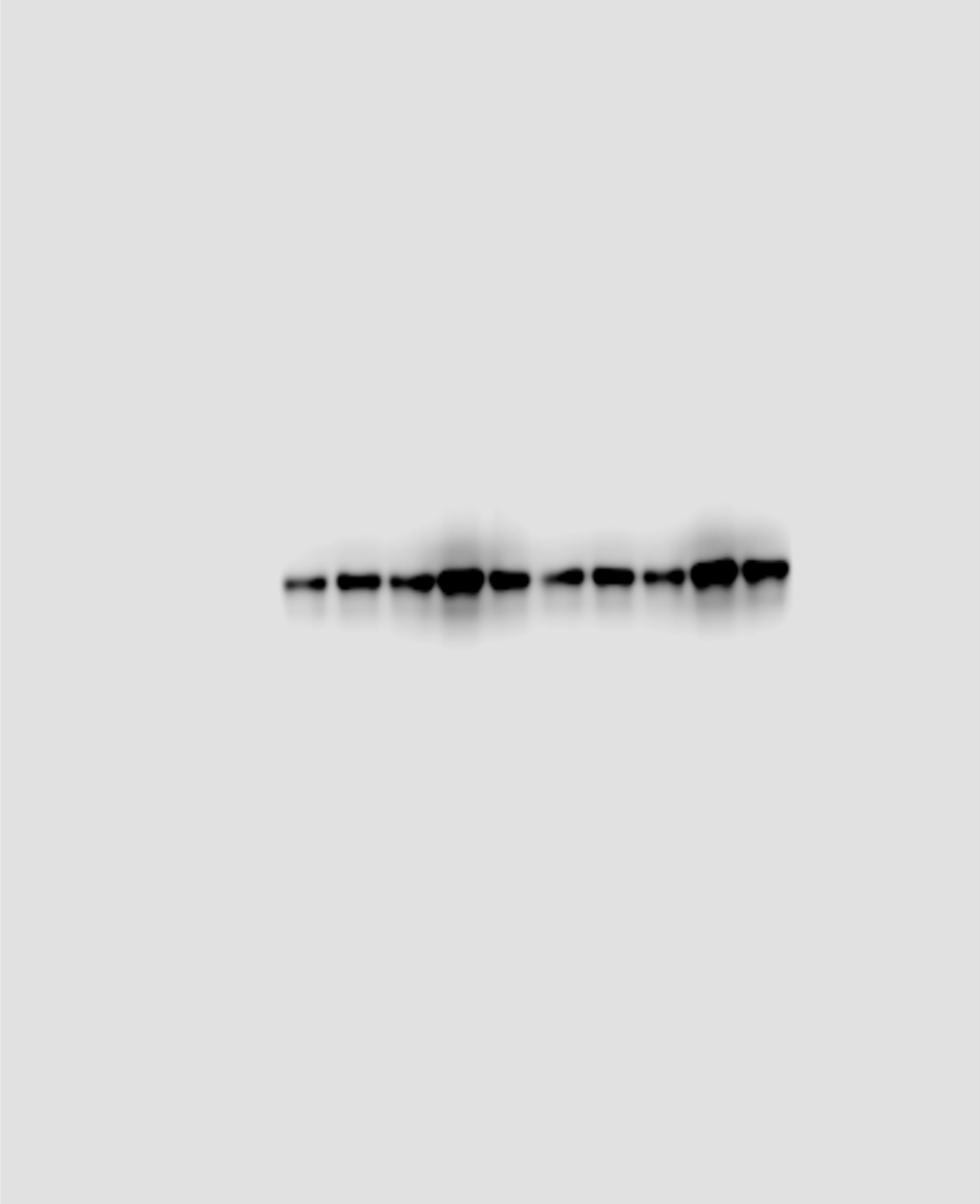

Supplement: Figure 1—figure supplement 2—source data 2. [file elife-99217-fig1-figsupp2-data2.zip › Figure 1-figure supplement 2A/p53.tif]

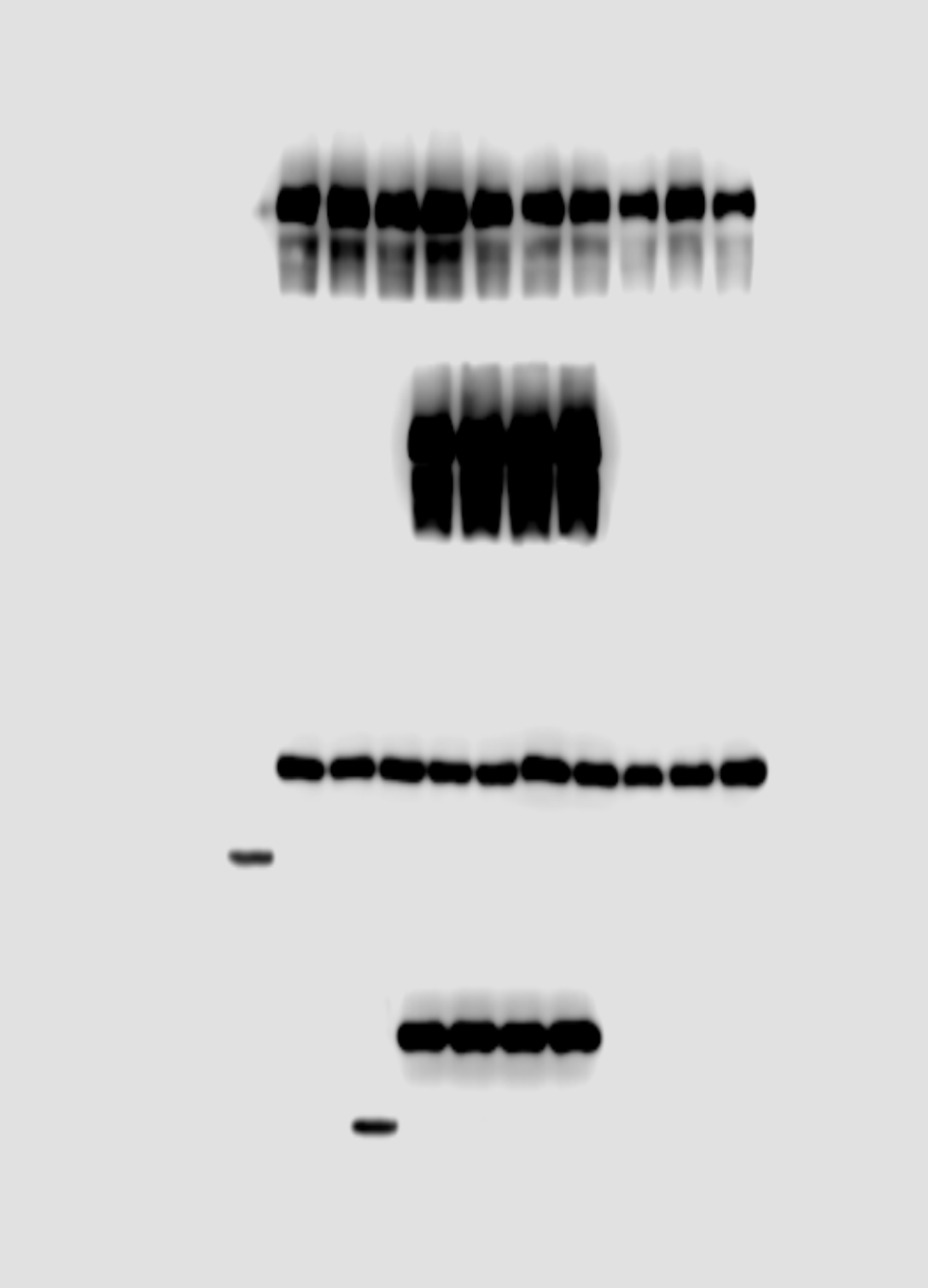

Supplement: Figure 1—figure supplement 2—source data 2. [file elife-99217-fig1-figsupp2-data2.zip › Figure 1-figure supplement 2A/PCNA.tif]

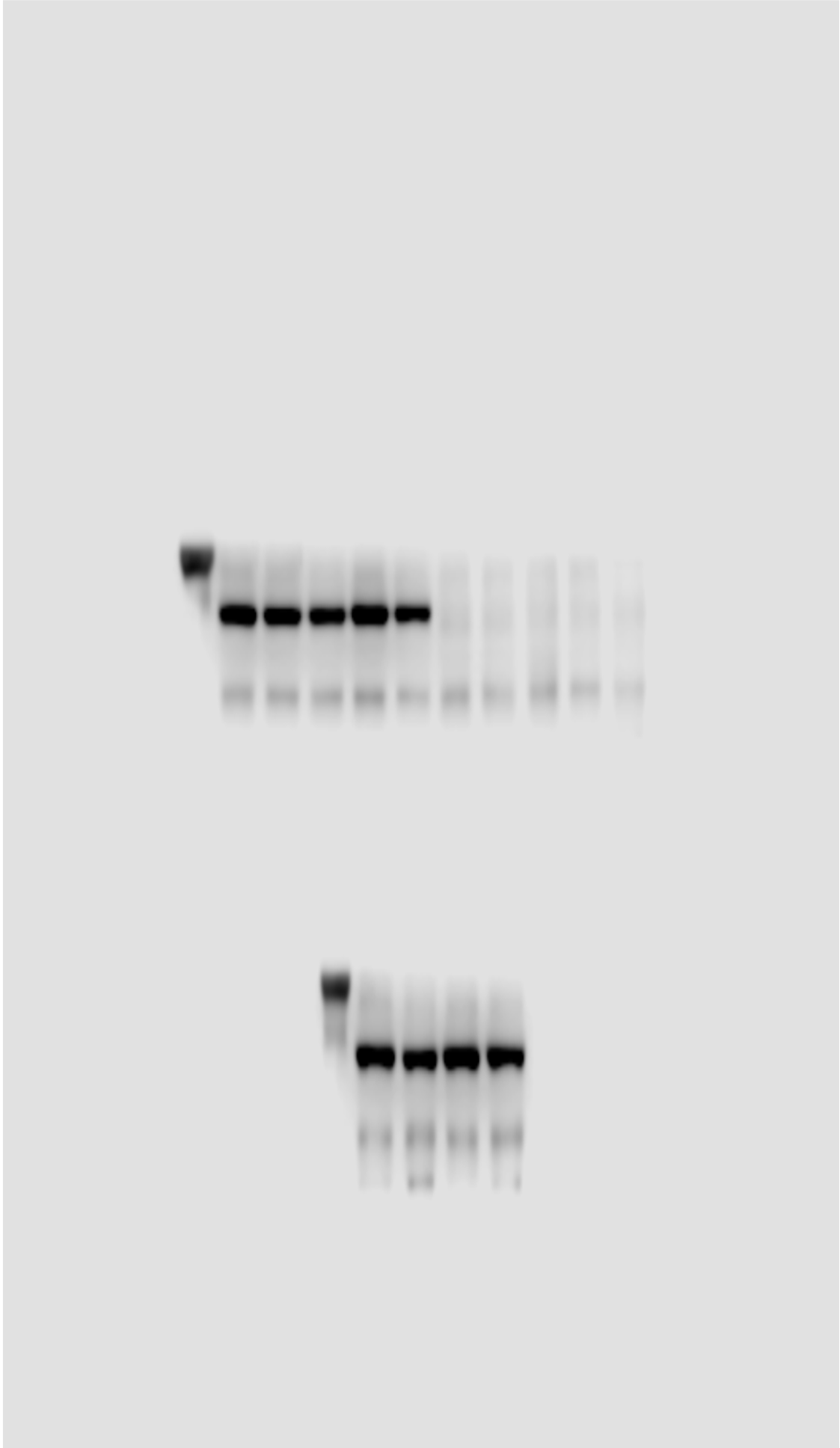

Supplement: Figure 1—figure supplement 2—source data 2. [file elife-99217-fig1-figsupp2-data2.zip › Figure 1-figure supplement 2A/PNKP.tif]

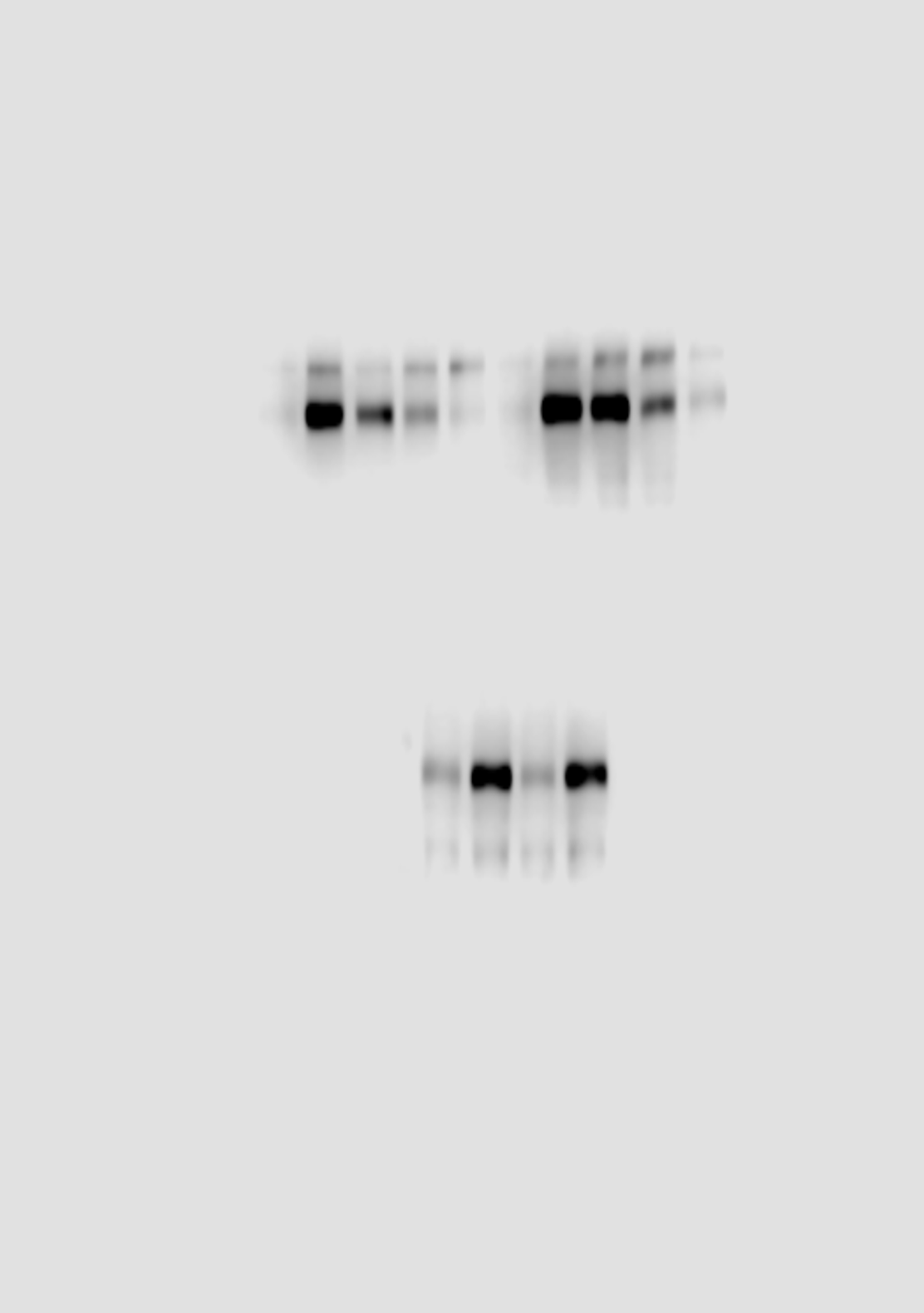

Supplement: Figure 1—figure supplement 2—source data 2. [file elife-99217-fig1-figsupp2-data2.zip › Figure 1-figure supplement 2A/pS824-KAP1.tif]

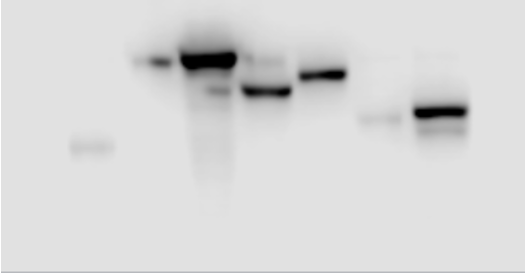

Supplement: Figure 3—figure supplement 1—source data 2. [file elife-99217-fig3-figsupp1-data2.zip › Figure 3-figure supplement A xπü«πé│πâÆπéÜπâ╝/PNKP.png]

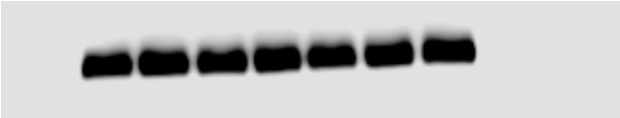

Supplement: Figure 3—figure supplement 1—source data 2. [file elife-99217-fig3-figsupp1-data2.zip › Figure 3-figure supplement A xπü«πé│πâÆπéÜπâ╝/KAP1.png]

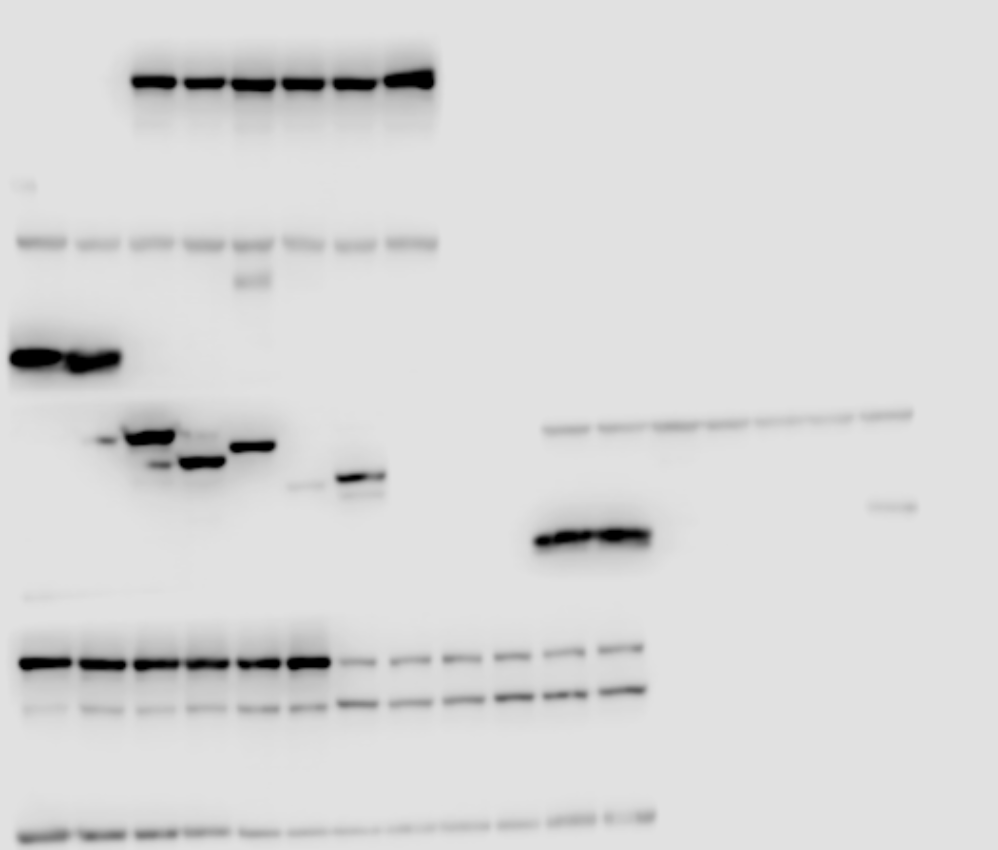

Supplement: Figure 3—figure supplement 1—source data 2. [file elife-99217-fig3-figsupp1-data2.zip › Figure 3-figure supplement A xπü«πé│πâÆπéÜπâ╝/GFP.png]

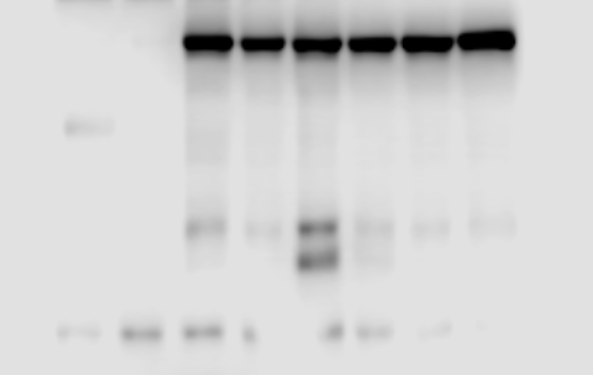

Supplement: Figure 3—figure supplement 1—source data 4. [file elife-99217-fig3-figsupp1-data4.zip › Figure 3-figure supplement B xπü«πé│πâÆπéÜπâ╝/PNKP.png]

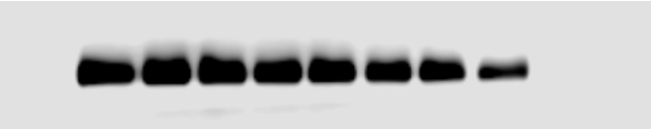

Supplement: Figure 3—figure supplement 1—source data 4. [file elife-99217-fig3-figsupp1-data4.zip › Figure 3-figure supplement B xπü«πé│πâÆπéÜπâ╝/KAP1.png]

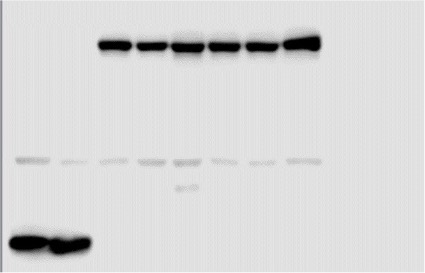

Supplement: Figure 3—figure supplement 1—source data 4. [file elife-99217-fig3-figsupp1-data4.zip › Figure 3-figure supplement B xπü«πé│πâÆπéÜπâ╝/GFP.jpg]

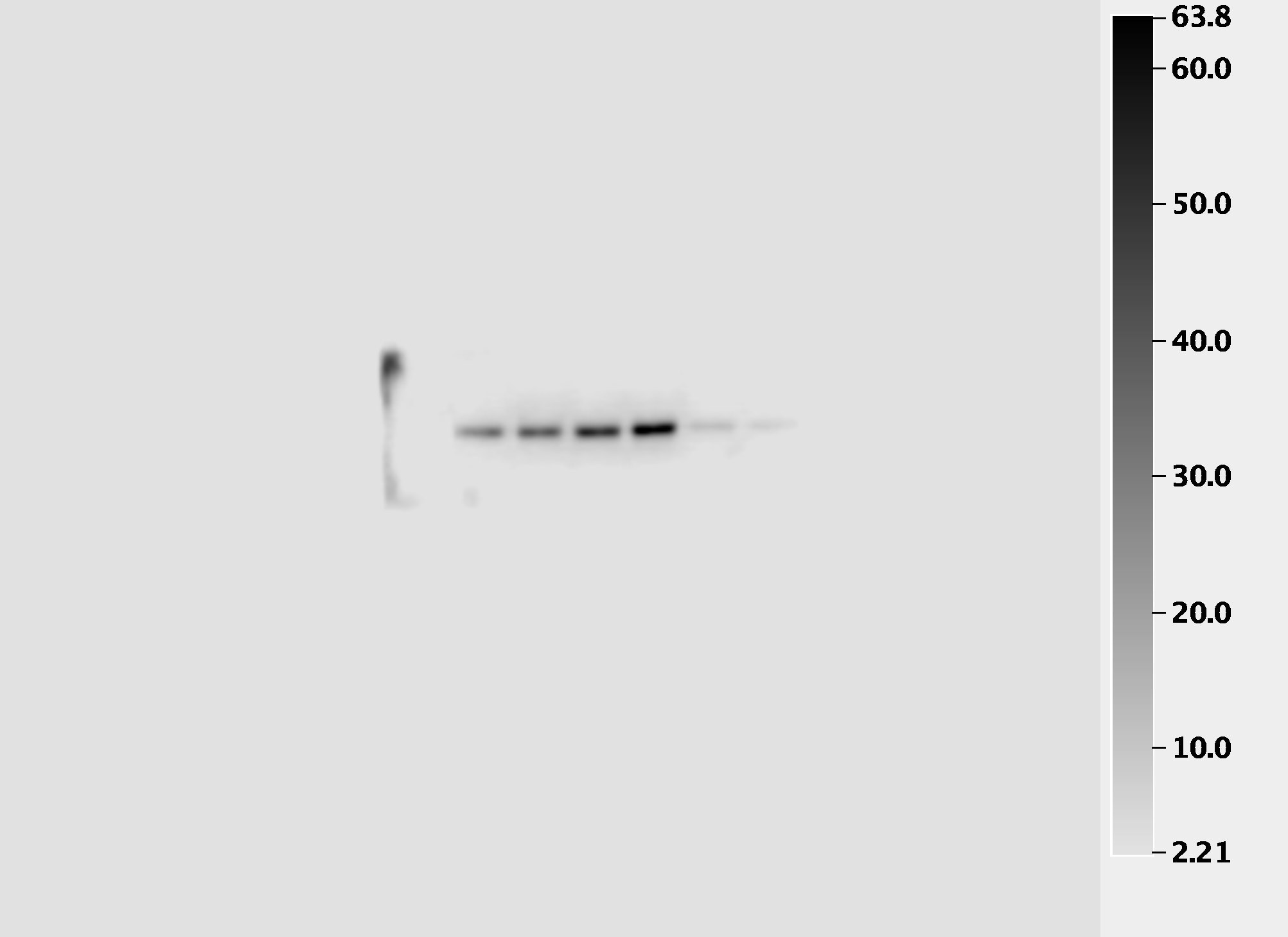

Supplement: Figure 4—source data 2. [file elife-99217-fig4-data2.zip › Figure 4Aπü«πé│πâÆπéÜπâ╝/CyclinA2.png]

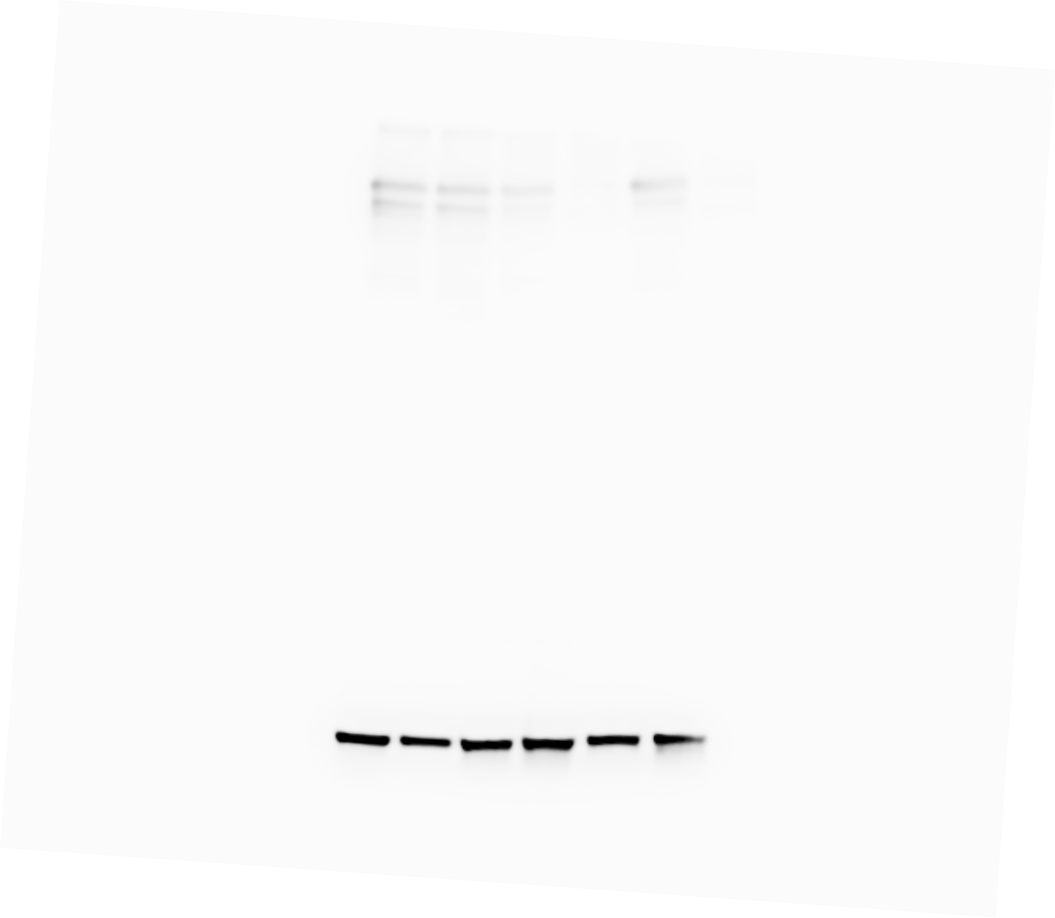

Supplement: Figure 4—source data 2. [file elife-99217-fig4-data2.zip › Figure 4Aπü«πé│πâÆπéÜπâ╝/GFP antibody.png]

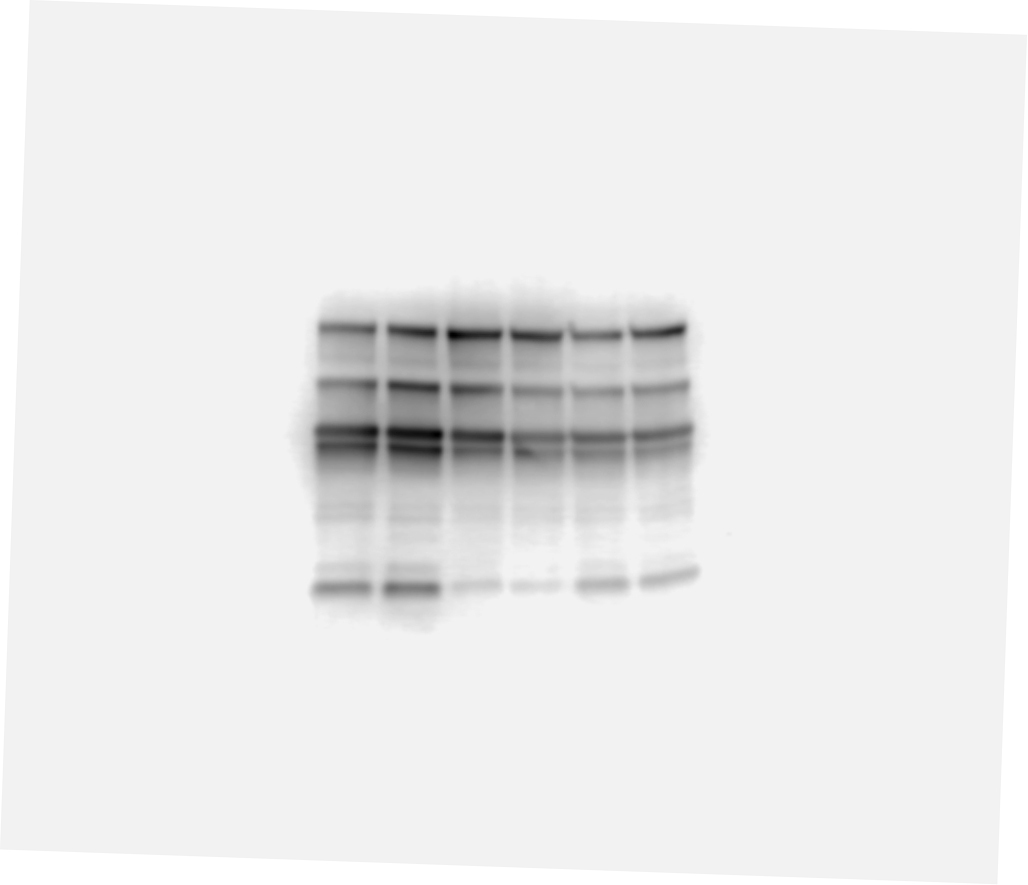

Supplement: Figure 4—source data 2. [file elife-99217-fig4-data2.zip › Figure 4Aπü«πé│πâÆπéÜπâ╝/PNKP antibody.jpg]

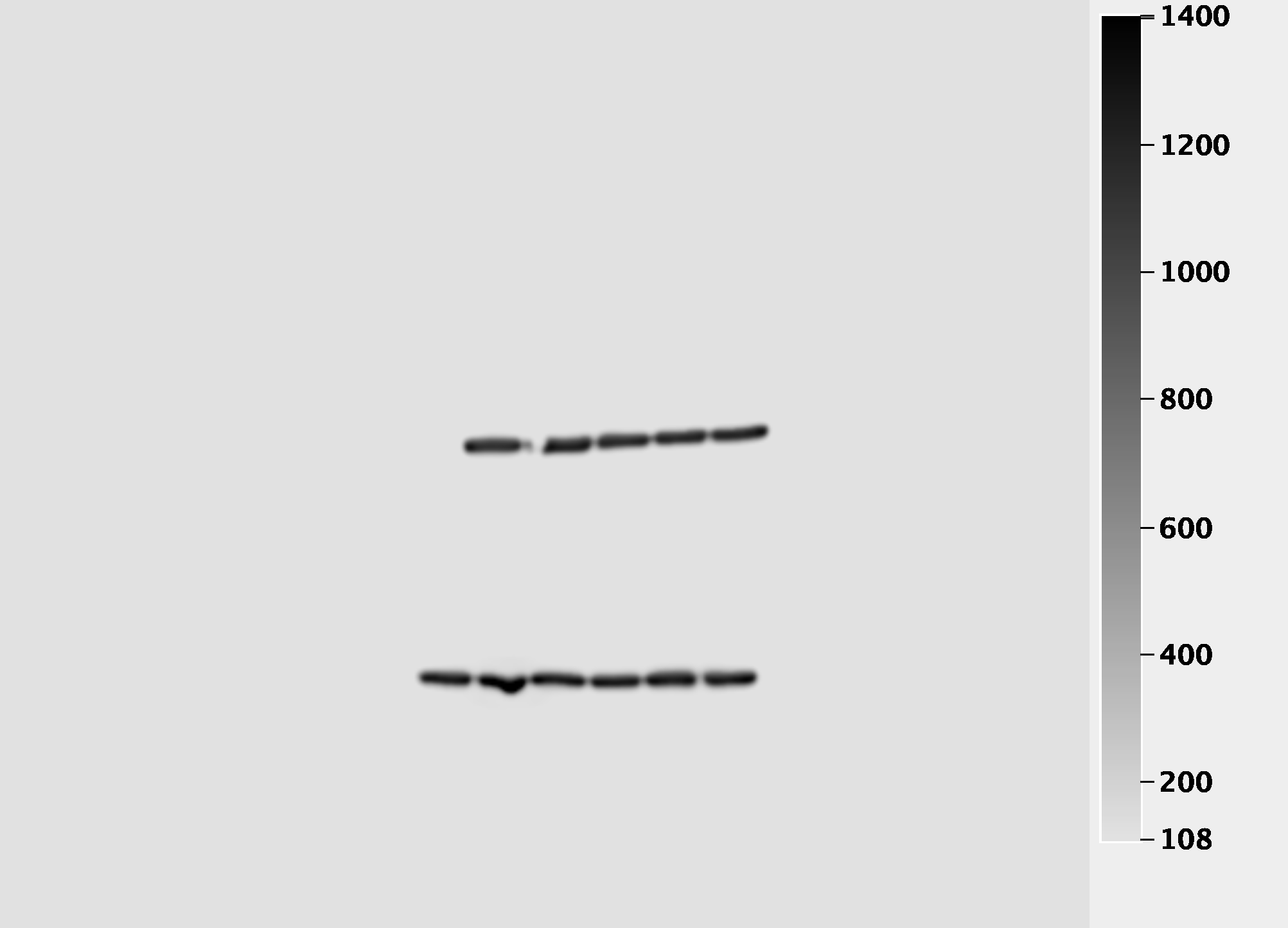

Supplement: Figure 4—source data 2. [file elife-99217-fig4-data2.zip › Figure 4Aπü«πé│πâÆπéÜπâ╝/GAPDH (bottom).png]

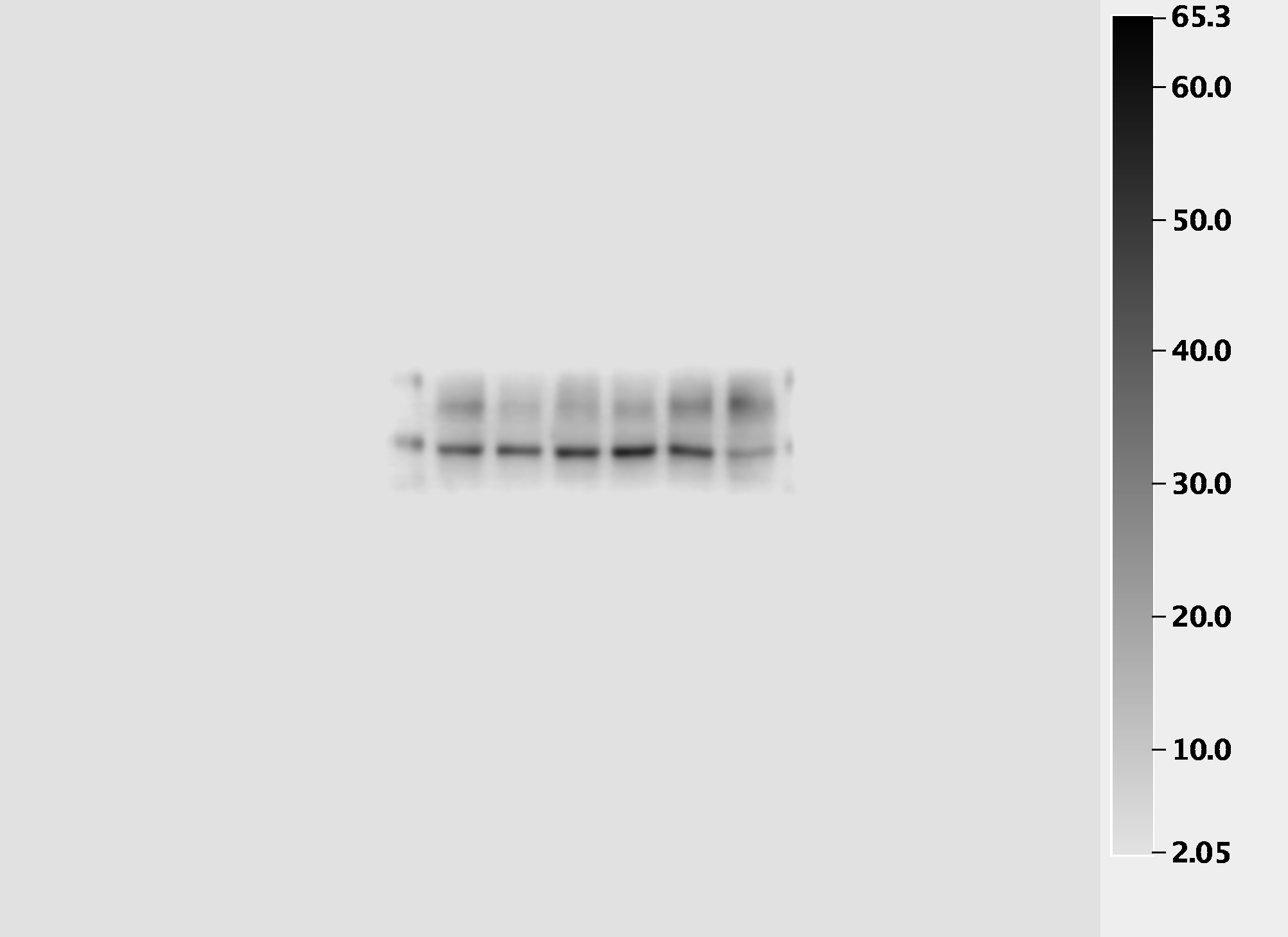

Supplement: Figure 4—source data 2. [file elife-99217-fig4-data2.zip › Figure 4Aπü«πé│πâÆπéÜπâ╝/pT118.png]

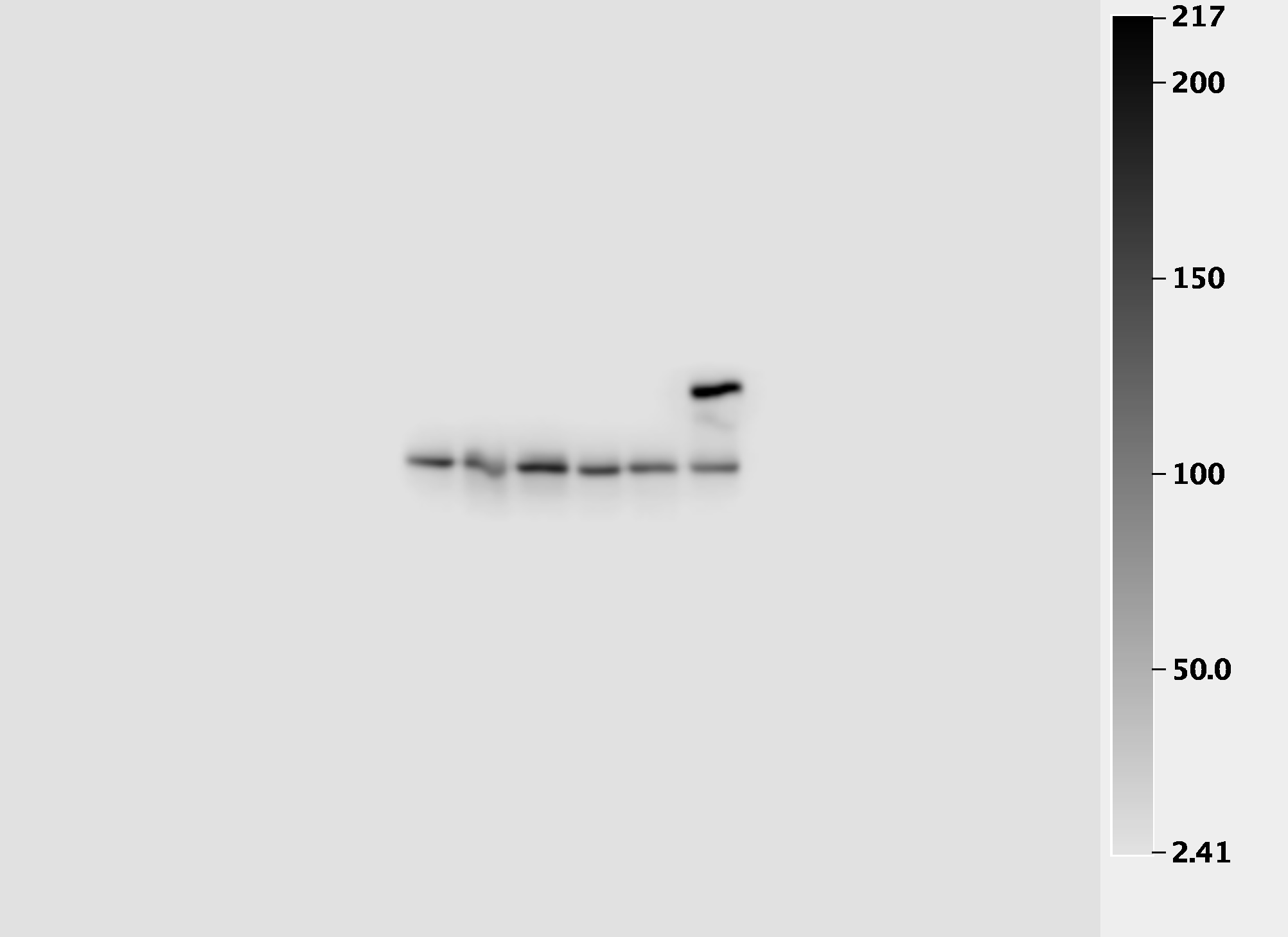

Supplement: Figure 4—source data 2. [file elife-99217-fig4-data2.zip › Figure 4Aπü«πé│πâÆπéÜπâ╝/CyclinE1.png]

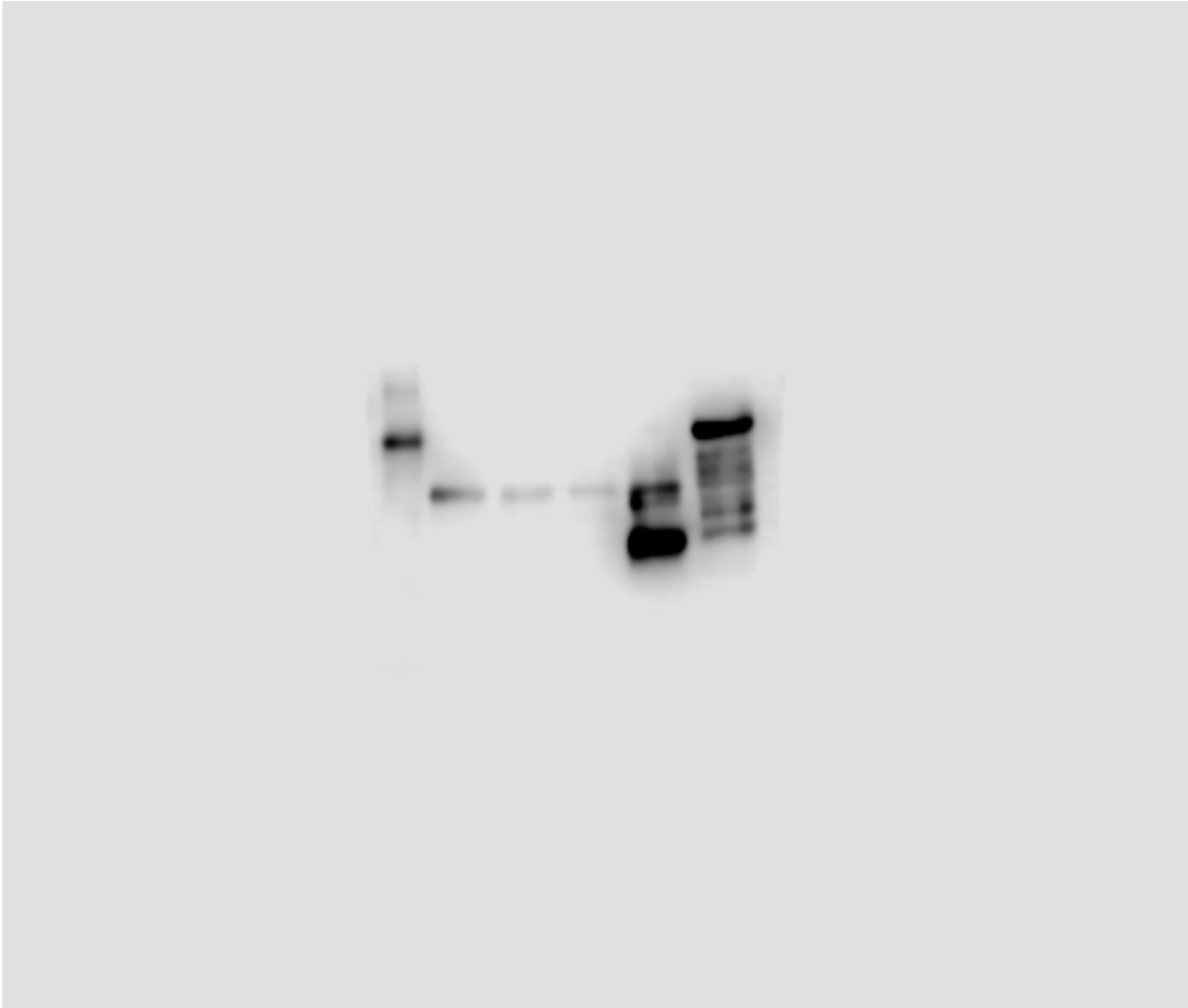

Supplement: Figure 4—source data 4. [file elife-99217-fig4-data4.zip › Figure 4B, Souce Data2/Cyclin A2.tif]

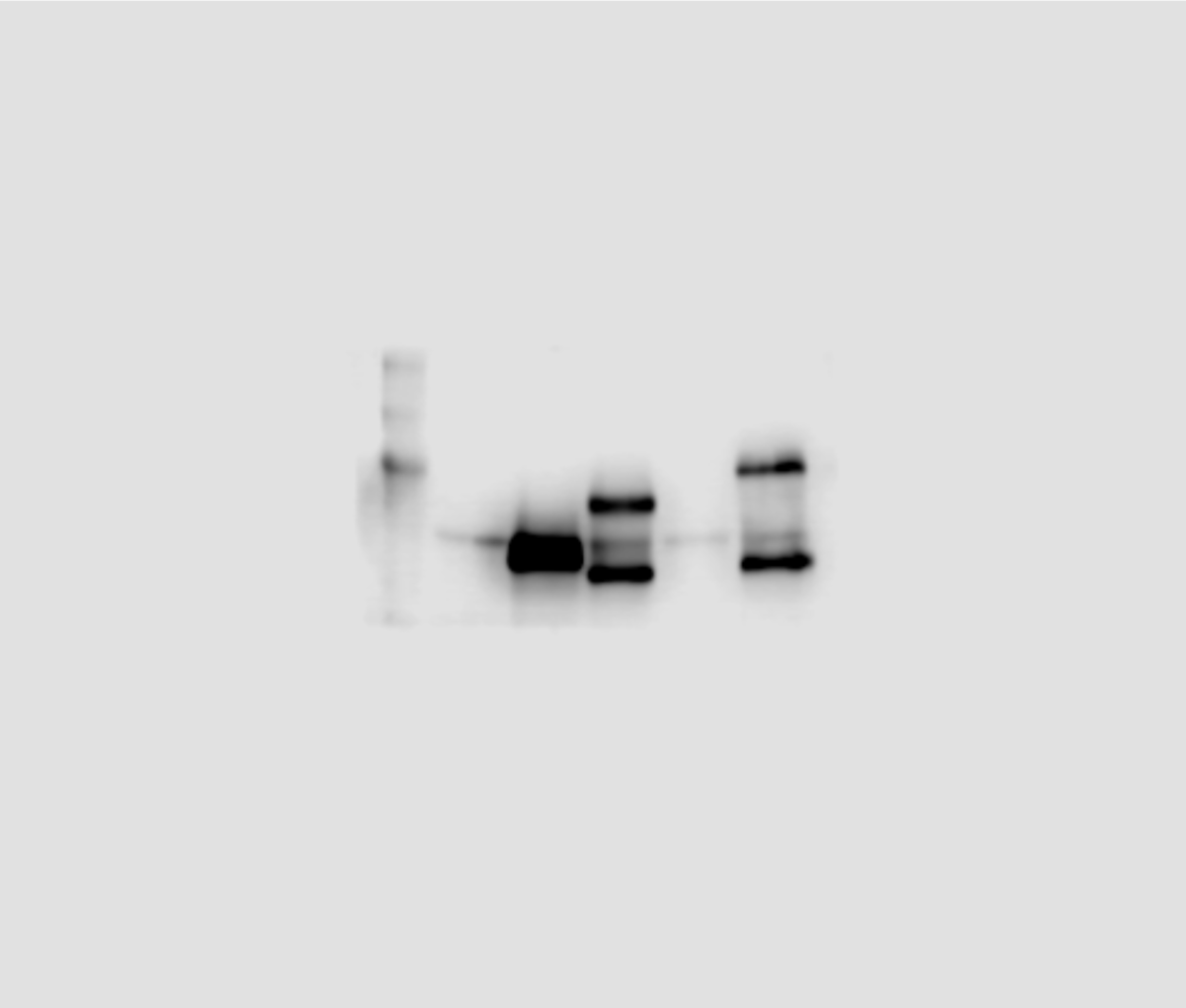

Supplement: Figure 4—source data 4. [file elife-99217-fig4-data4.zip › Figure 4B, Souce Data2/GST.tif]

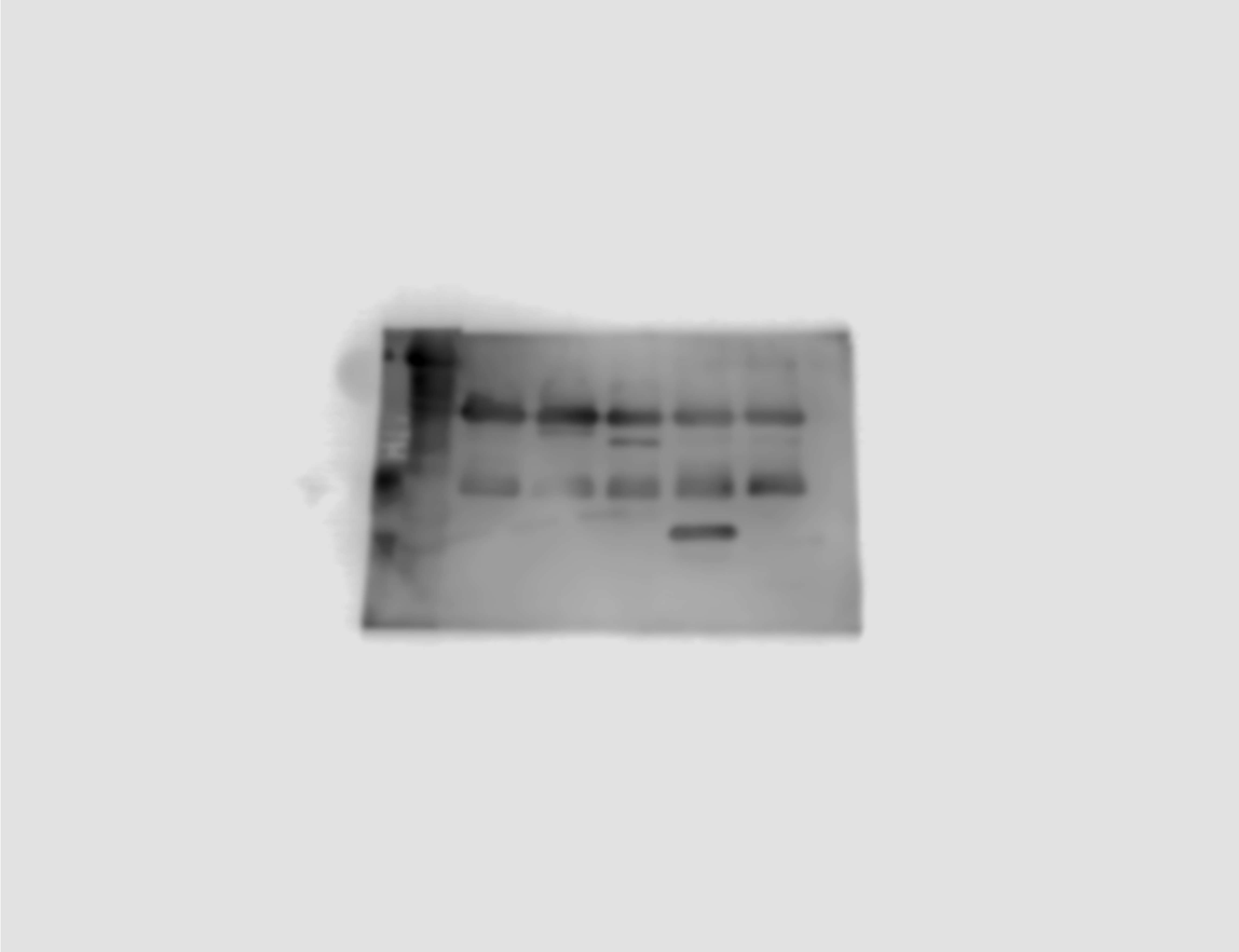

Supplement: Figure 4—source data 4. [file elife-99217-fig4-data4.zip › Figure 4B, Souce Data2/His.tif]

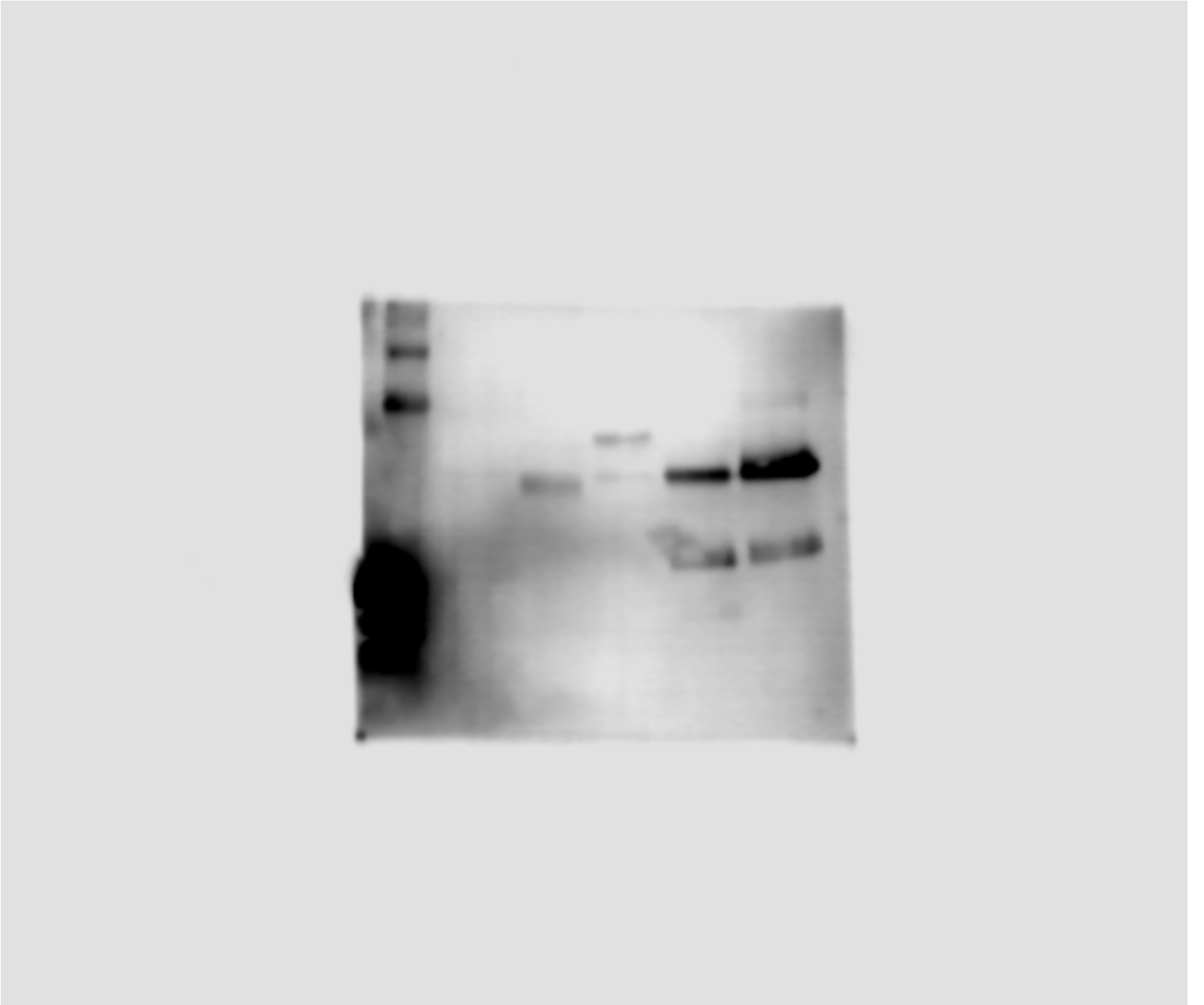

Supplement: Figure 4—source data 4. [file elife-99217-fig4-data4.zip › Figure 4B, Souce Data2/pT118-PNKP.tif]

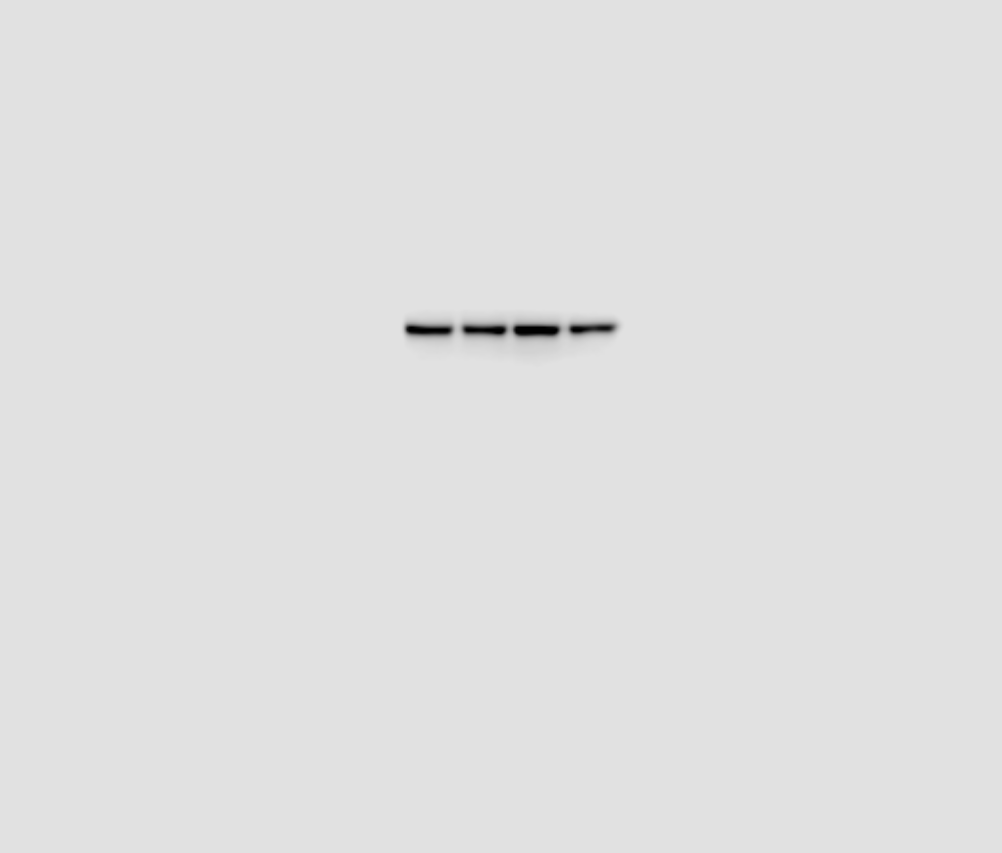

Supplement: Figure 4—source data 6. [file elife-99217-fig4-data6.zip › Figure 4Cπü«πé│πâÆπéÜπâ╝/PNKP_ GFP-PNKP.png]

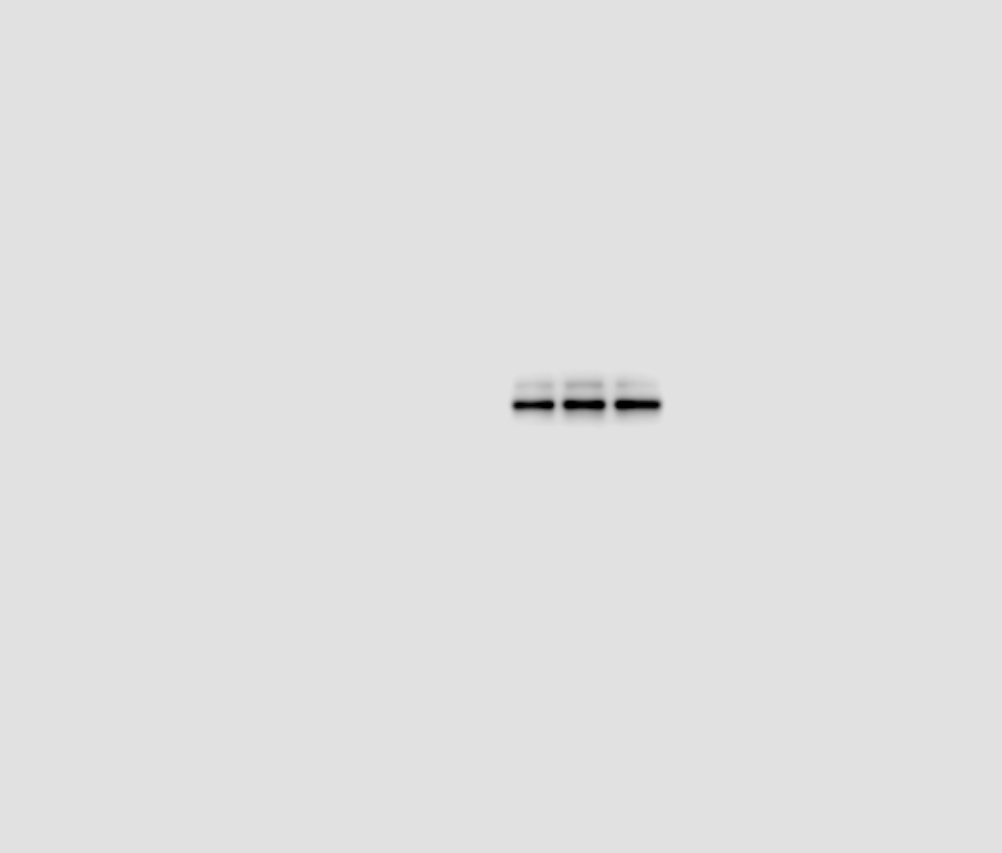

Supplement: Figure 4—source data 6. [file elife-99217-fig4-data6.zip › Figure 4Cπü«πé│πâÆπéÜπâ╝/mCherry2-CCNA2.png]

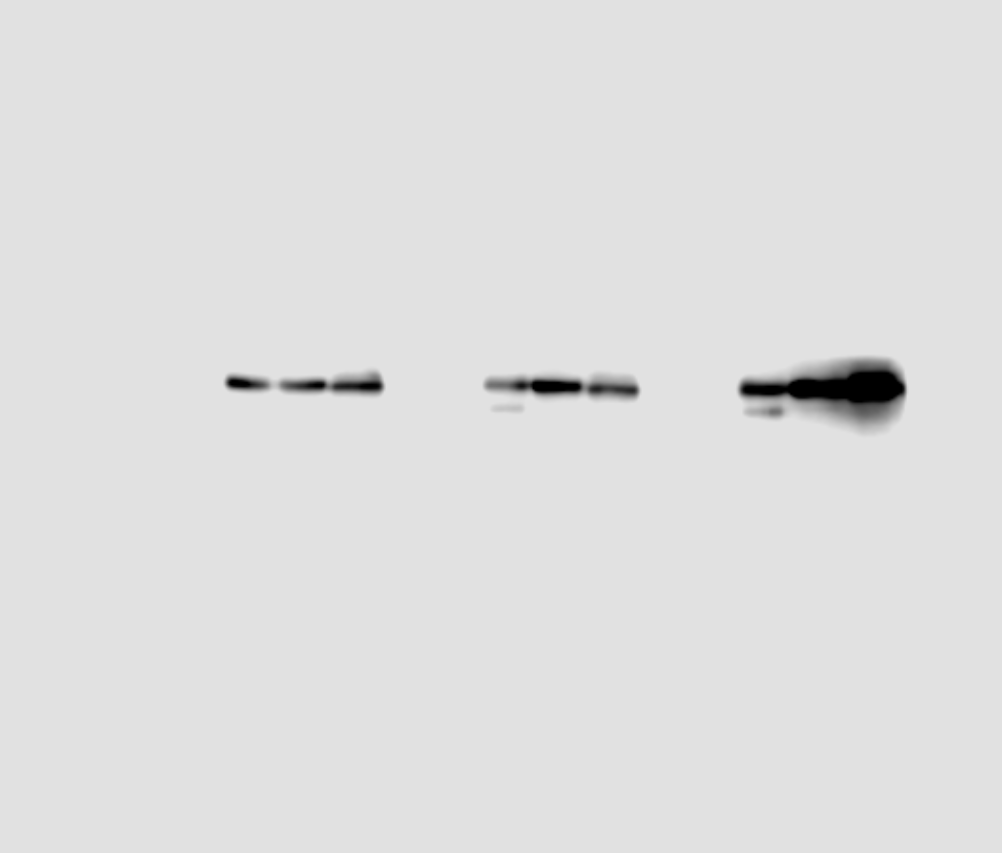

Supplement: Figure 4—source data 6. [file elife-99217-fig4-data6.zip › Figure 4Cπü«πé│πâÆπéÜπâ╝/FLAG-CDKs.png]

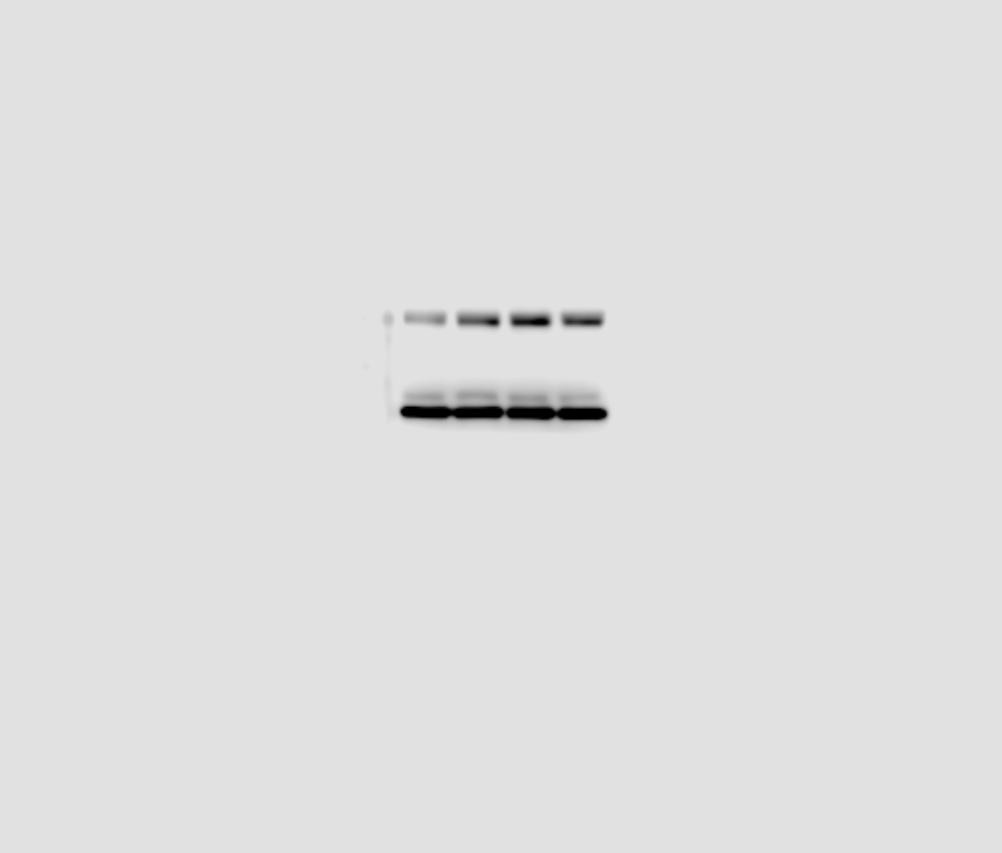

Supplement: Figure 4—source data 6. [file elife-99217-fig4-data6.zip › Figure 4Cπü«πé│πâÆπéÜπâ╝/pT118_GFP-PNKP.png]

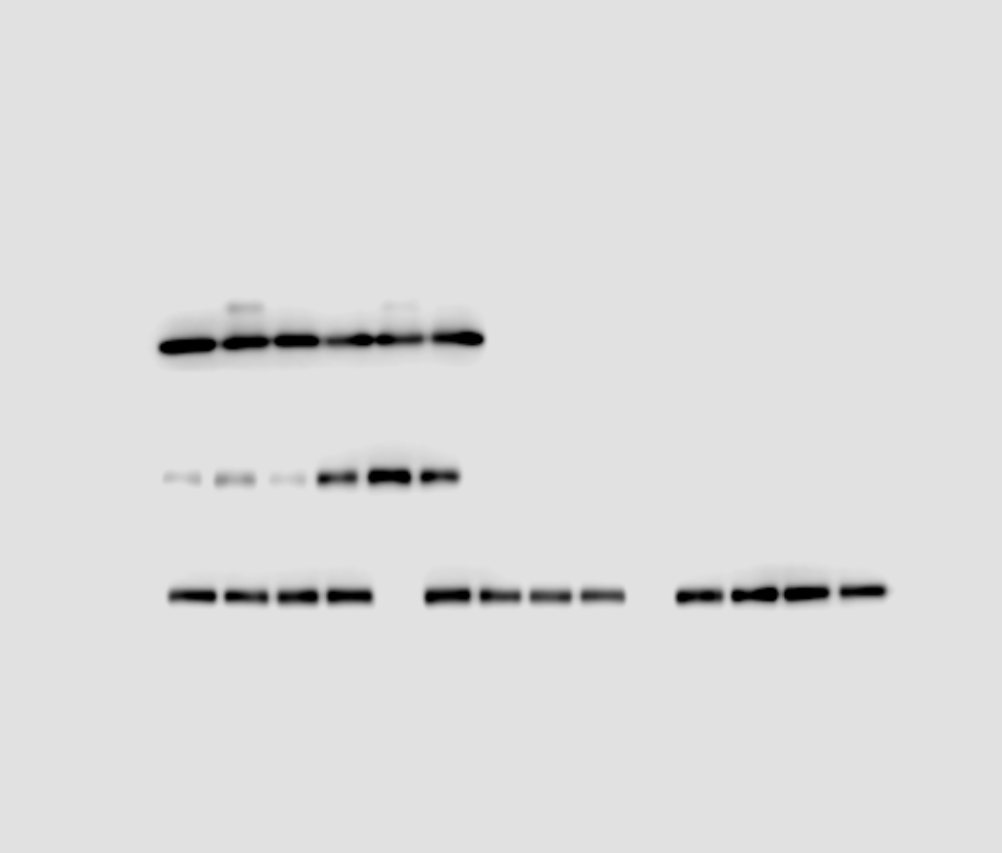

Supplement: Figure 4—source data 6. [file elife-99217-fig4-data6.zip › Figure 4Cπü«πé│πâÆπéÜπâ╝/KAP1.png]

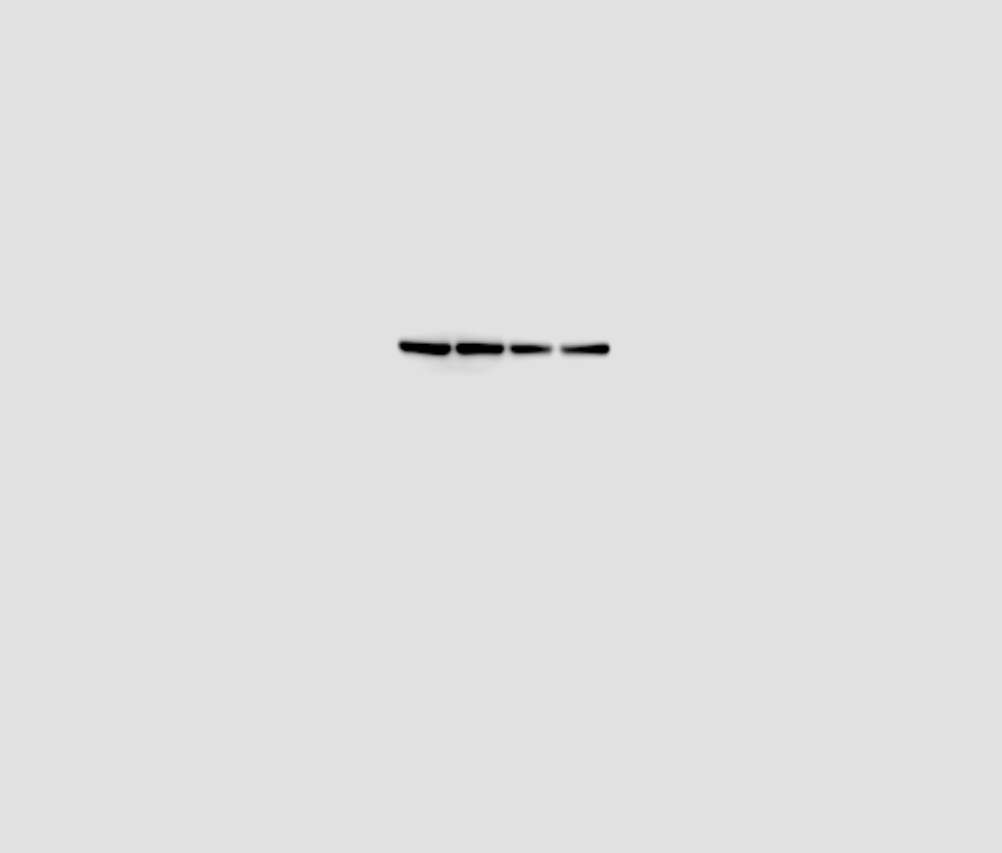

Supplement: Figure 4—source data 6. [file elife-99217-fig4-data6.zip › Figure 4Cπü«πé│πâÆπéÜπâ╝/GFP_ GFP-PNKP.png]

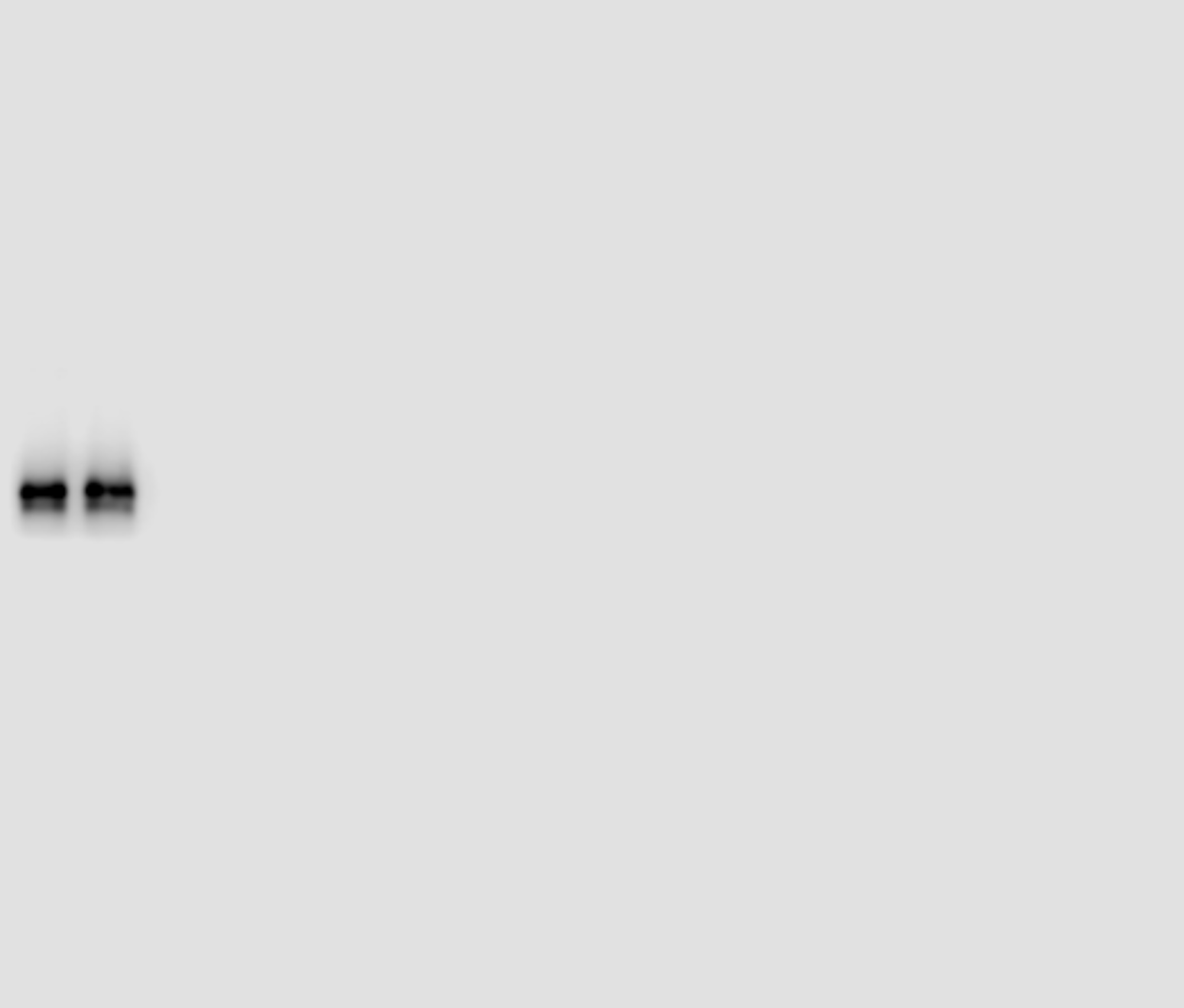

Supplement: Figure 4—source data 8. [file elife-99217-fig4-data8.zip › Figure 4Dπü«πé│πâÆπéÜπâ╝/RPA2 input.tif]

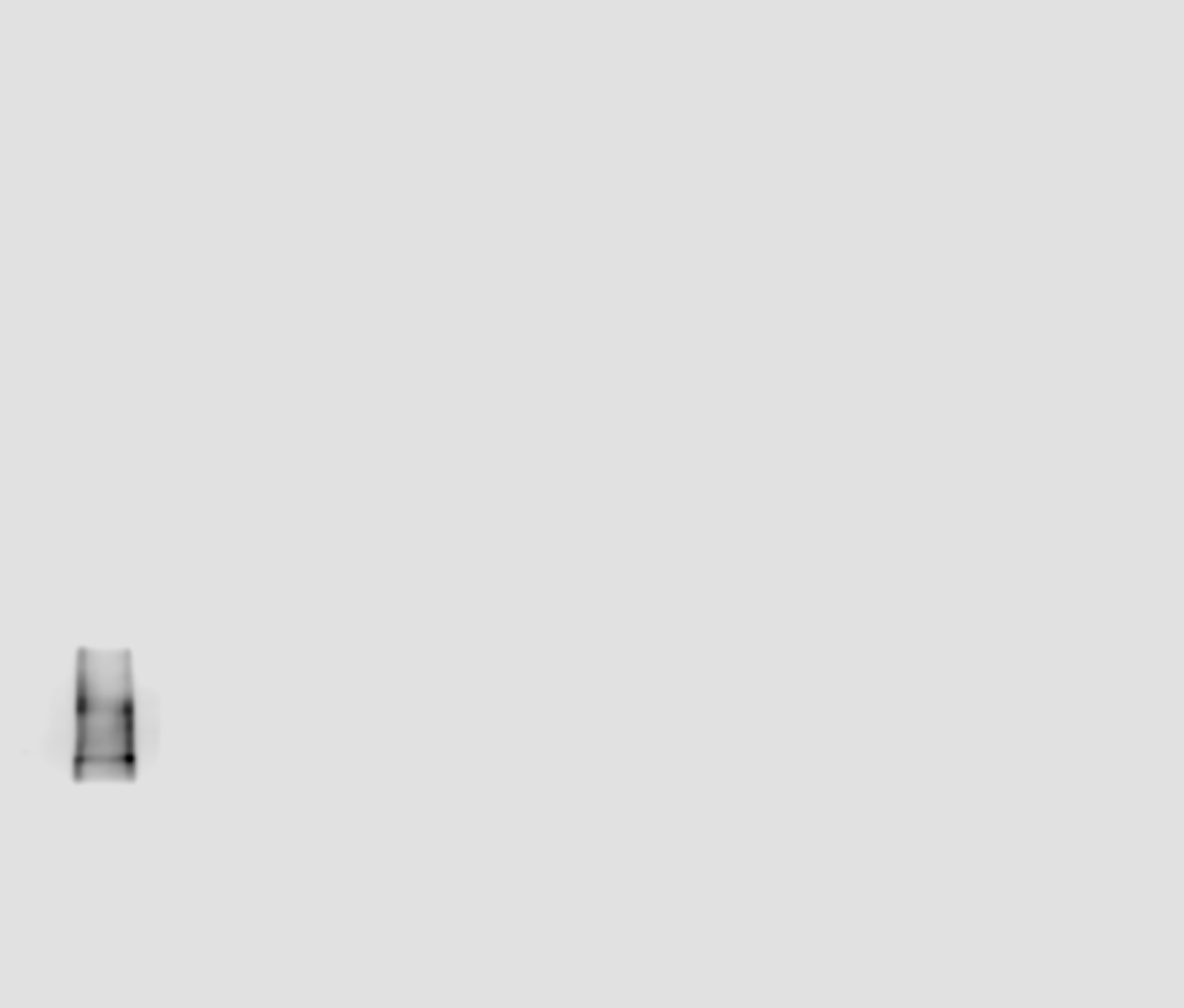

Supplement: Figure 4—source data 8. [file elife-99217-fig4-data8.zip › Figure 4Dπü«πé│πâÆπéÜπâ╝/PNKP pT118 iPOND.png]

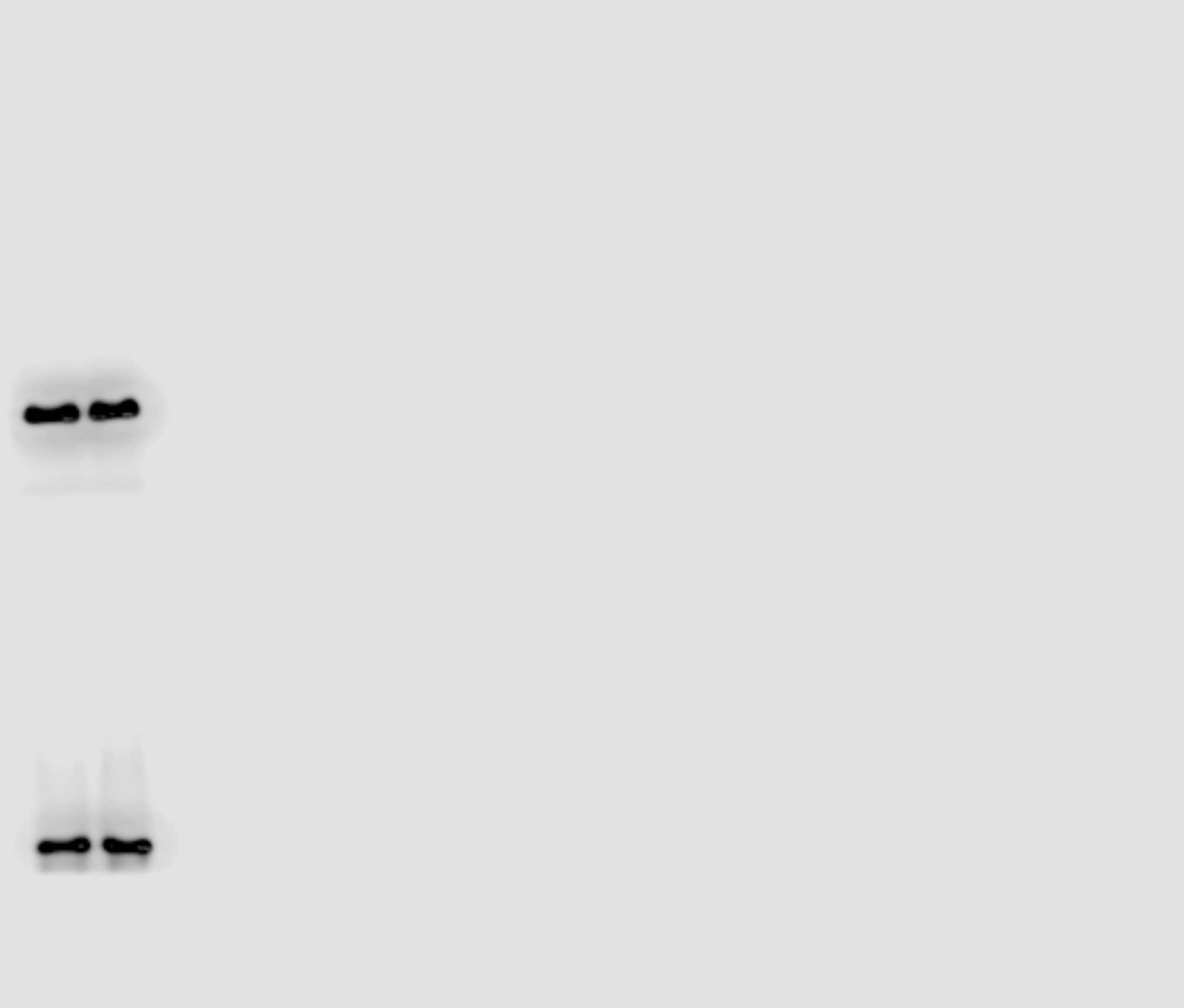

Supplement: Figure 4—source data 8. [file elife-99217-fig4-data8.zip › Figure 4Dπü«πé│πâÆπéÜπâ╝/FEN1 PCNA input.png]

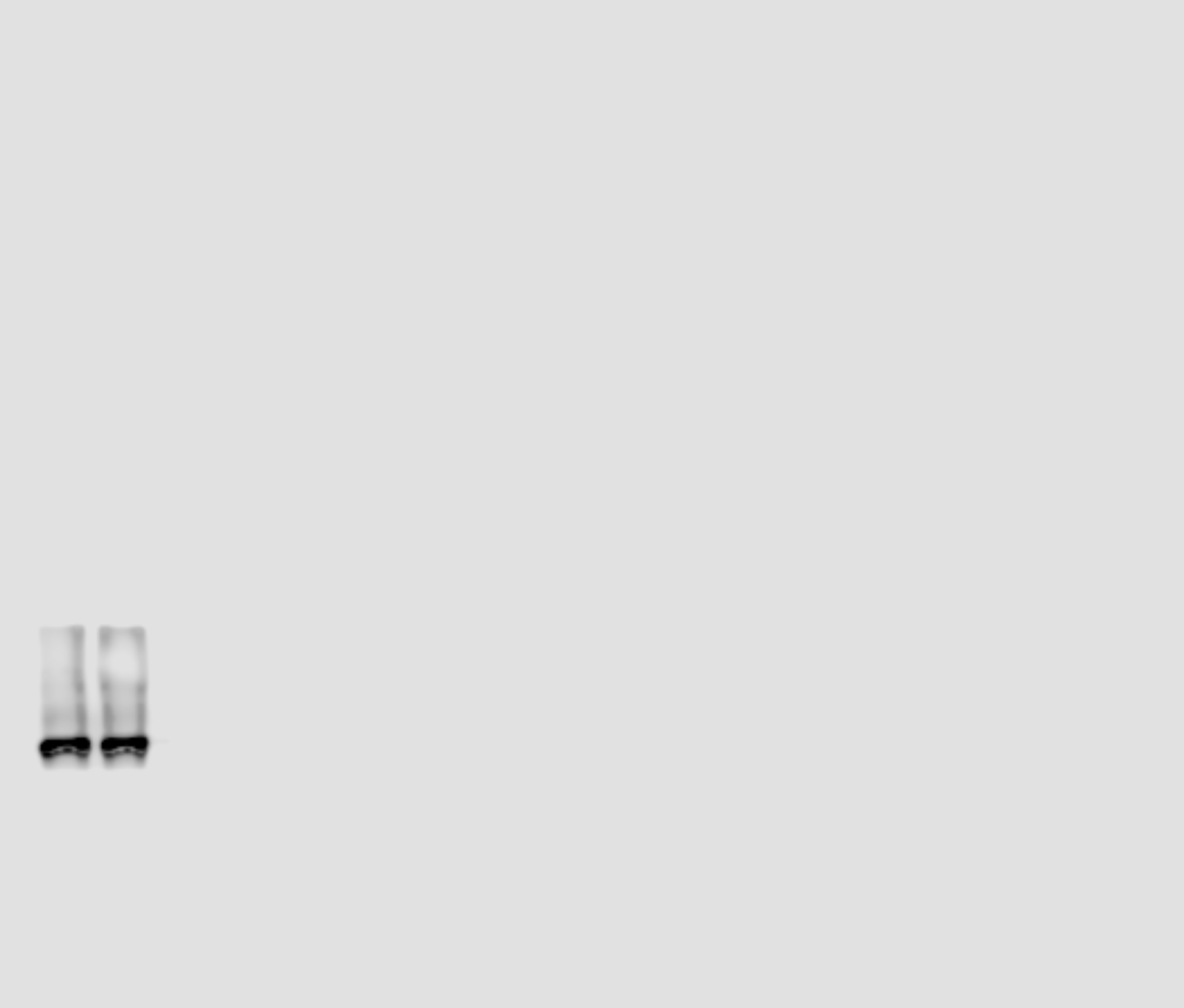

Supplement: Figure 4—source data 8. [file elife-99217-fig4-data8.zip › Figure 4Dπü«πé│πâÆπéÜπâ╝/GFP-PNKP input.tif]

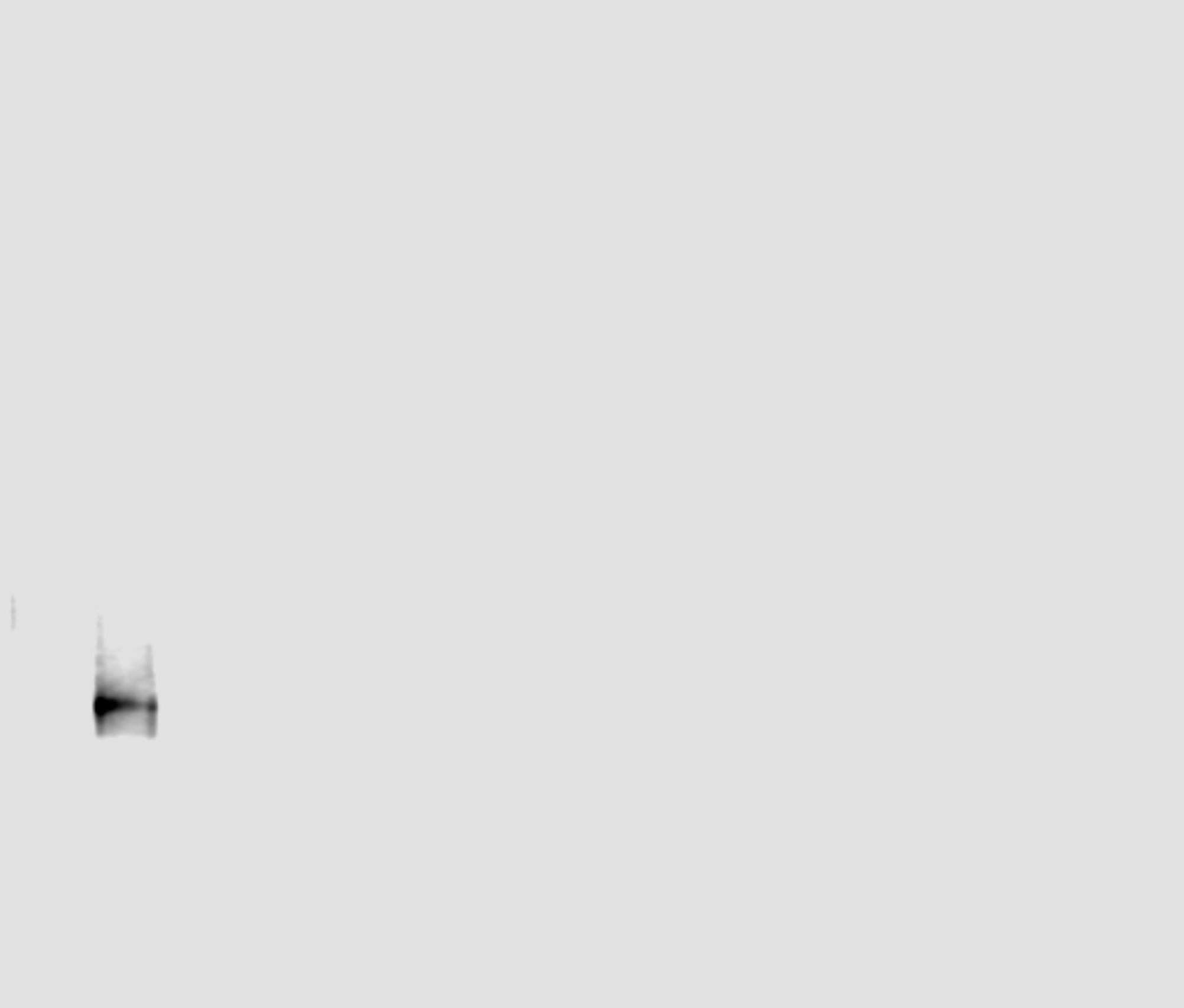

Supplement: Figure 4—source data 8. [file elife-99217-fig4-data8.zip › Figure 4Dπü«πé│πâÆπéÜπâ╝/FEN1 iPOND.png]

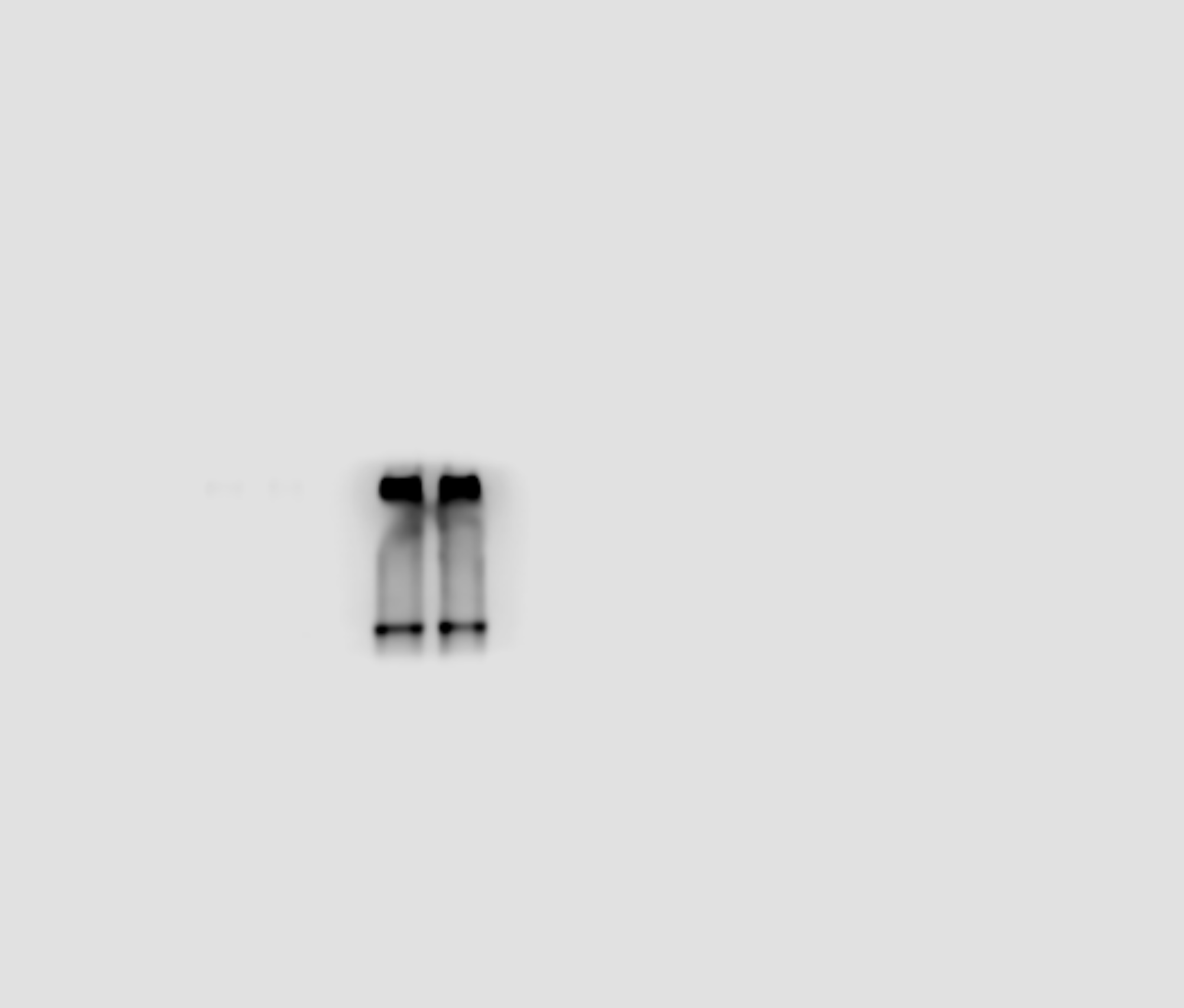

Supplement: Figure 4—source data 8. [file elife-99217-fig4-data8.zip › Figure 4Dπü«πé│πâÆπéÜπâ╝/PNKP-GFP-PNKP input.tif]

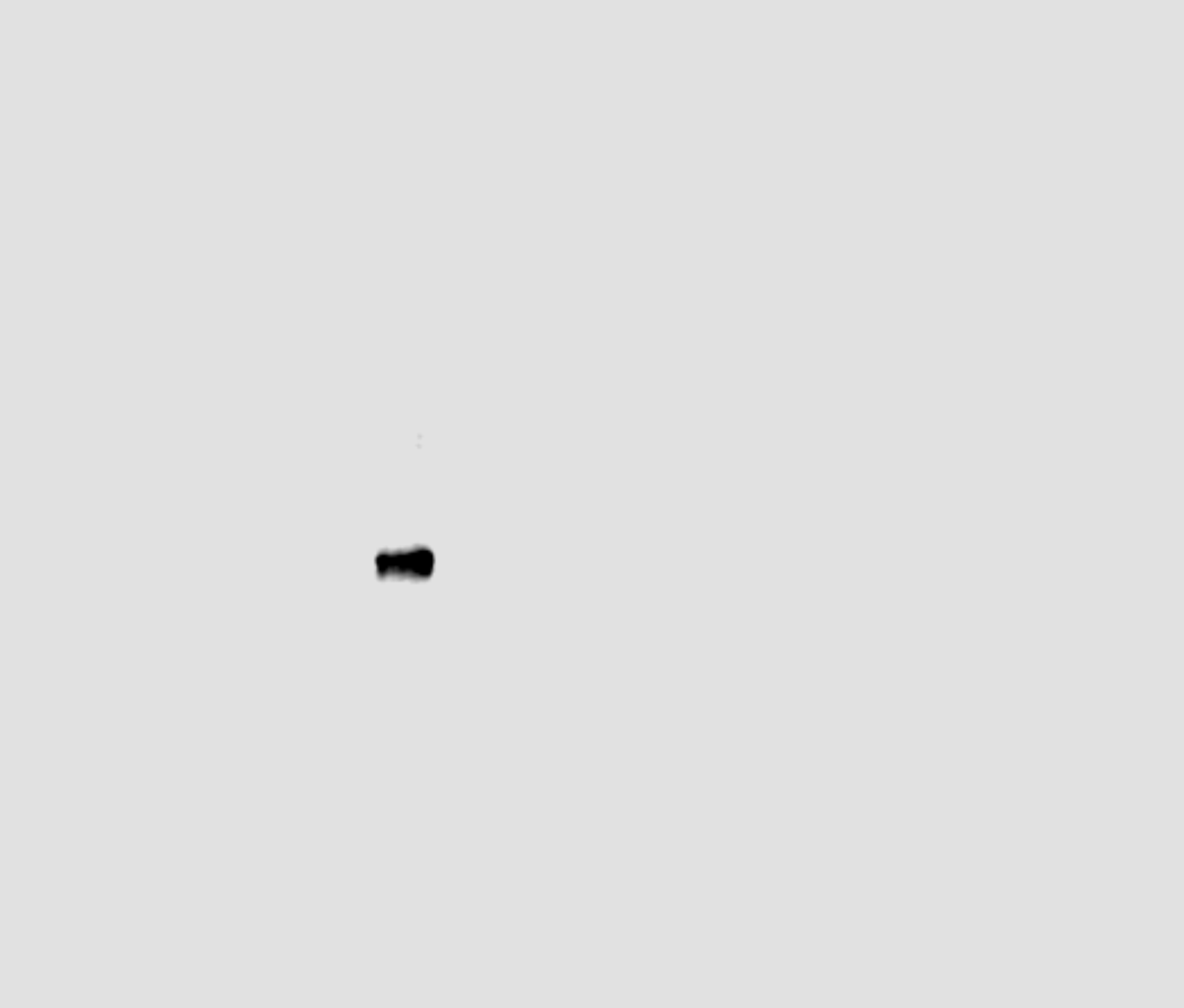

Supplement: Figure 4—source data 8. [file elife-99217-fig4-data8.zip › Figure 4Dπü«πé│πâÆπéÜπâ╝/PNKP-GFP-PNKP iPOND.png]

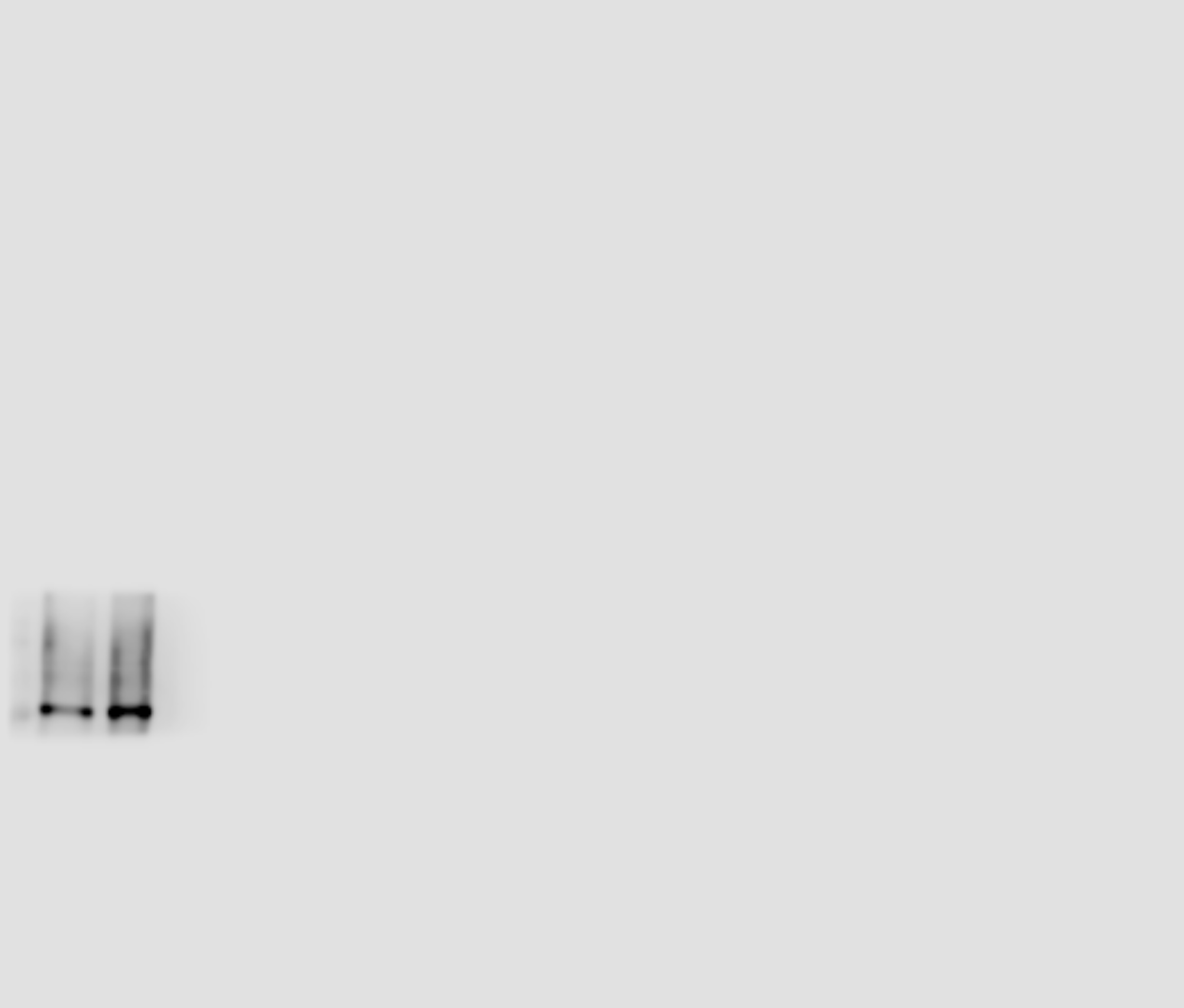

Supplement: Figure 4—source data 8. [file elife-99217-fig4-data8.zip › Figure 4Dπü«πé│πâÆπéÜπâ╝/PNKP pT118 input.png]

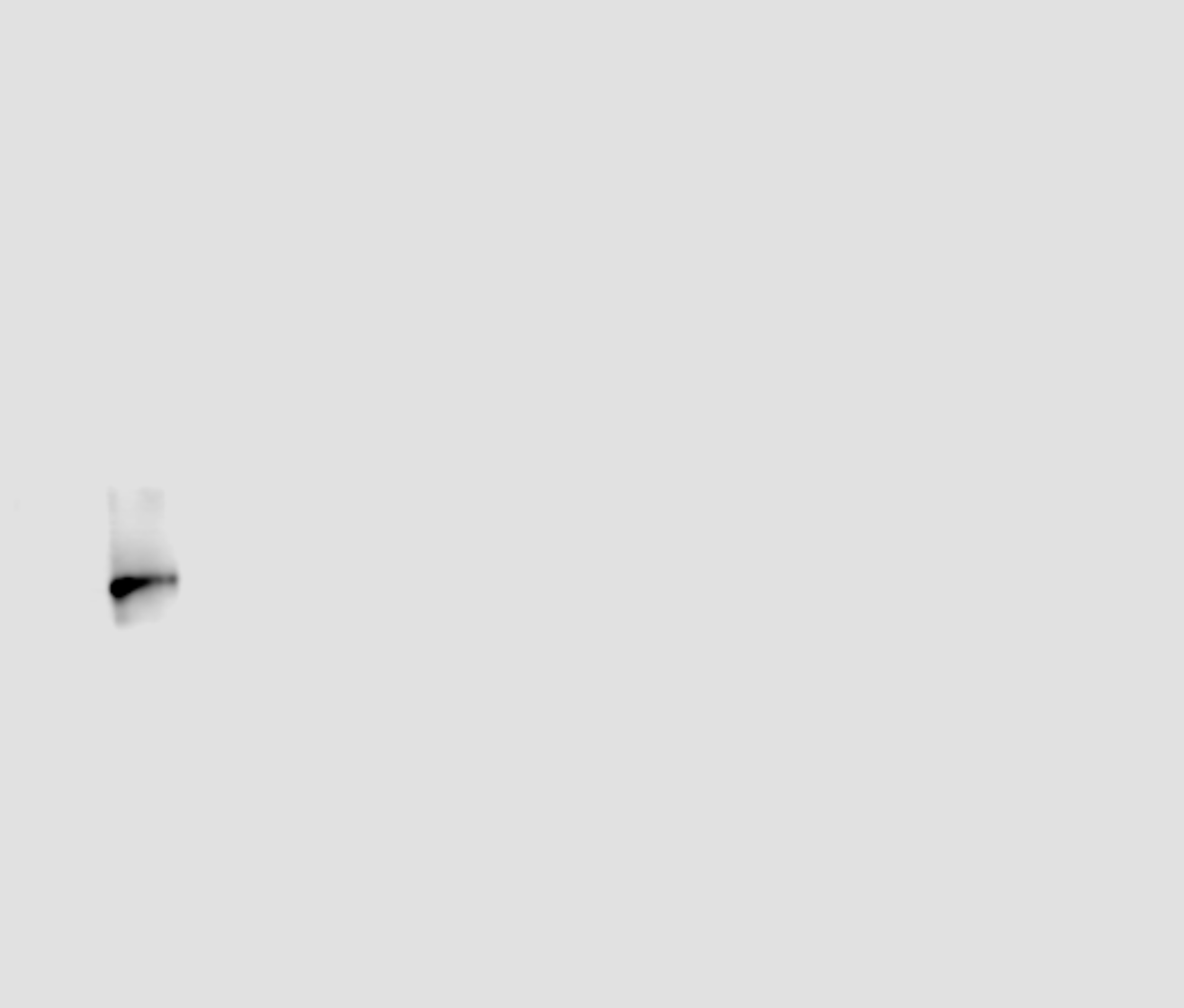

Supplement: Figure 4—source data 8. [file elife-99217-fig4-data8.zip › Figure 4Dπü«πé│πâÆπéÜπâ╝/GFP-PNKP iPOND.png]

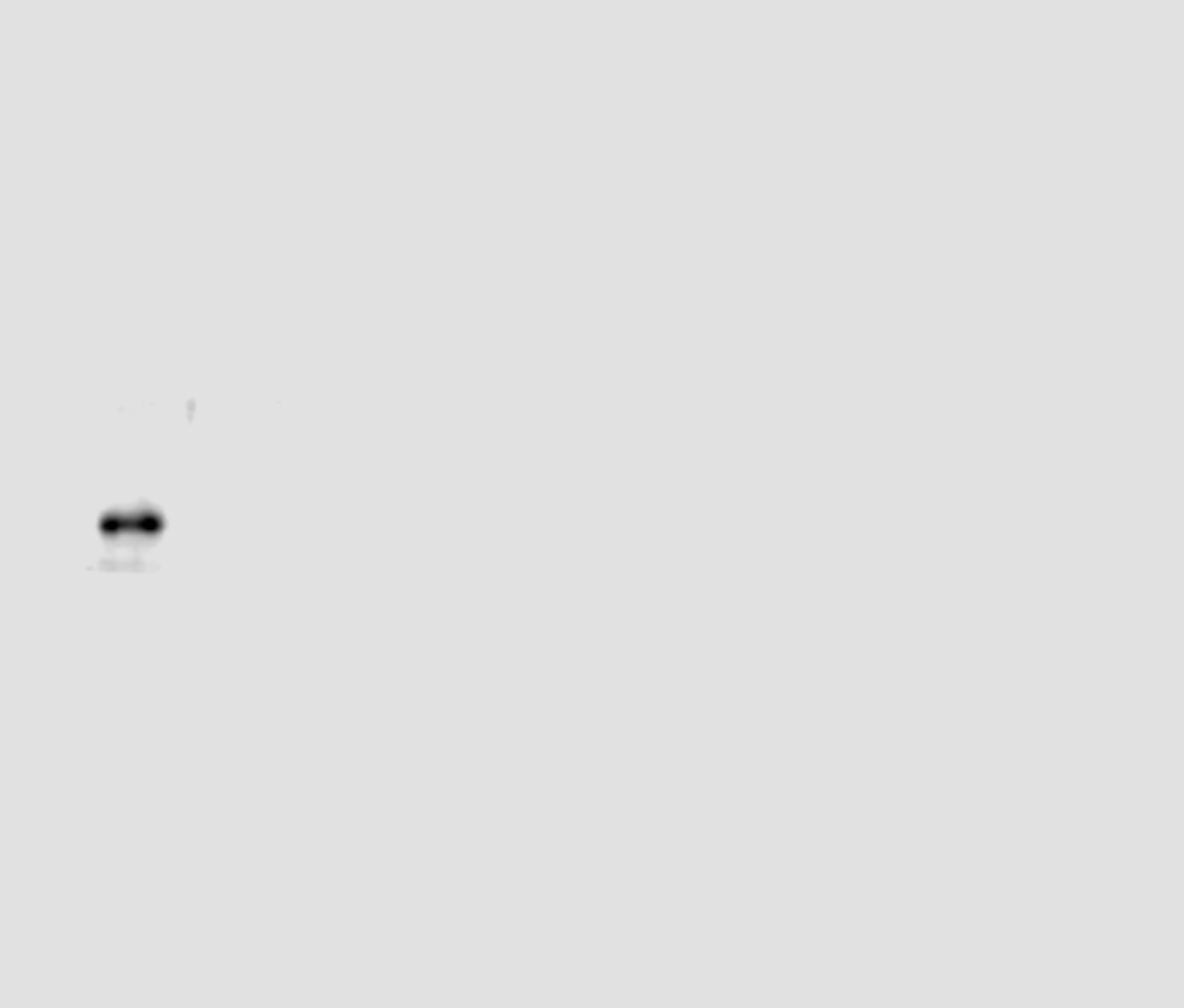

Supplement: Figure 4—source data 8. [file elife-99217-fig4-data8.zip › Figure 4Dπü«πé│πâÆπéÜπâ╝/RPA2 iPOND.png]

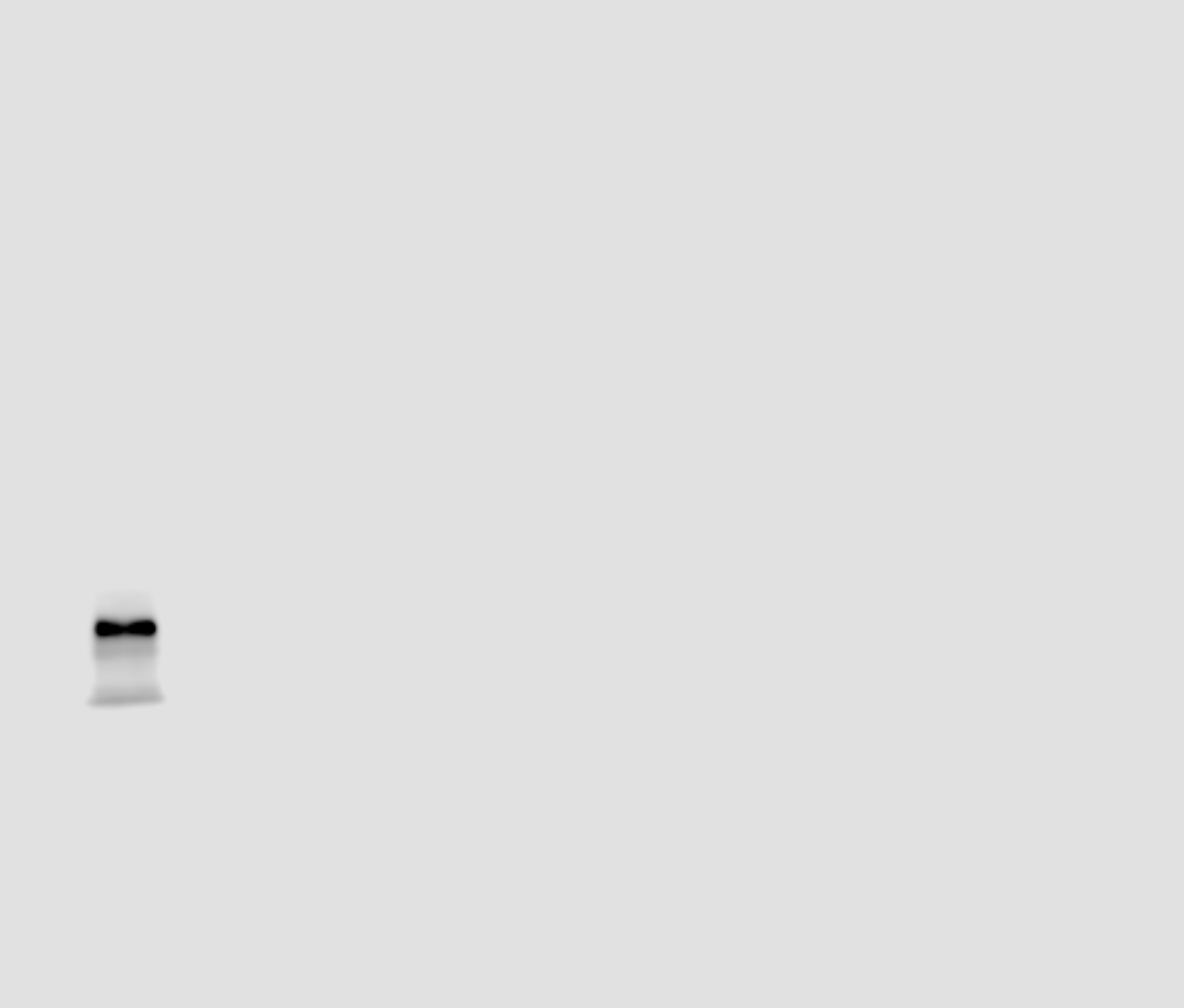

Supplement: Figure 4—source data 8. [file elife-99217-fig4-data8.zip › Figure 4Dπü«πé│πâÆπéÜπâ╝/PCNA iPOND.png]

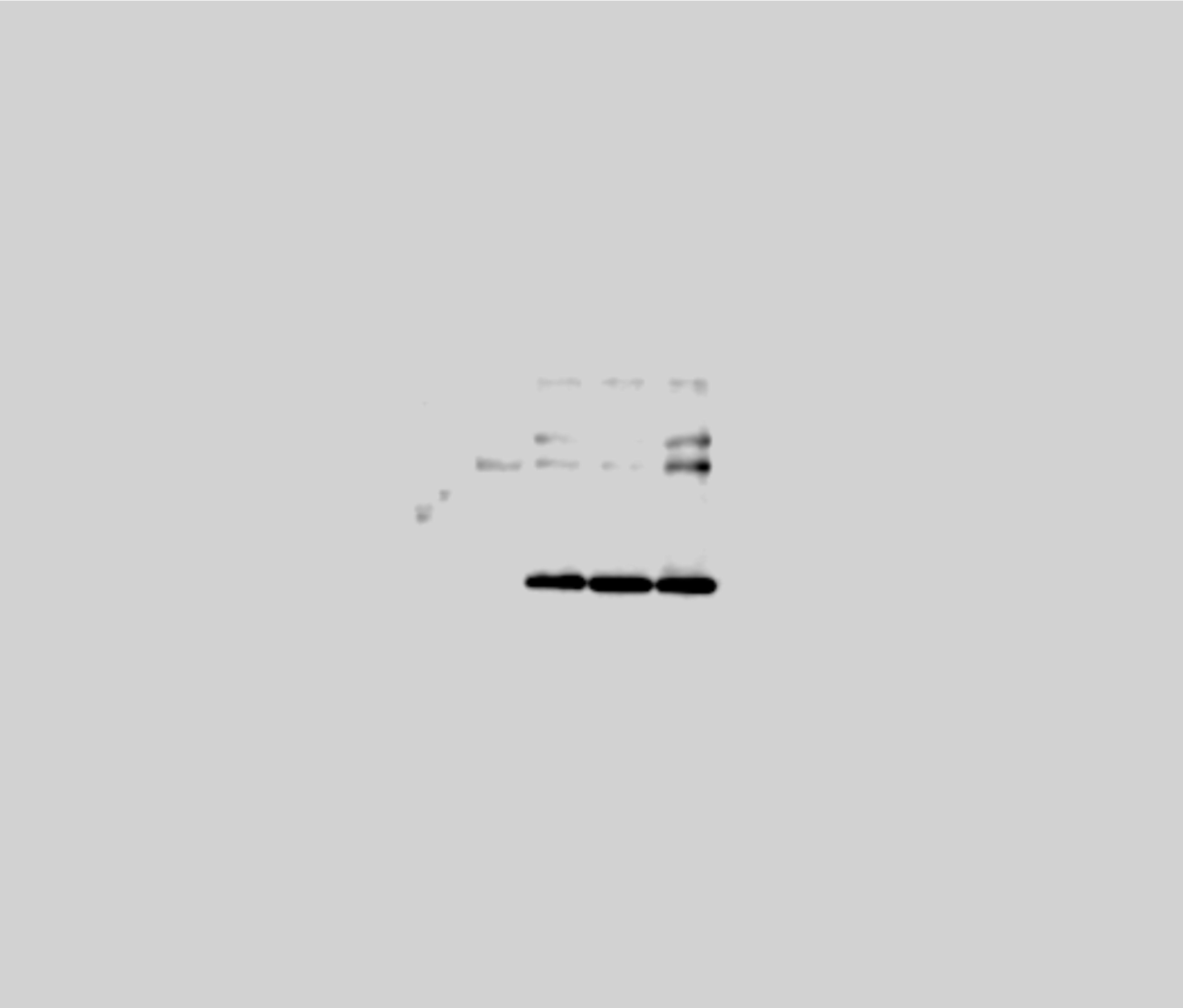

Supplement: Figure 4—source data 10. [file elife-99217-fig4-data10.zip › Figure 4E, Source Data2/Input GFP-PNKP.tif]

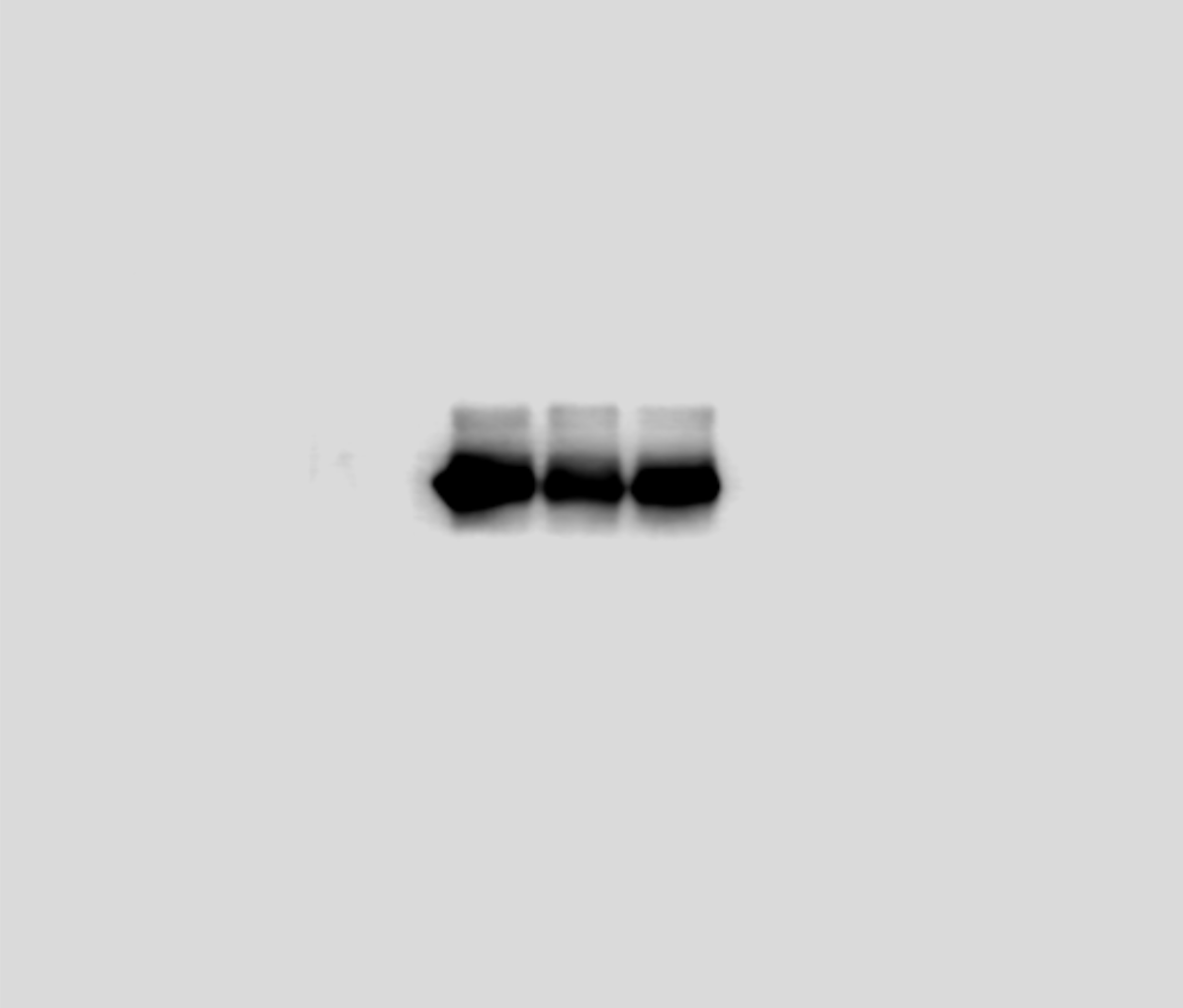

Supplement: Figure 4—source data 10. [file elife-99217-fig4-data10.zip › Figure 4E, Source Data2/Input PCNA.tif]

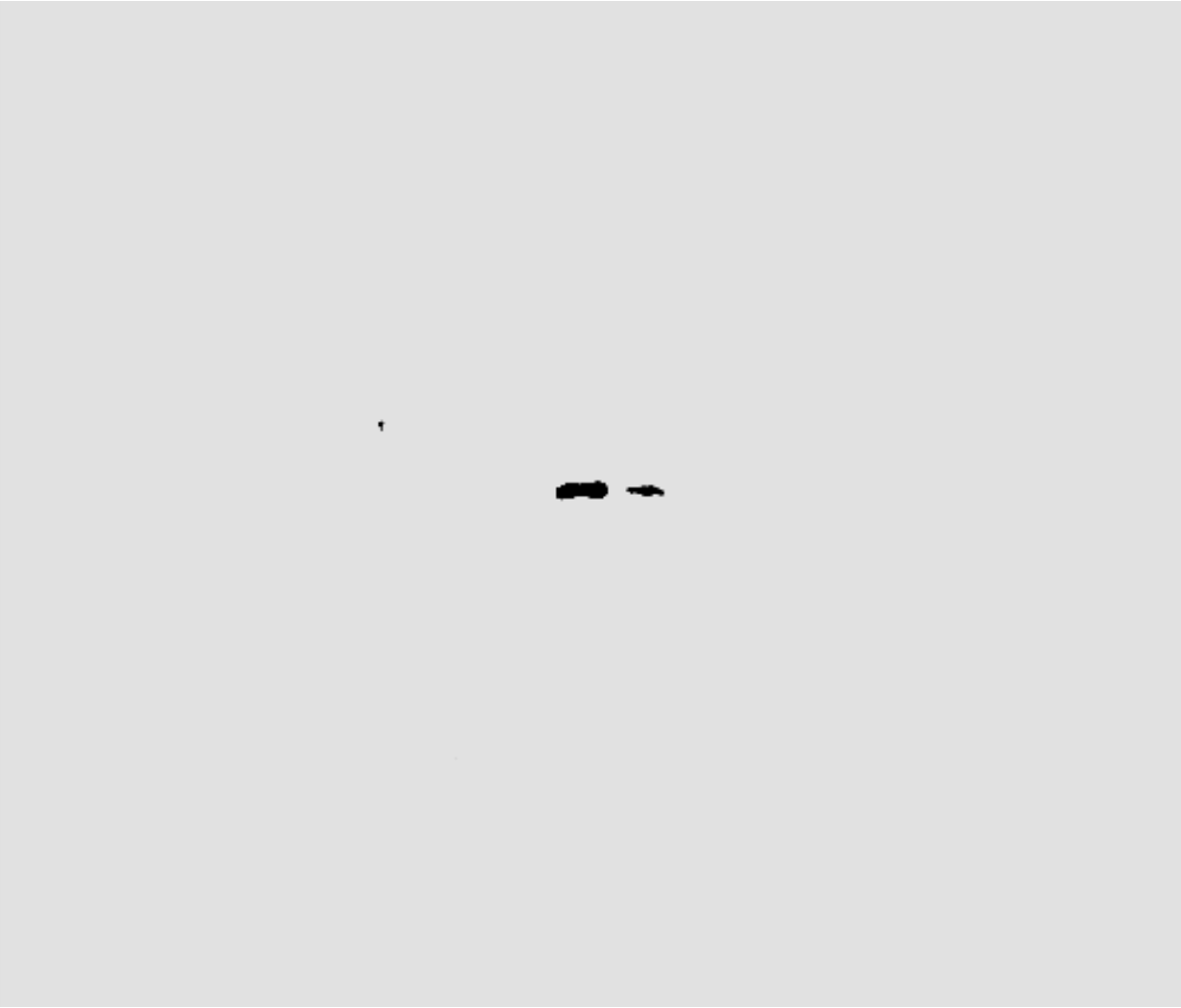

Supplement: Figure 4—source data 10. [file elife-99217-fig4-data10.zip › Figure 4E, Source Data2/iPOND GFP-PNKP.tif]

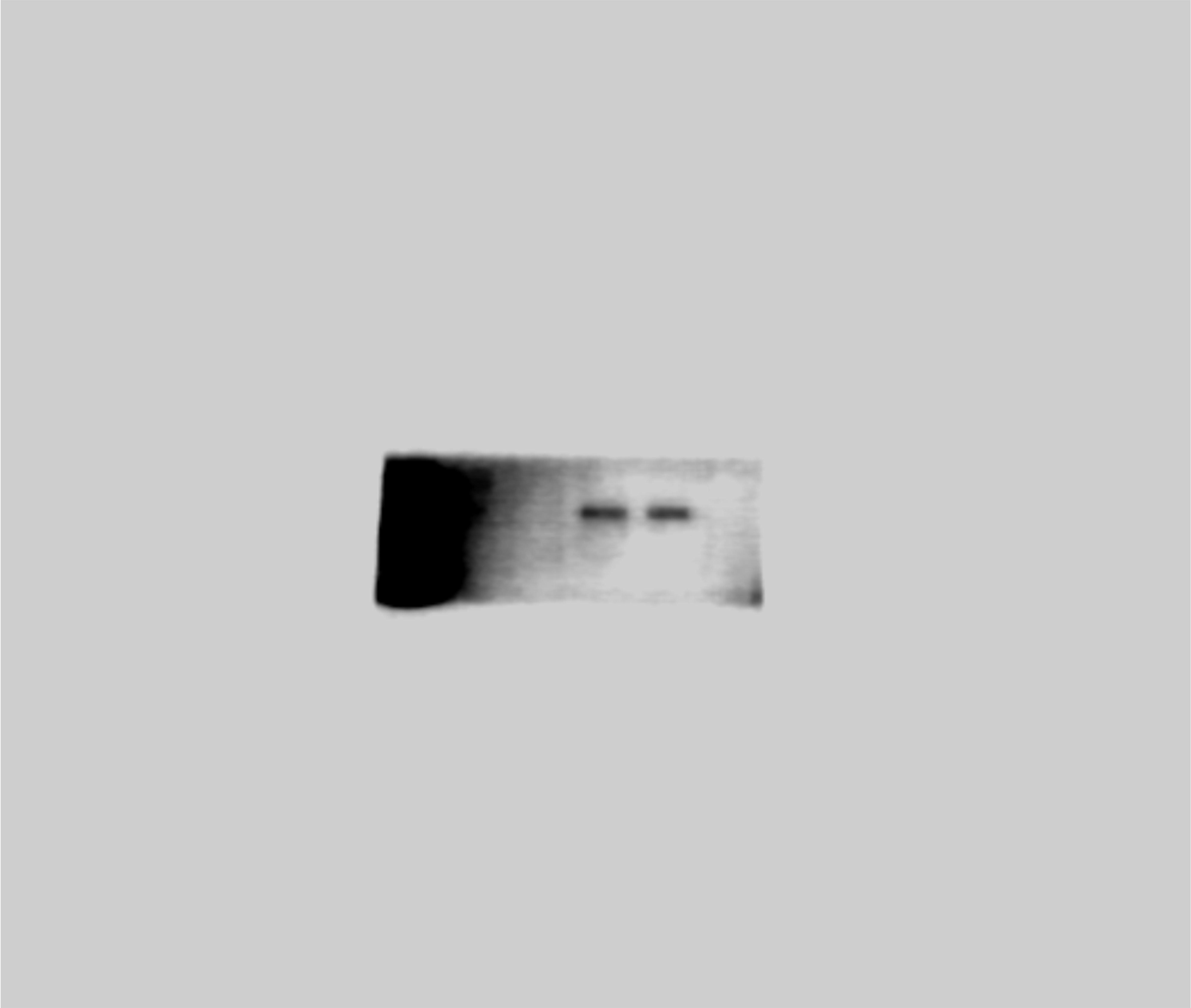

Supplement: Figure 4—source data 10. [file elife-99217-fig4-data10.zip › Figure 4E, Source Data2/iPOND PCNA.tif]

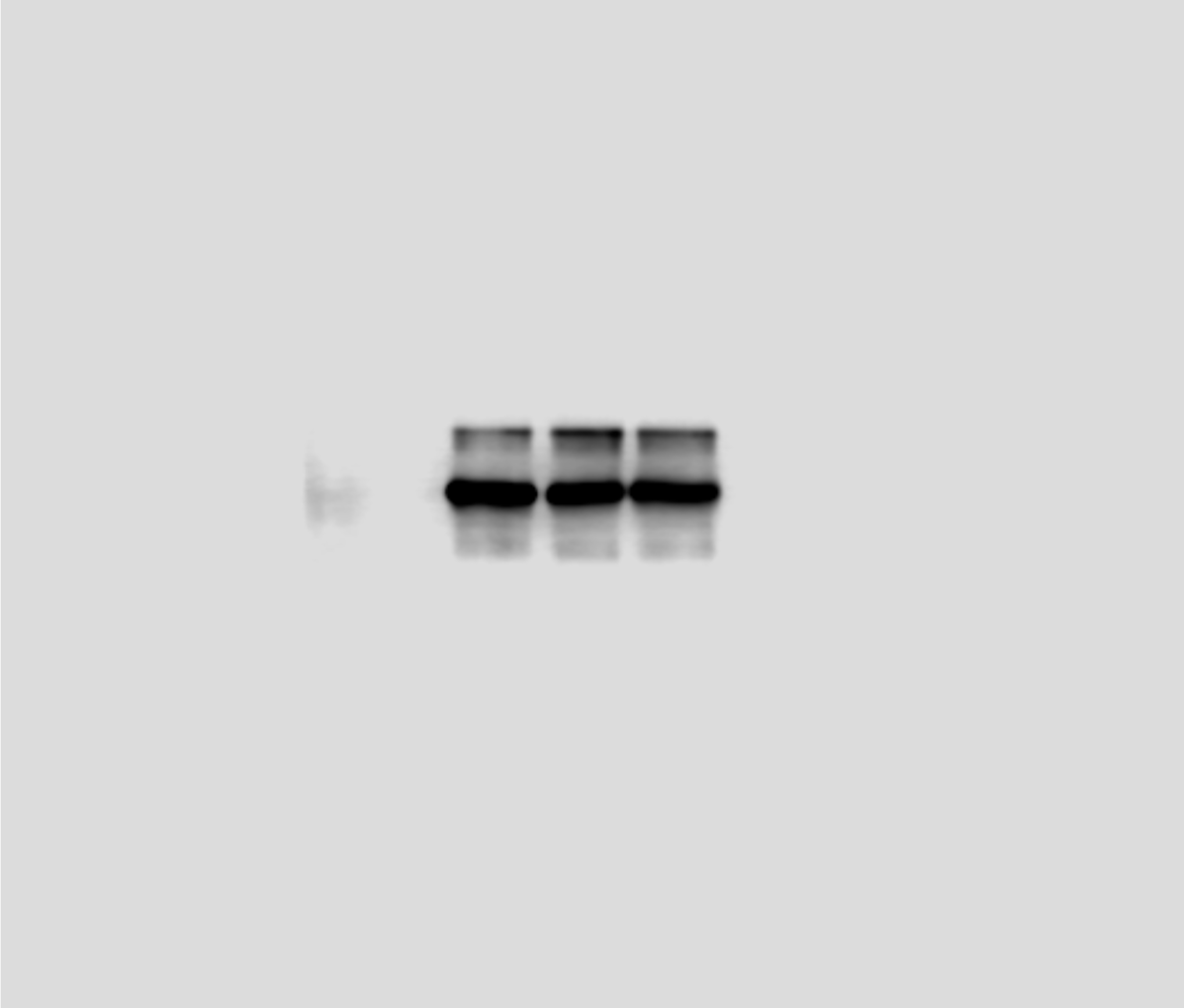

Supplement: Figure 4—source data 12. [file elife-99217-fig4-data12.zip › Figure 4F, Source Data2/Input PCNA.tif]

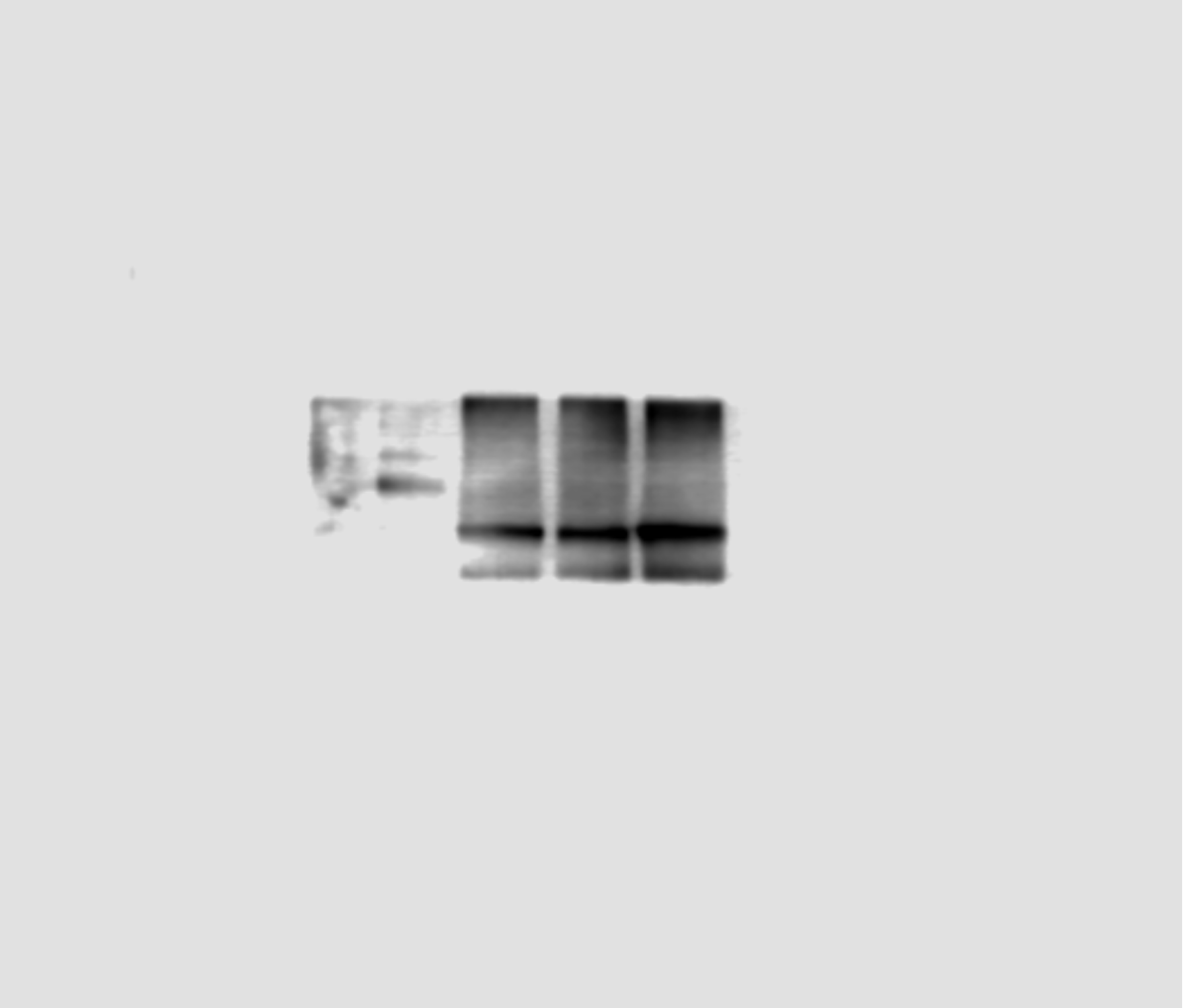

Supplement: Figure 4—source data 12. [file elife-99217-fig4-data12.zip › Figure 4F, Source Data2/Input PNKP.tif]

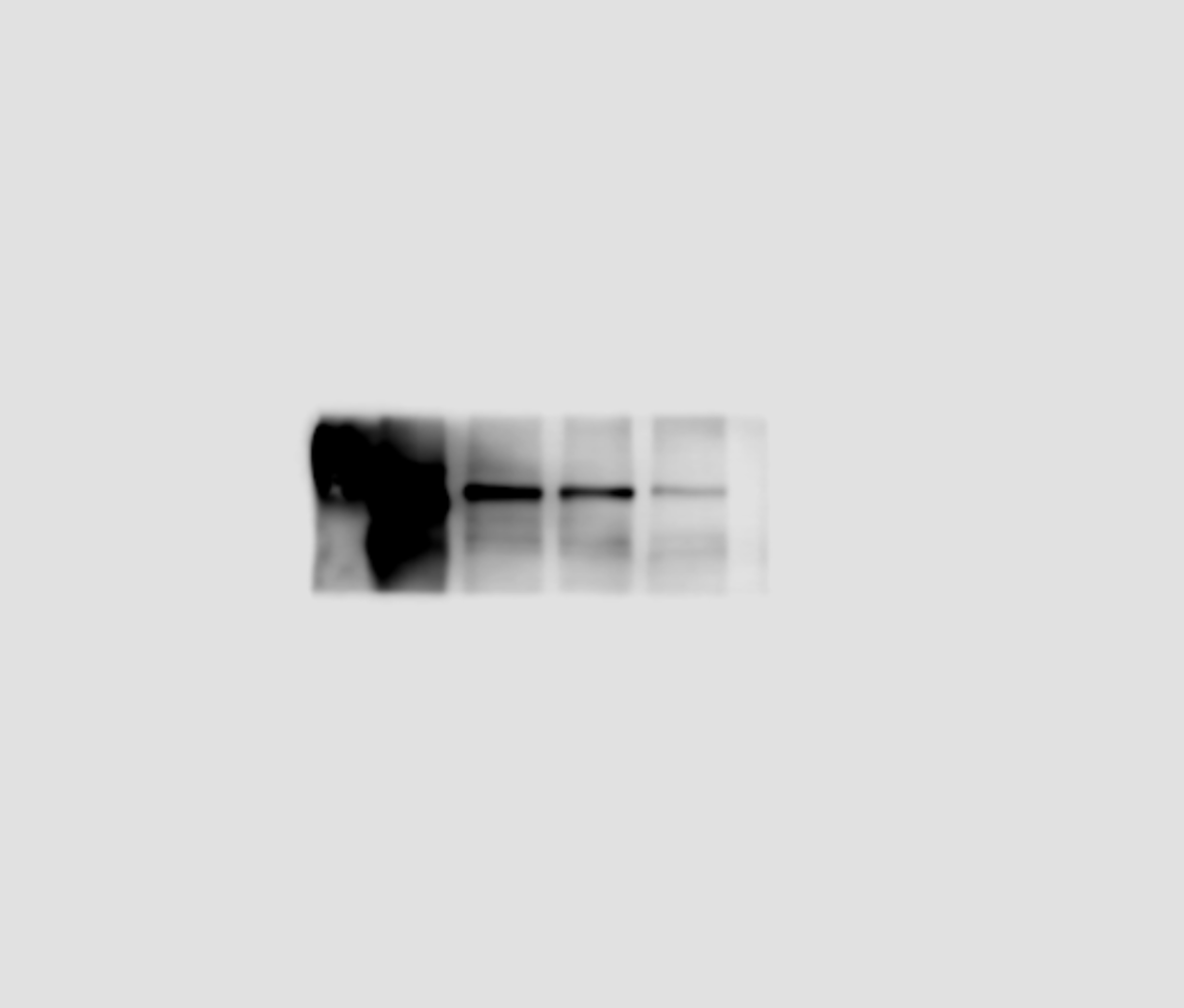

Supplement: Figure 4—source data 12. [file elife-99217-fig4-data12.zip › Figure 4F, Source Data2/Input XRCC1.tif]

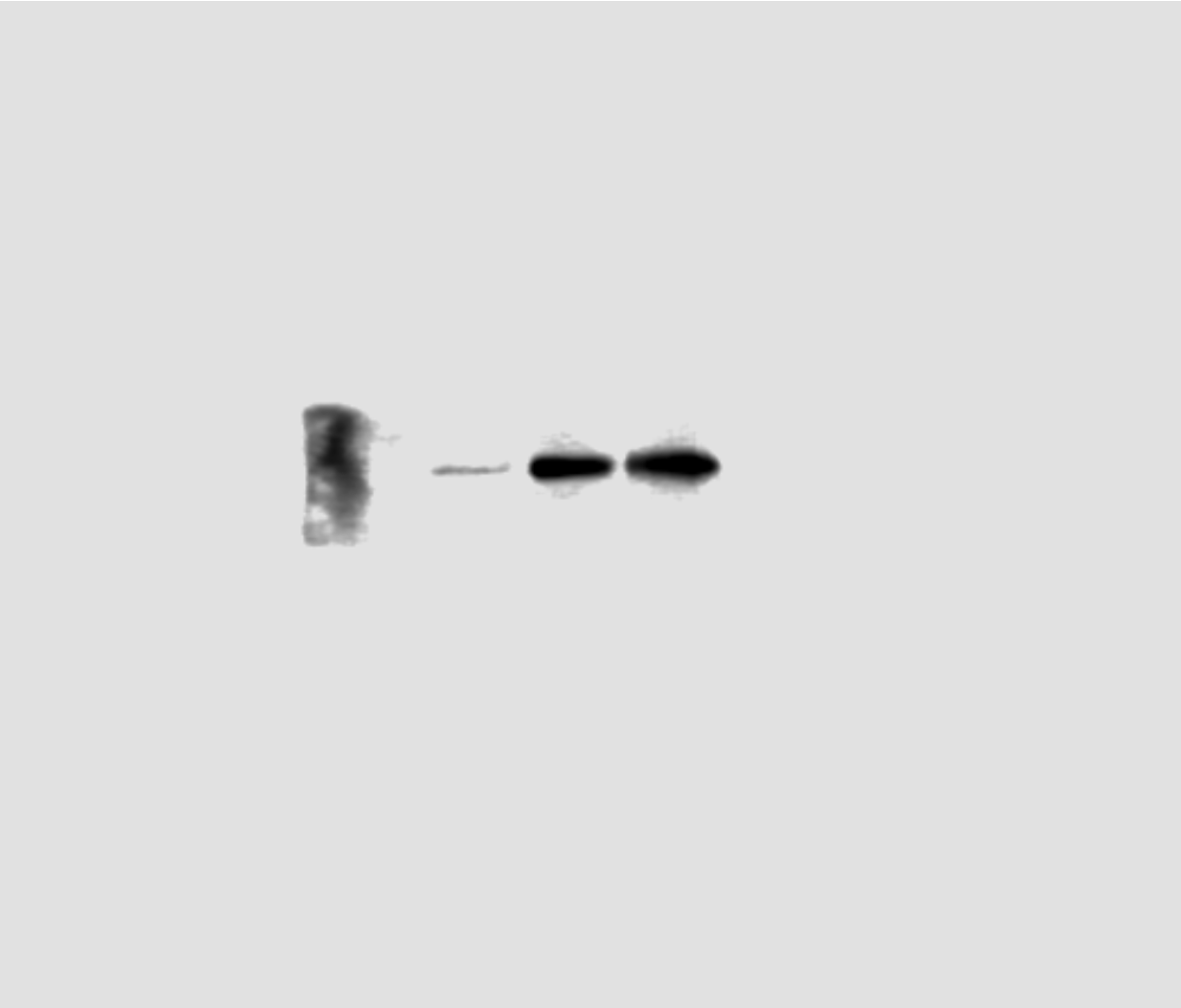

Supplement: Figure 4—source data 12. [file elife-99217-fig4-data12.zip › Figure 4F, Source Data2/iPOND PCNA.tif]

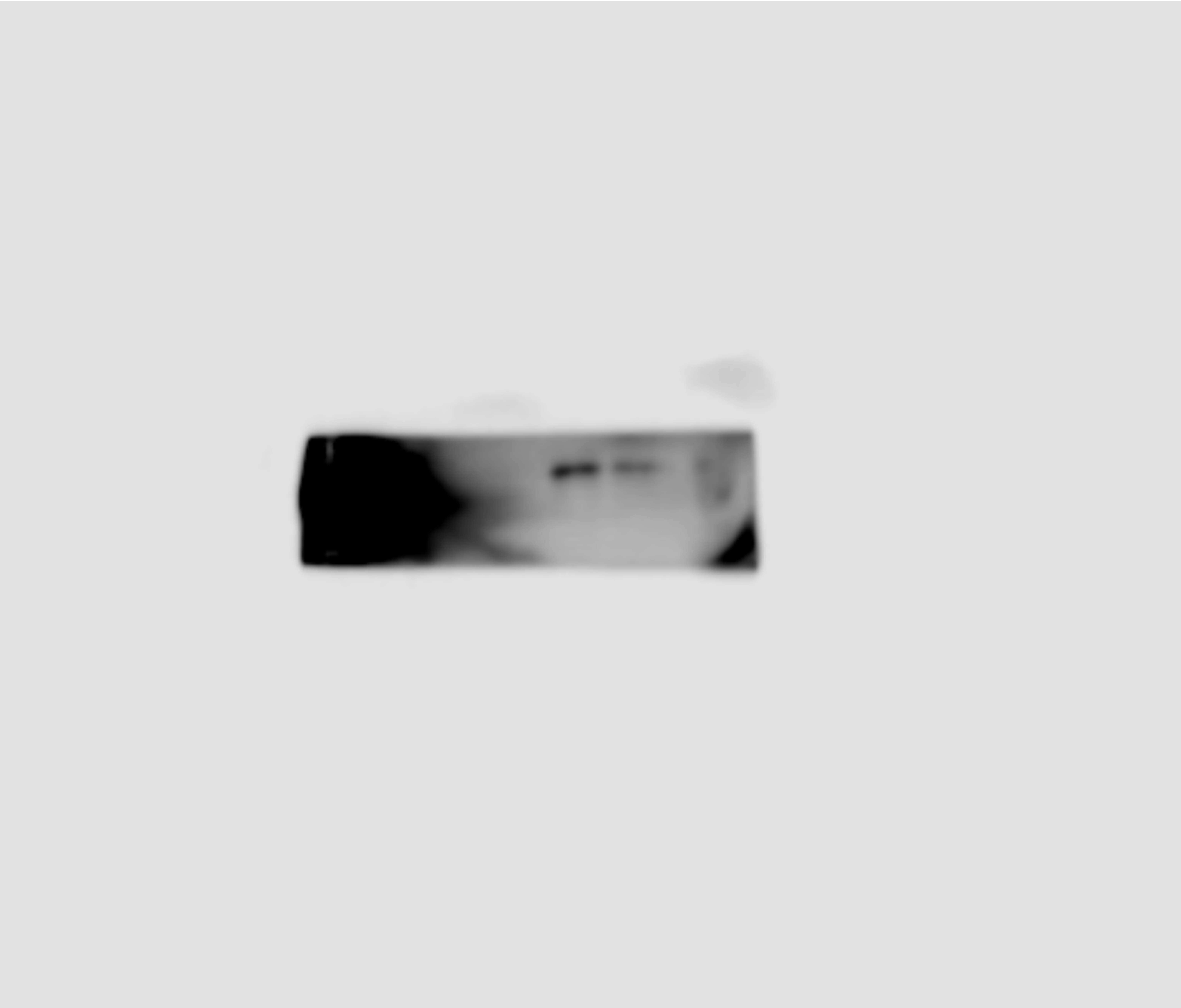

Supplement: Figure 4—source data 12. [file elife-99217-fig4-data12.zip › Figure 4F, Source Data2/iPOND PNKP.tif]

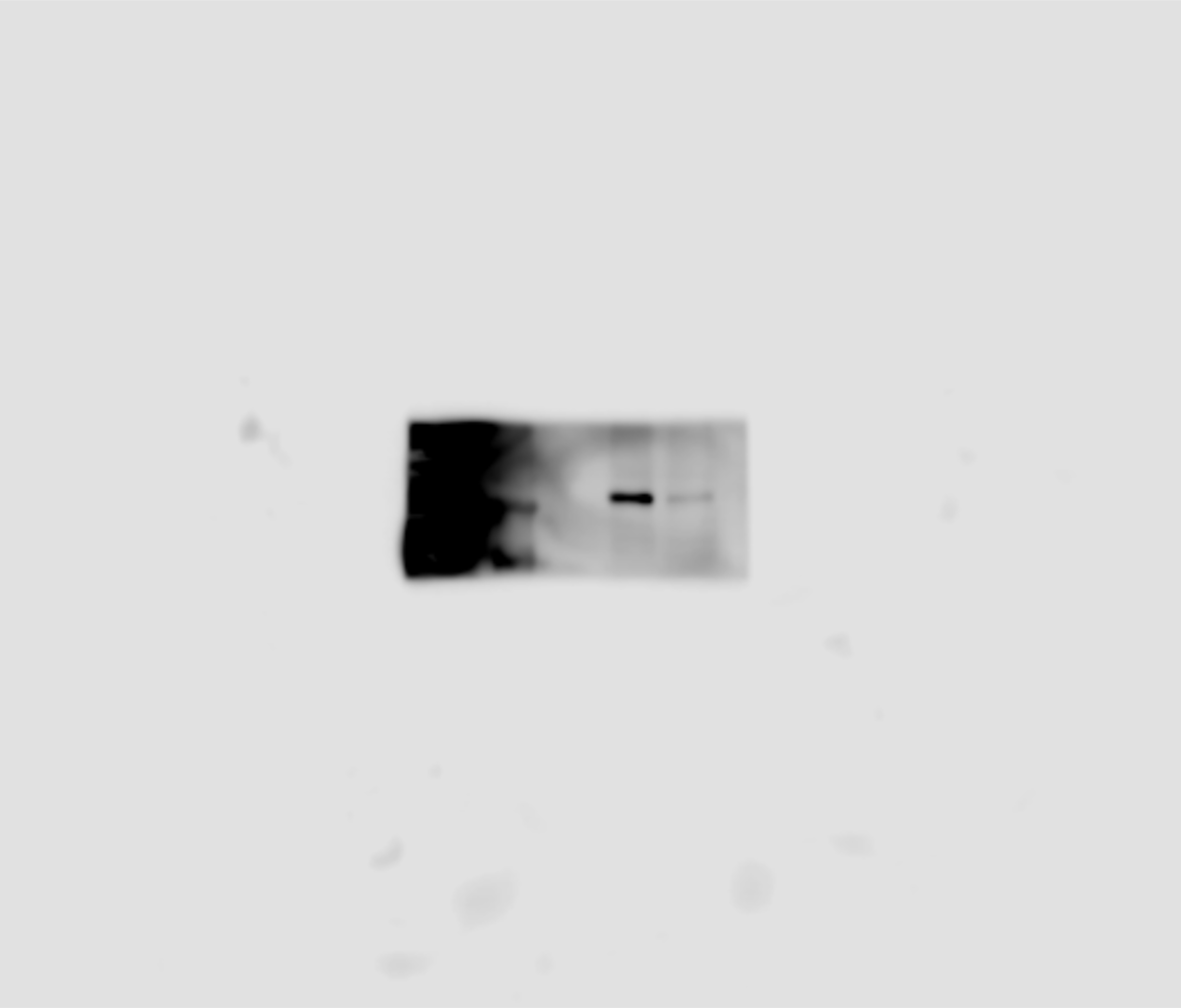

Supplement: Figure 4—source data 12. [file elife-99217-fig4-data12.zip › Figure 4F, Source Data2/iPOND XRCC1.tif]

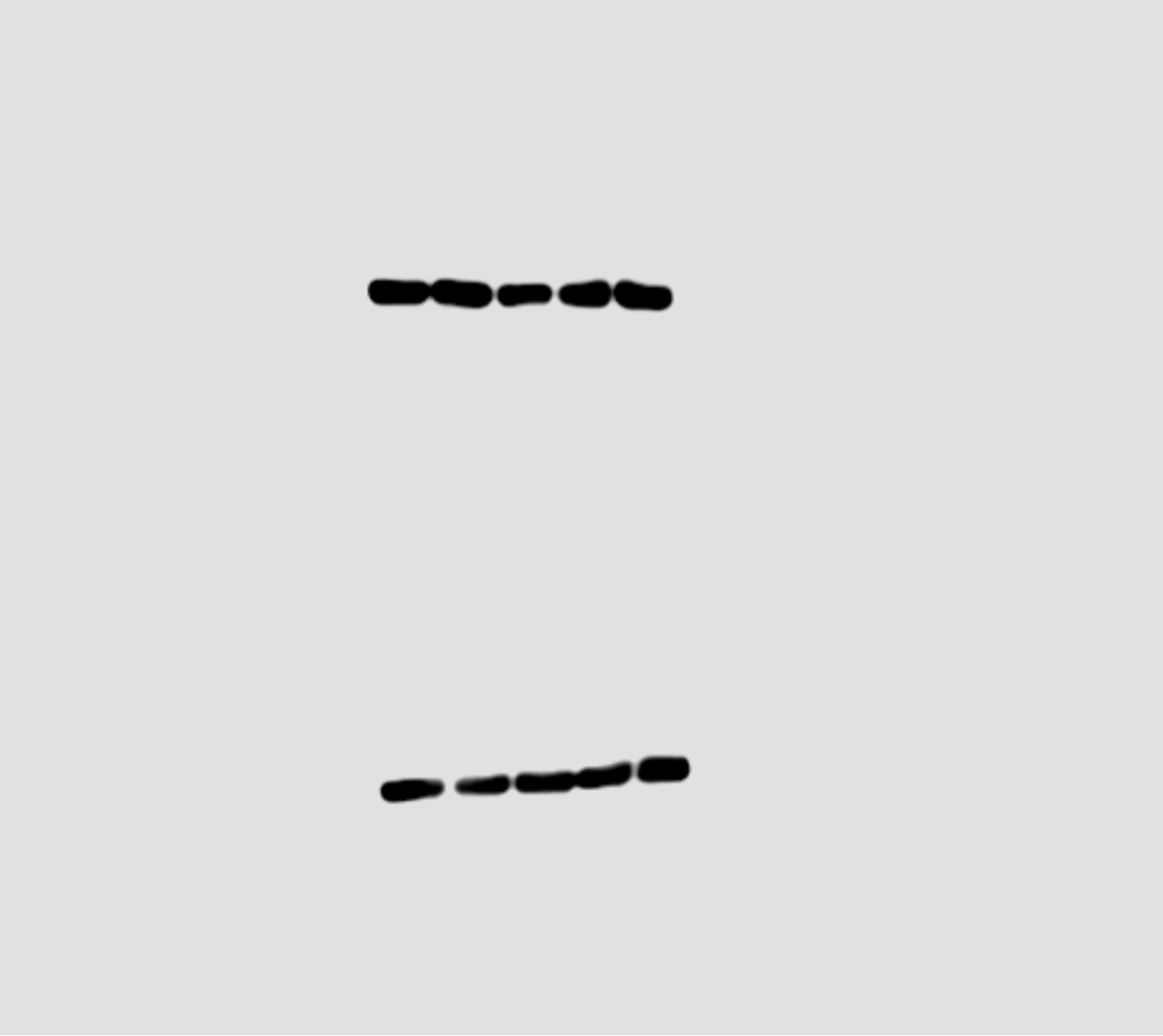

Supplement: Figure 4—figure supplement 1—source data 2. [file elife-99217-fig4-figsupp1-data2.zip › Figure 4-figure supplement Cπü«πé│πâÆπéÜπâ╝/GAPGH.png]

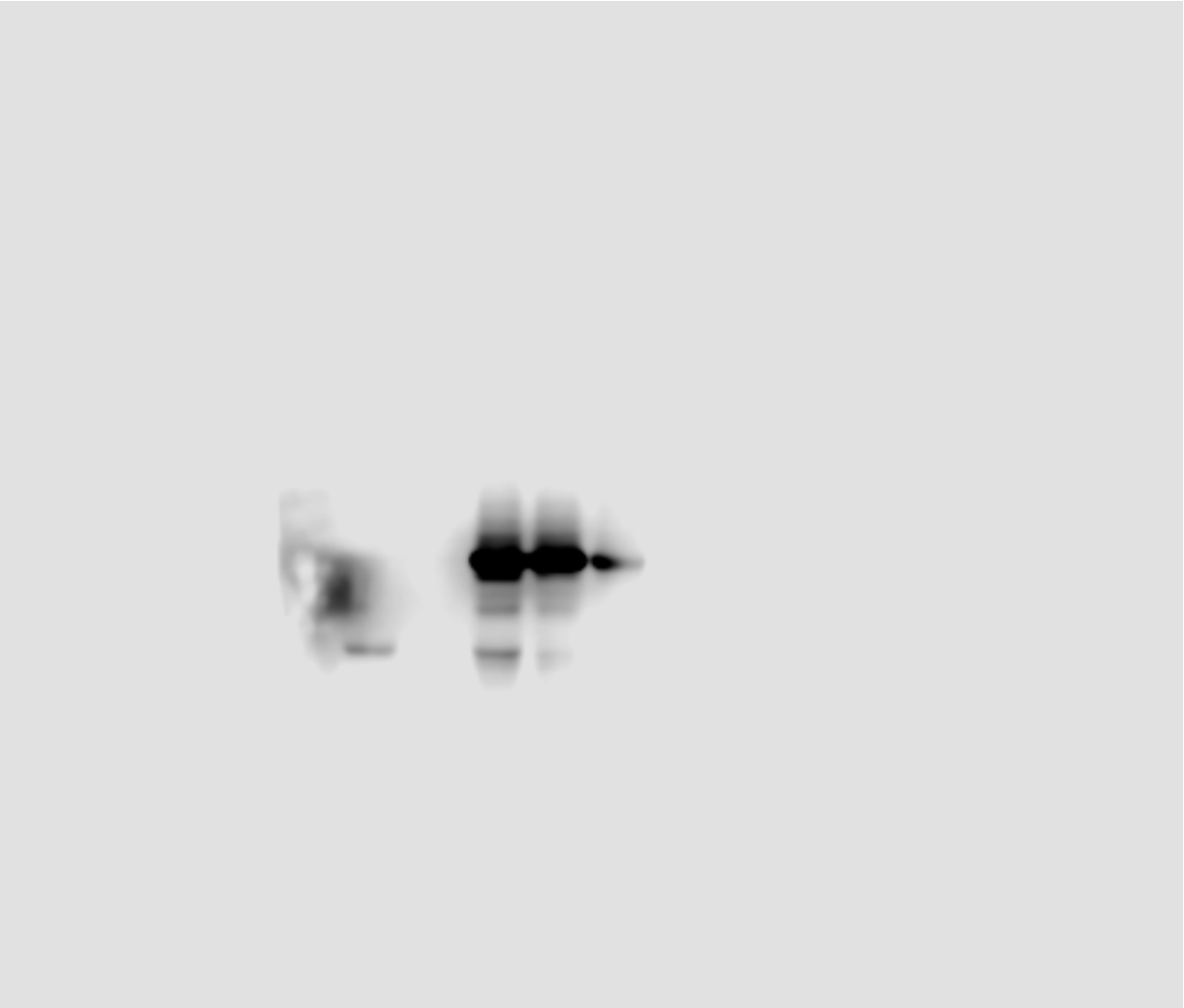

Supplement: Figure 4—figure supplement 1—source data 2. [file elife-99217-fig4-figsupp1-data2.zip › Figure 4-figure supplement Cπü«πé│πâÆπéÜπâ╝/PNKP.png]

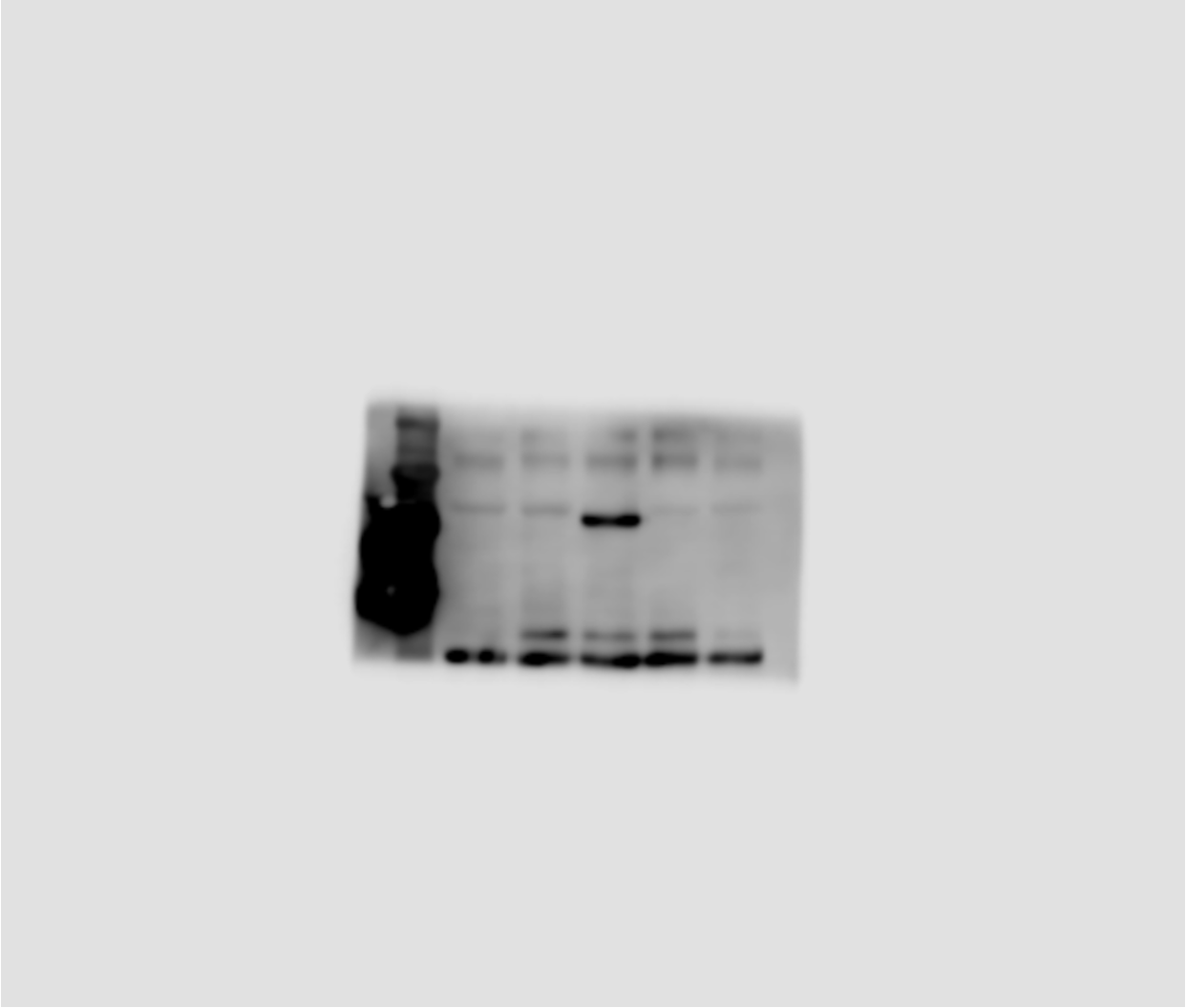

Supplement: Figure 4—figure supplement 1—source data 2. [file elife-99217-fig4-figsupp1-data2.zip › Figure 4-figure supplement Cπü«πé│πâÆπéÜπâ╝/pT118 PNKP.png]

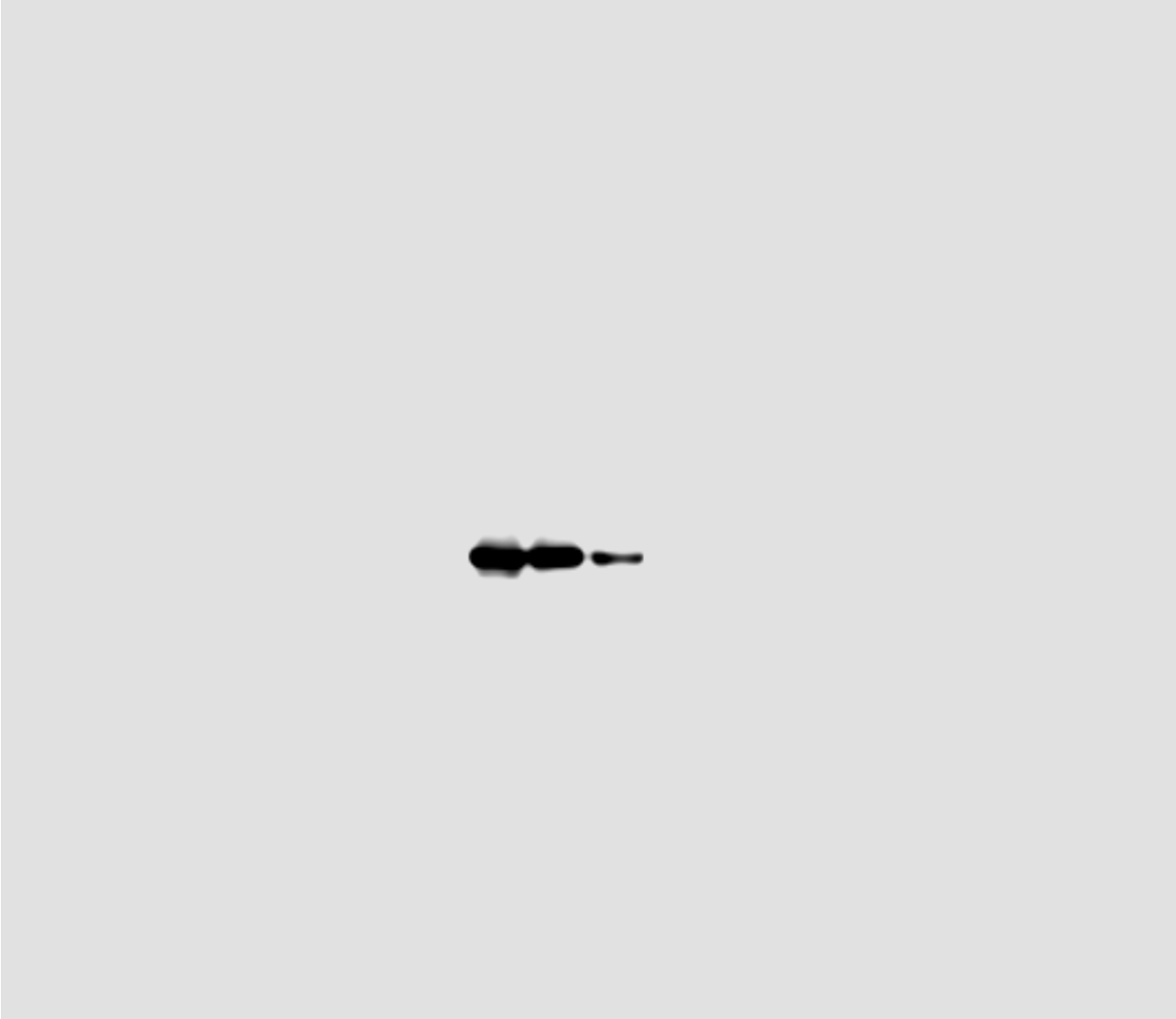

Supplement: Figure 4—figure supplement 1—source data 2. [file elife-99217-fig4-figsupp1-data2.zip › Figure 4-figure supplement Cπü«πé│πâÆπéÜπâ╝/GFP.png]

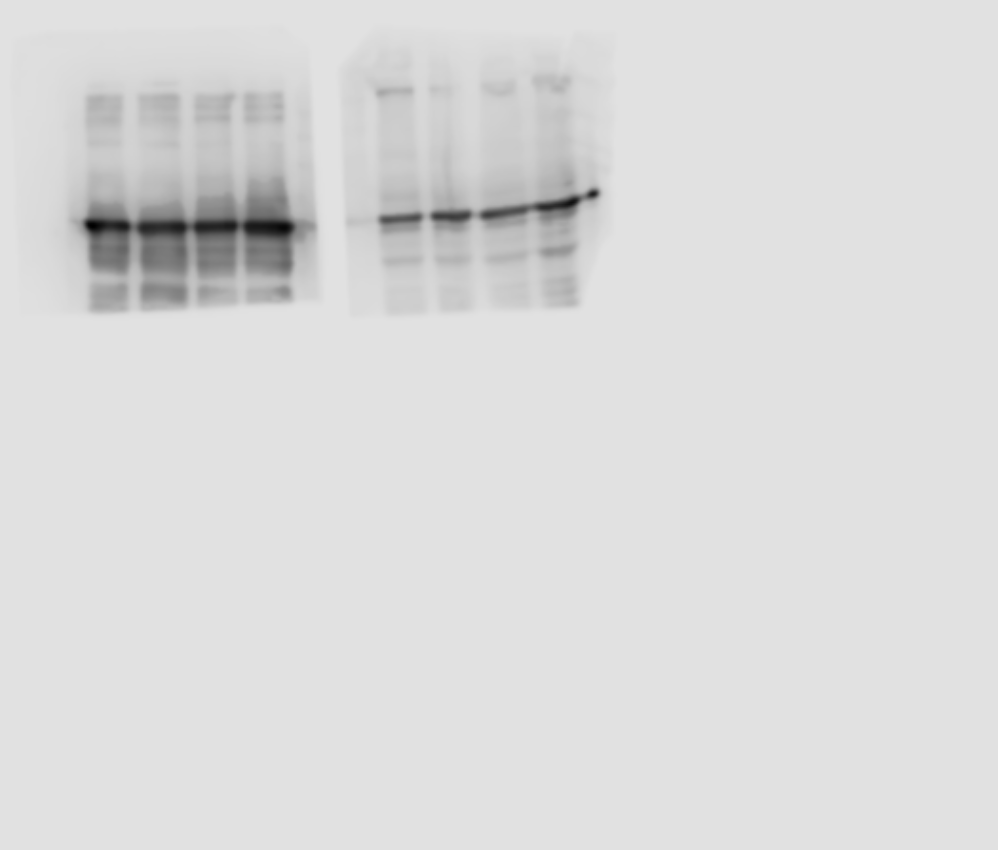

Supplement: Figure 4—figure supplement 1—source data 4. [file elife-99217-fig4-figsupp1-data4.zip › GFP.tif]

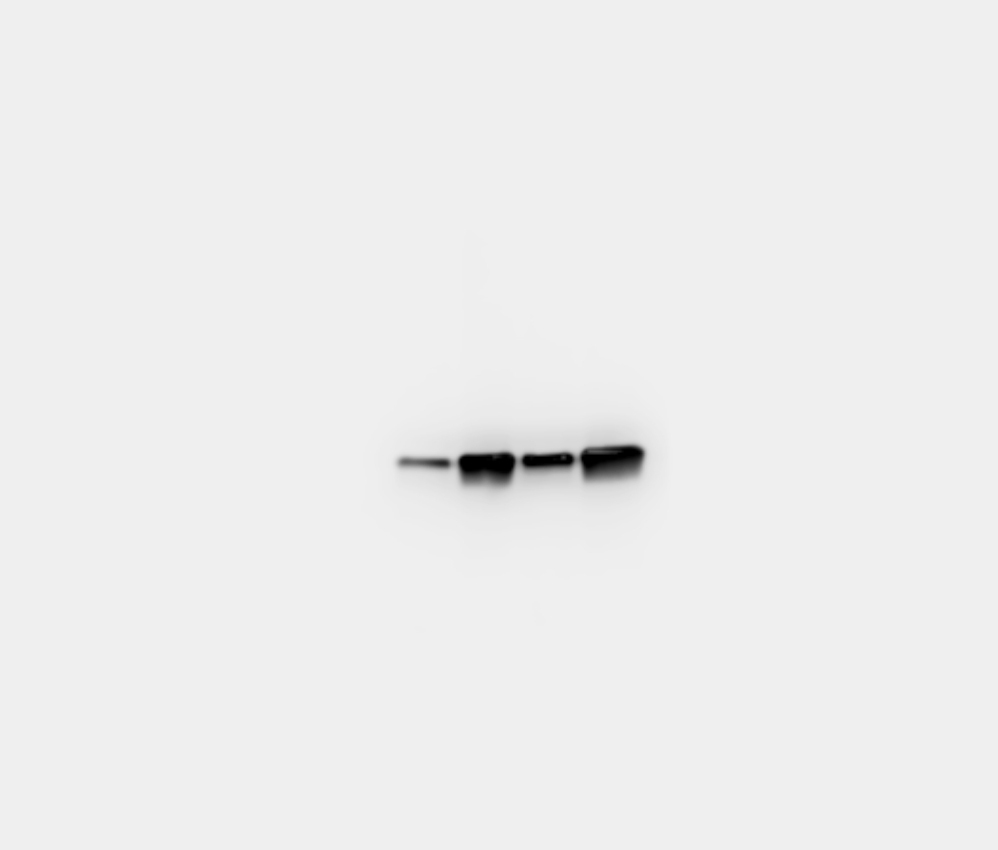

Supplement: Figure 4—figure supplement 1—source data 4. [file elife-99217-fig4-figsupp1-data4.zip › input FLAG-CDK1_2_FLAG antibody.jpg]

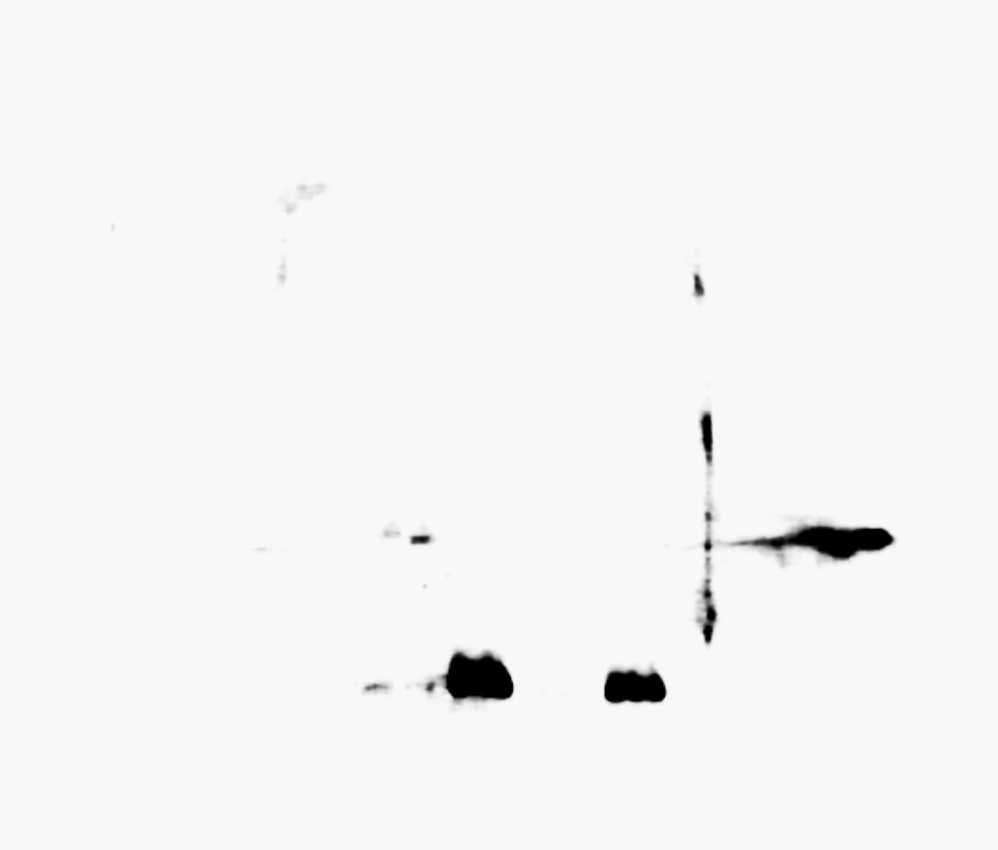

Supplement: Figure 4—figure supplement 1—source data 4. [file elife-99217-fig4-figsupp1-data4.zip › FLAG_IP_long exposure.jpg]

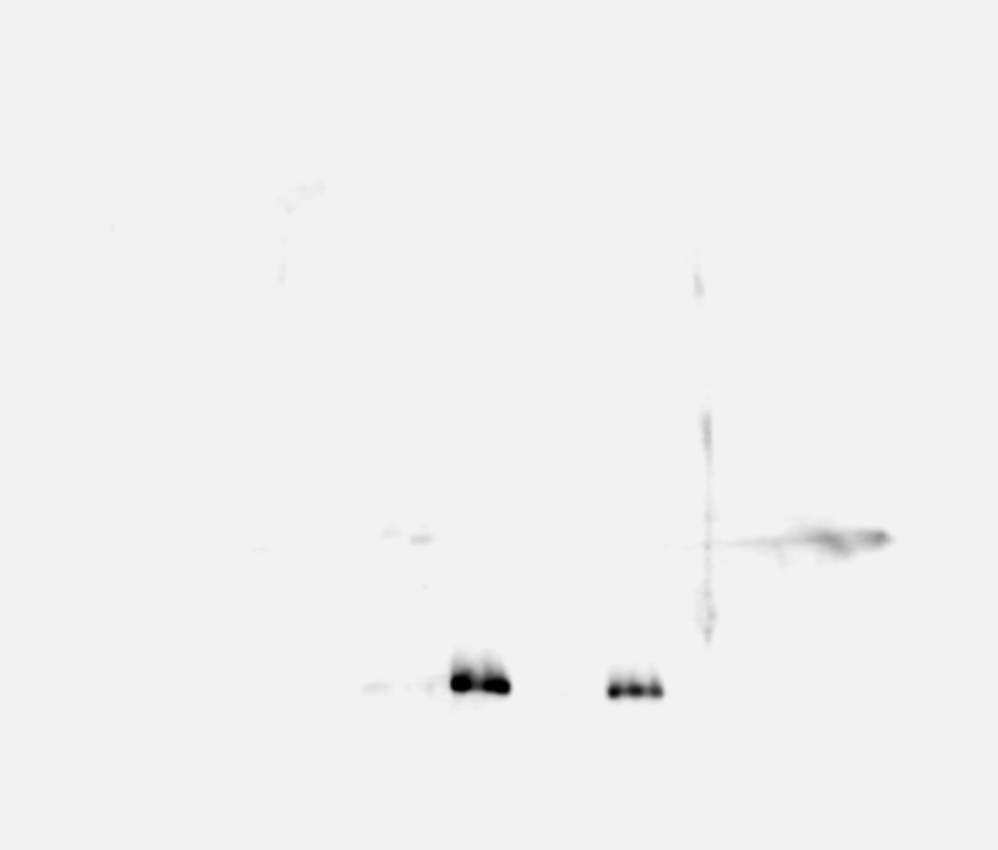

Supplement: Figure 4—figure supplement 1—source data 4. [file elife-99217-fig4-figsupp1-data4.zip › FLAG-IP_short exposure.jpg]

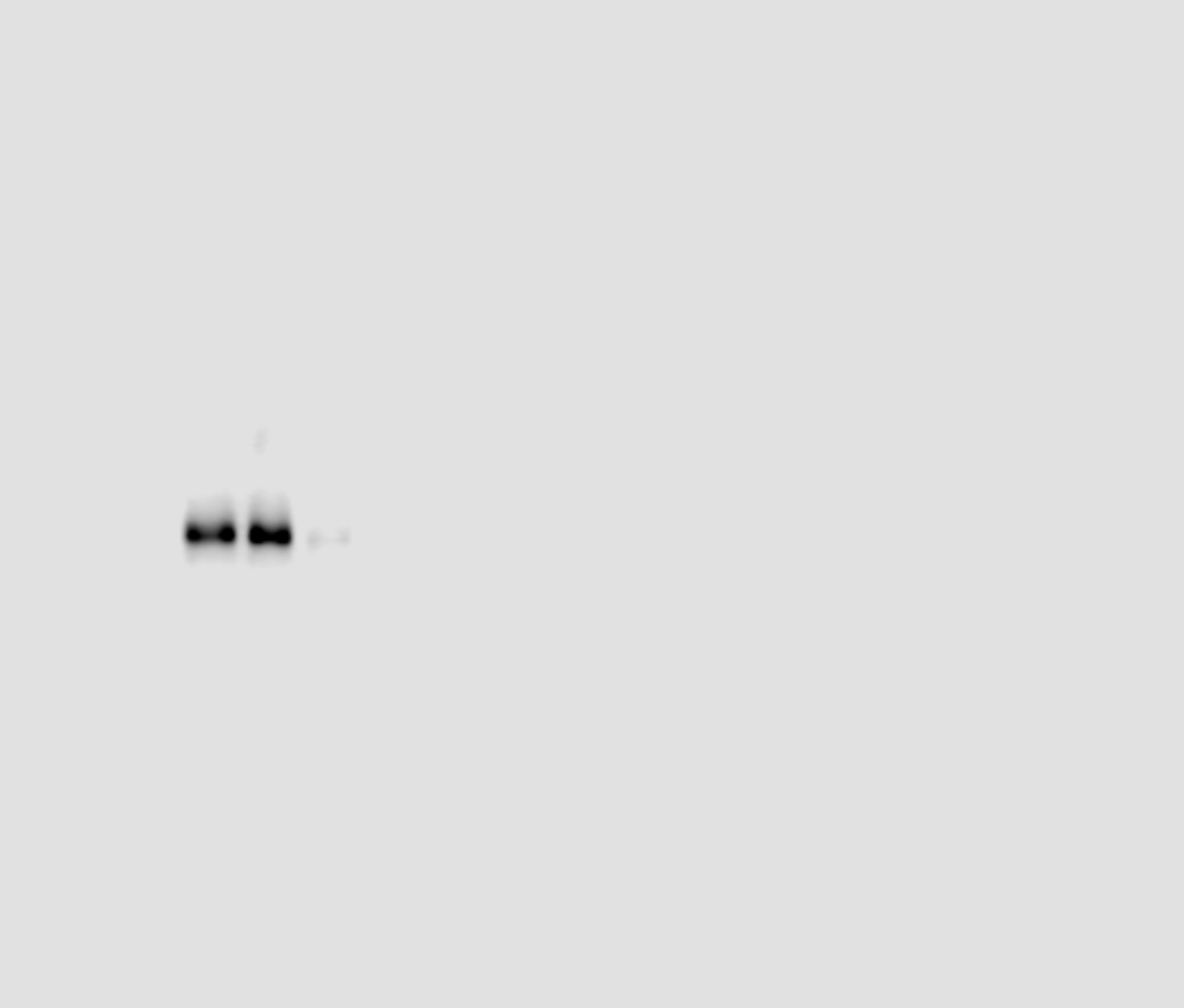

Supplement: Figure 4—figure supplement 1—source data 6. [file elife-99217-fig4-figsupp1-data6.zip › Figure 4-figure supplement Fπü«πé│πâÆπéÜπâ╝/PNKP input.tif]

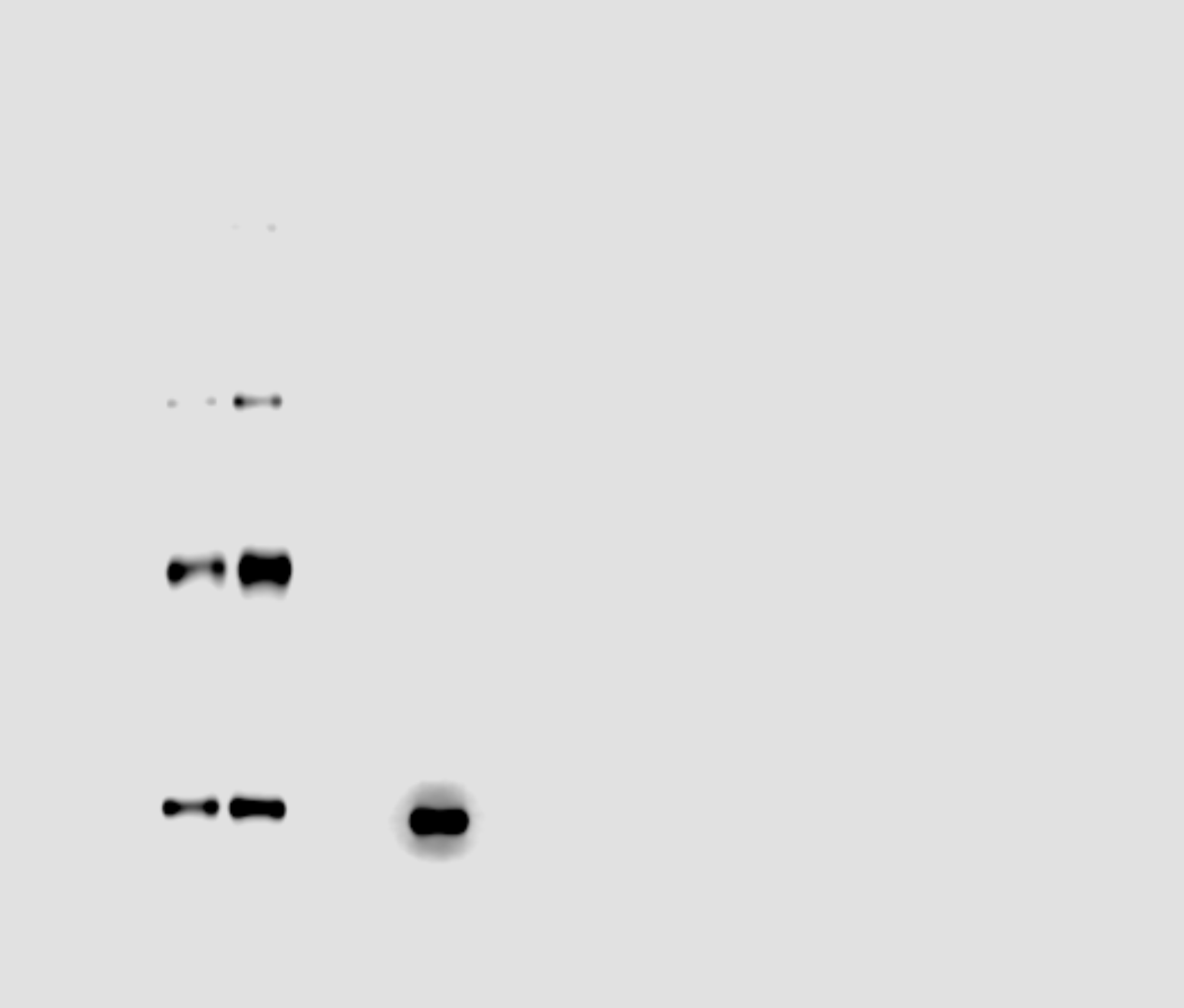

Supplement: Figure 4—figure supplement 1—source data 6. [file elife-99217-fig4-figsupp1-data6.zip › Figure 4-figure supplement Fπü«πé│πâÆπéÜπâ╝/PCNA input.png]

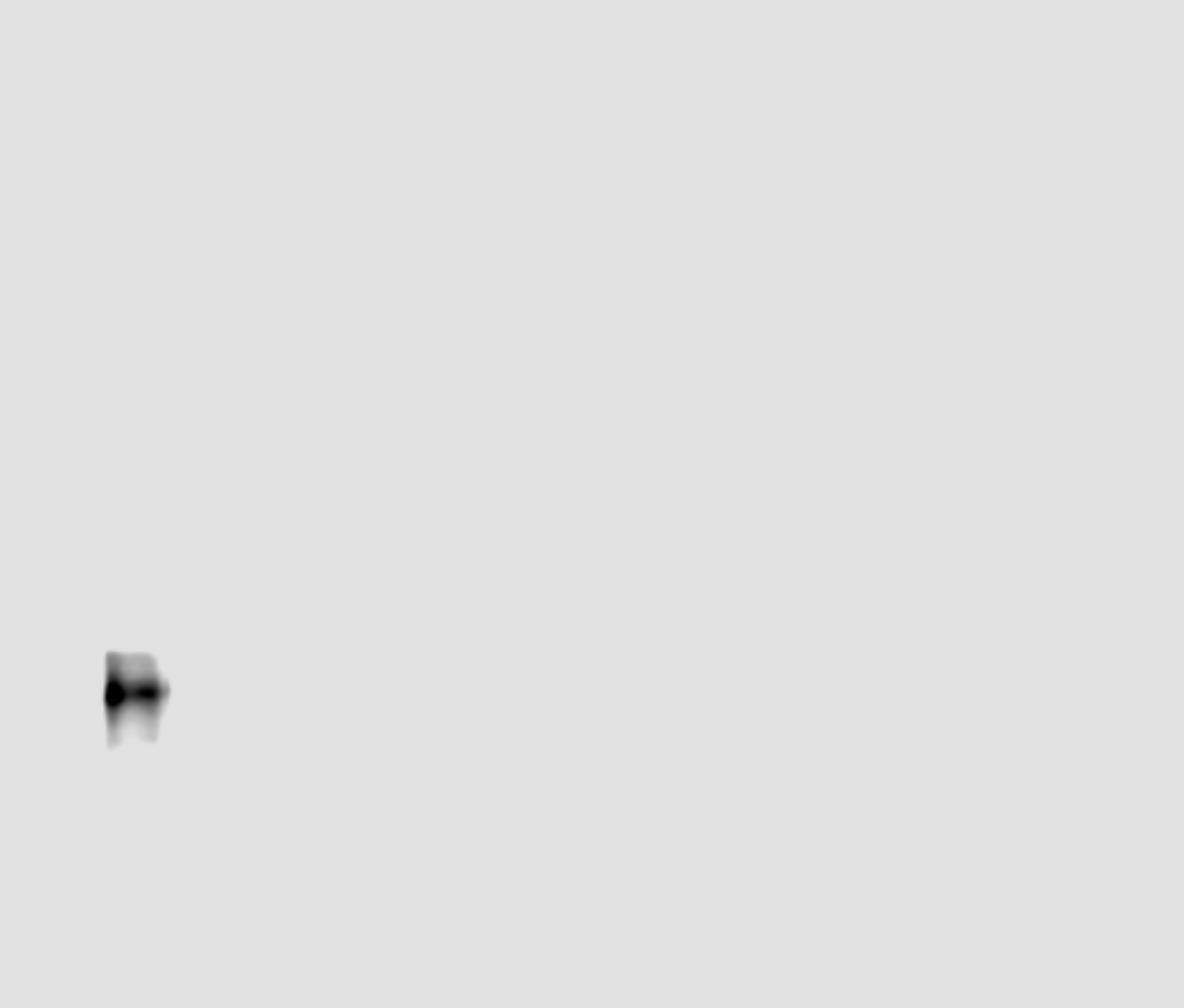

Supplement: Figure 4—figure supplement 1—source data 6. [file elife-99217-fig4-figsupp1-data6.zip › Figure 4-figure supplement Fπü«πé│πâÆπéÜπâ╝/PNKP iPOND.png]

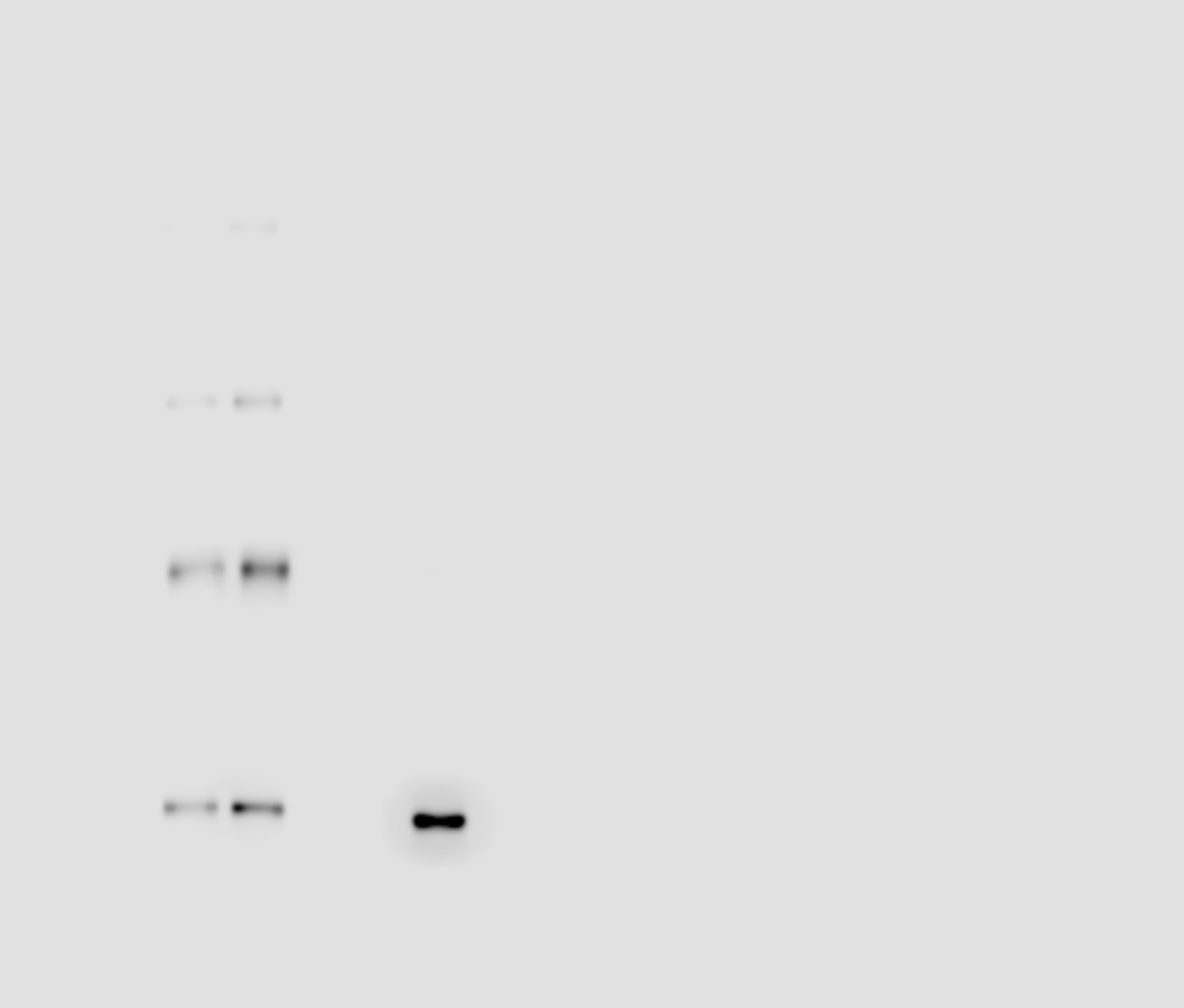

Supplement: Figure 4—figure supplement 1—source data 6. [file elife-99217-fig4-figsupp1-data6.zip › Figure 4-figure supplement Fπü«πé│πâÆπéÜπâ╝/PCNA iPOND.png]

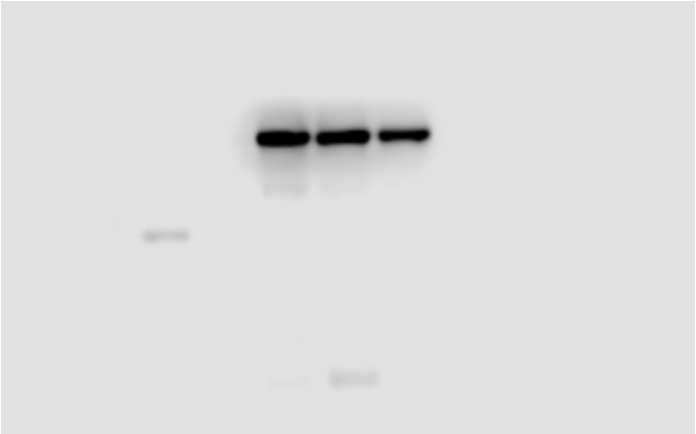

Supplement: Figure 5—figure supplement 1—source data 2. [file elife-99217-fig5-figsupp1-data2.zip › Figure 5-figure supplement Aπü«πé│πâÆπéÜπâ╝/PNKP.png]

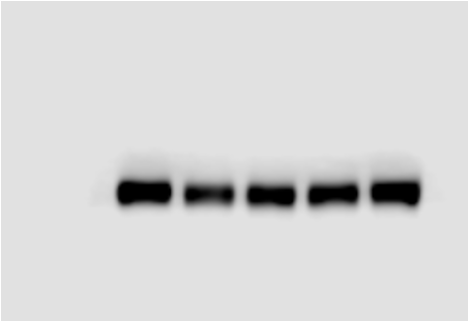

Supplement: Figure 5—figure supplement 1—source data 2. [file elife-99217-fig5-figsupp1-data2.zip › Figure 5-figure supplement Aπü«πé│πâÆπéÜπâ╝/KAP1.png]

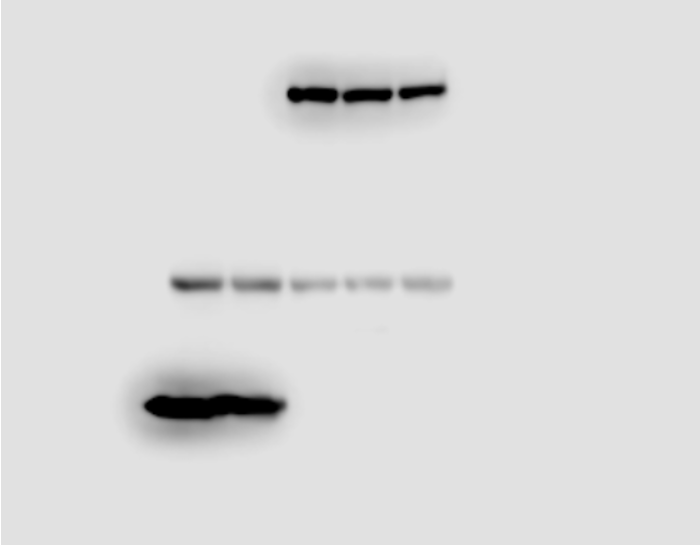

Supplement: Figure 5—figure supplement 1—source data 2. [file elife-99217-fig5-figsupp1-data2.zip › Figure 5-figure supplement Aπü«πé│πâÆπéÜπâ╝/GFP.png]

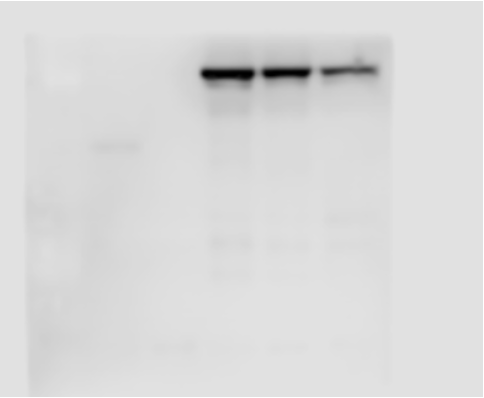

Supplement: Figure 6—figure supplement 1—source data 2. [file elife-99217-fig6-figsupp1-data2.zip › Figure 6-figure supplement Aπü«πé│πâÆπéÜπâ╝/PNKP.png]

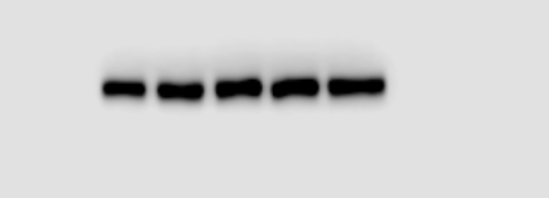

Supplement: Figure 6—figure supplement 1—source data 2. [file elife-99217-fig6-figsupp1-data2.zip › Figure 6-figure supplement Aπü«πé│πâÆπéÜπâ╝/KAP1.png]

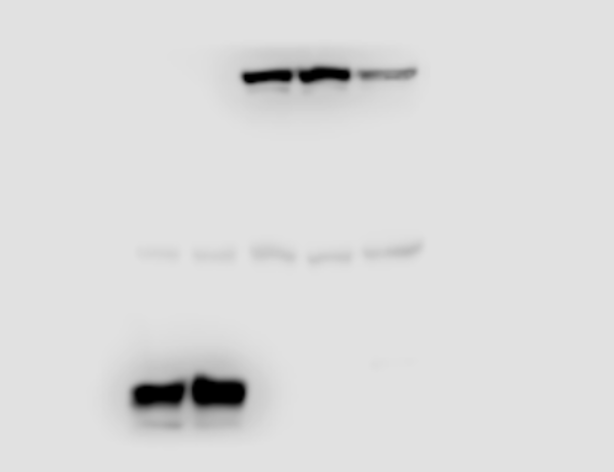

Supplement: Figure 6—figure supplement 1—source data 2. [file elife-99217-fig6-figsupp1-data2.zip › Figure 6-figure supplement Aπü«πé│πâÆπéÜπâ╝/GFP.png]

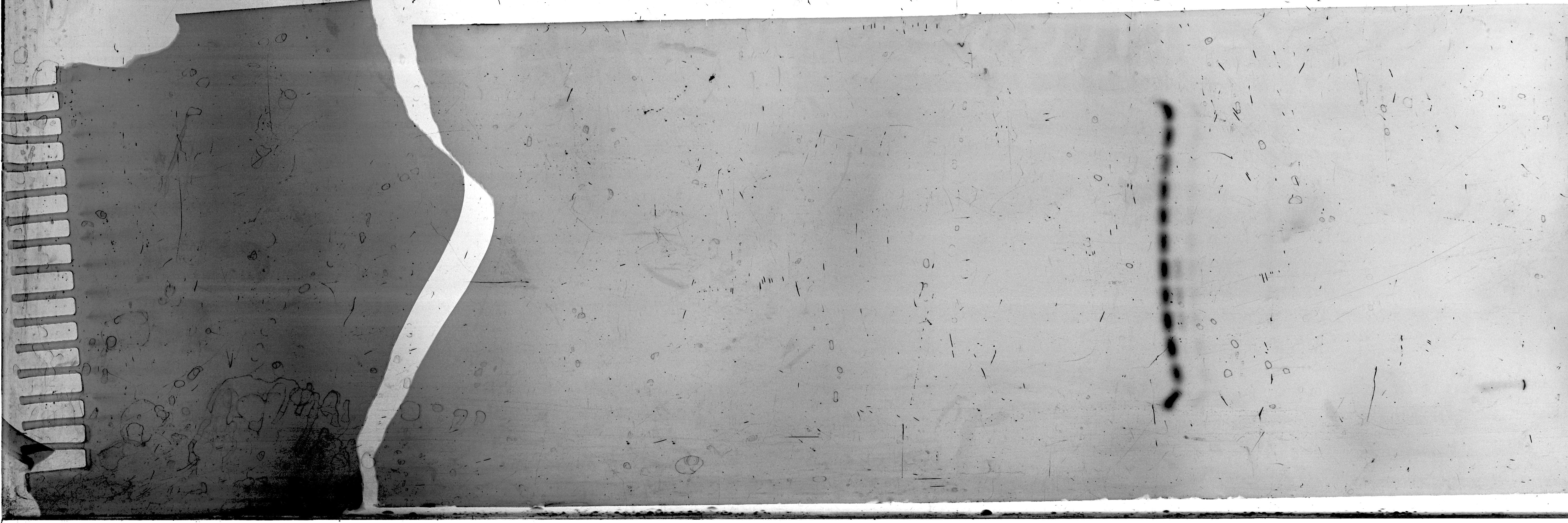

Supplement: Figure 6—figure supplement 1—source data 4. [file elife-99217-fig6-figsupp1-data4.zip › S6B_Source Data 2/Kinase activity.bmp]

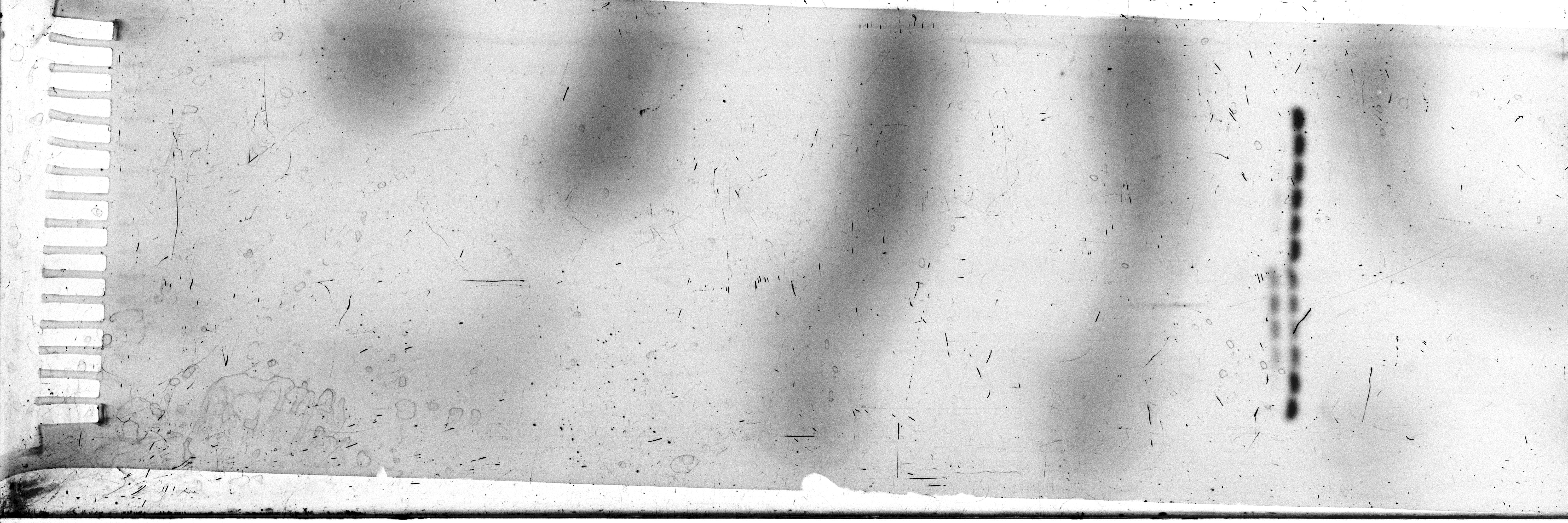

Supplement: Figure 6—figure supplement 1—source data 4. [file elife-99217-fig6-figsupp1-data4.zip › S6B_Source Data 2/Phosphatase activity.bmp]

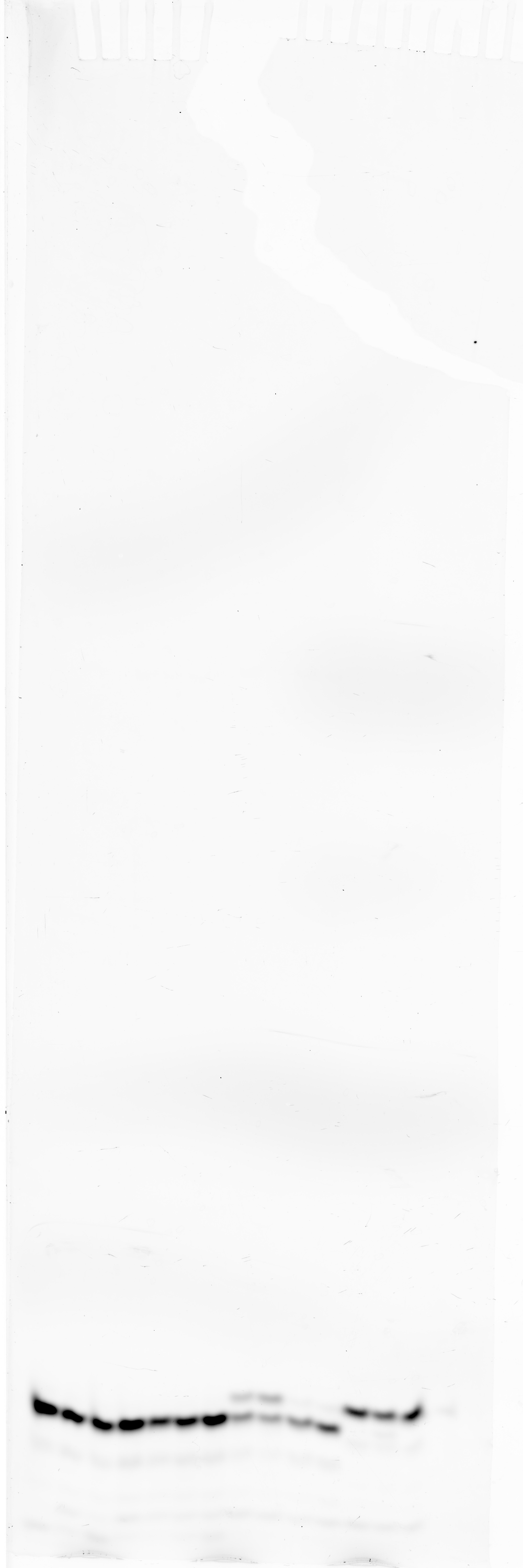

Supplement: Figure 6—figure supplement 1—source data 6. [file elife-99217-fig6-figsupp1-data6.zip › kinase acitivity.jpg]

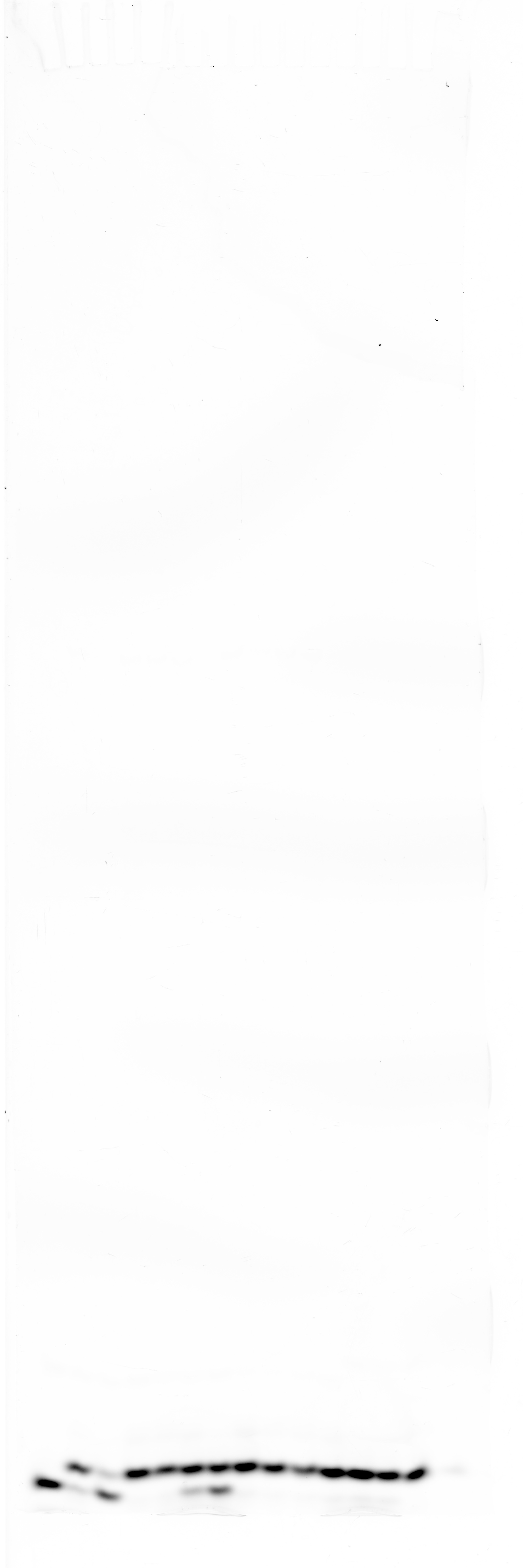

Supplement: Figure 6—figure supplement 1—source data 6. [file elife-99217-fig6-figsupp1-data6.zip › Phosphatase acticity.jpg]
