# Supplementary material for: CDK-mediated phosphorylation of PNKP is required for end-processing of single-strand DNA gaps on Okazaki fragments and genome stability
Source: eLife. 2025 Mar 27;14:e99217. doi: 10.7554/eLife.99217 (PMC11949490; doi:10.7554/eLife.99217)
Supplement: Figure 1—figure supplement 2—source data 1. — Regions surrounded with red dashed line represent cropped areas, respectively. Annotations represent employed antibodies, respectively. [file elife-99217-fig1-figsupp2-data1.pdf]

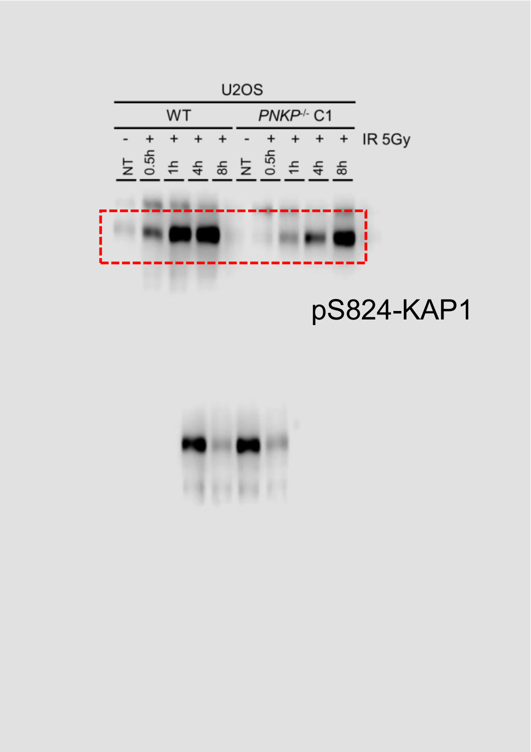
U2OS
WT
PNKP-/- C1
- + + + + - + + + +
IR 5Gy
NT 0.5h 1h 4h 8h NT 0.5h 1h 4h 8h
pS824-KAP1

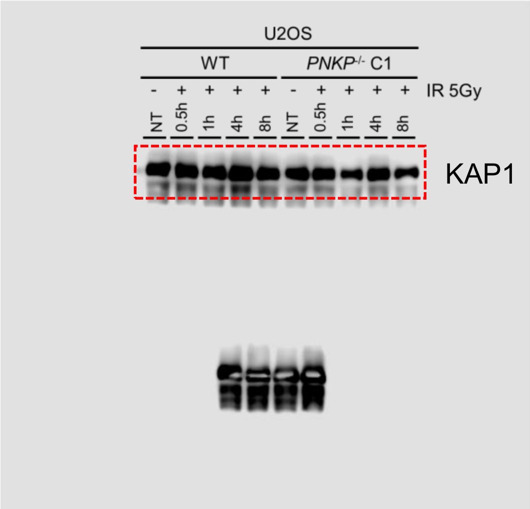
U2OS
WT
PNKP-/- C1
- + + + + - + + + +
IR 5Gy
NT 0.5h 1h 4h 8h NT 0.5h 1h 4h 8h
KAP1

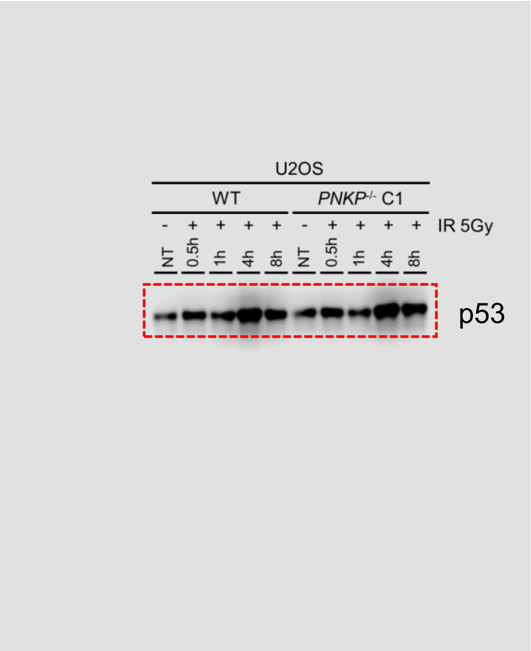
U2OS
WT
PNKP-/- C1
- + + + + - + + + +
IR 5Gy
NT 0.5h 1h 4h 8h NT 0.5h 1h 4h 8h
p53

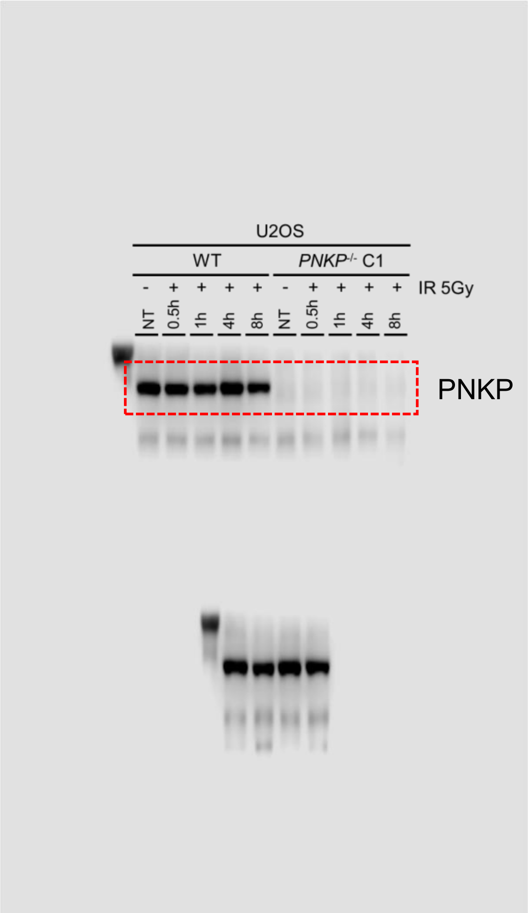
U2OS
WT
PNKP-/- C1
- + + + + - + + + +
IR 5Gy
NT 0.5h 1h 4h 8h NT 0.5h 1h 4h 8h
PNKP

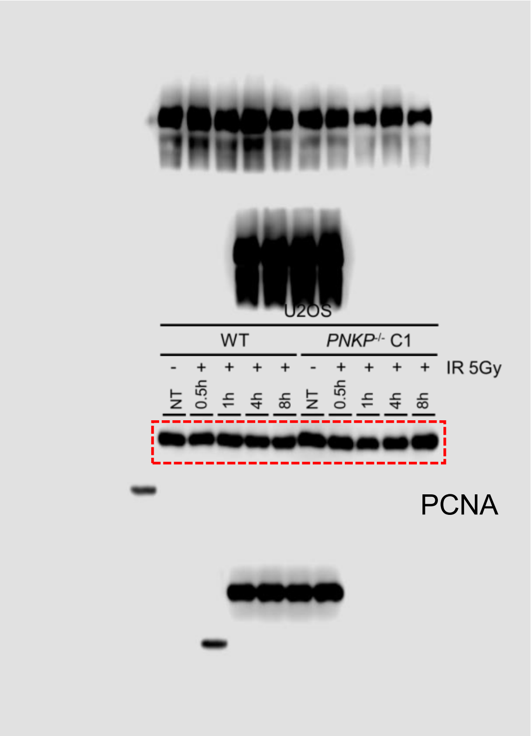

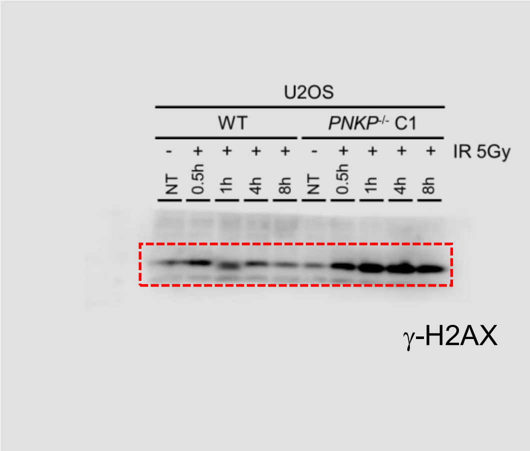

Figure 1-figure supplement 2A, Source Data1. Original membranes corresponding to Figure 1-figure supplement 2, panel A. Regions surrounded with red dashed line represent cropped areas, respectively. Annotations represent employed antibodies, respectively.
