# Supplementary material for: CDK-mediated phosphorylation of PNKP is required for end-processing of single-strand DNA gaps on Okazaki fragments and genome stability
Source: eLife. 2025 Mar 27;14:e99217. doi: 10.7554/eLife.99217 (PMC11949490; doi:10.7554/eLife.99217)
Supplement: Figure 4—source data 11. — Regions surrounded with red dashed line represent cropped areas, respectively. Annotations represent employed antibodies, respectively. [file elife-99217-fig4-data11.pdf]

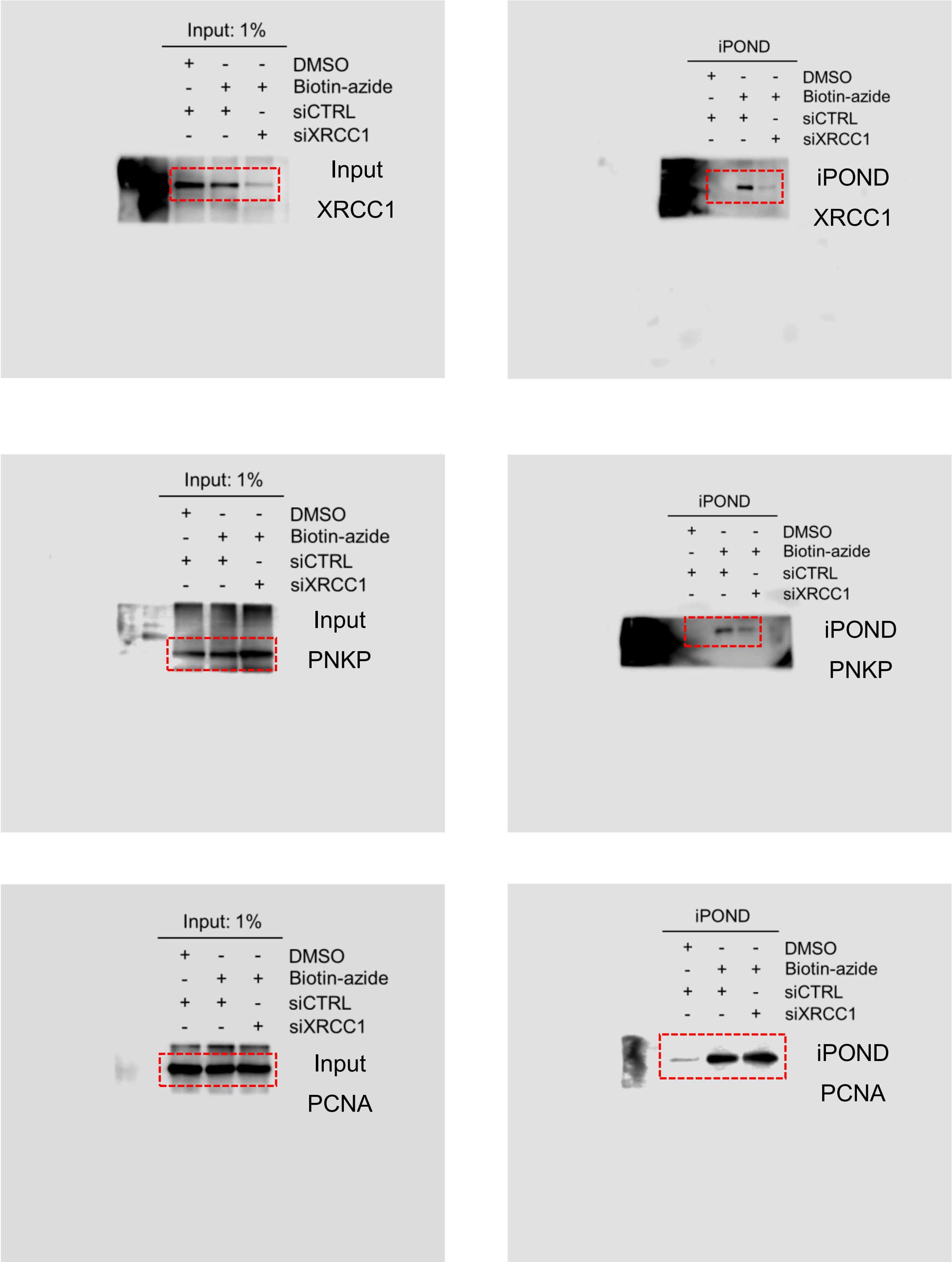

Figure 4F, Source Data1. Original membranes corresponding to Figure 4, panel F. Regions surrounded with red dashed line represent cropped areas, respectively. Annotations represent employed antibodies, respectively.
