# Supplementary material for: CDK-mediated phosphorylation of PNKP is required for end-processing of single-strand DNA gaps on Okazaki fragments and genome stability
Source: eLife. 2025 Mar 27;14:e99217. doi: 10.7554/eLife.99217 (PMC11949490; doi:10.7554/eLife.99217)
Supplement: Figure 5—figure supplement 1—source data 1. — Regions surrounded with red dashed line represent cropped areas, respectively. Annotations represent employed antibodies, respectively. [file elife-99217-fig5-figsupp1-data1.pdf]

Figure 5-figure supplement A

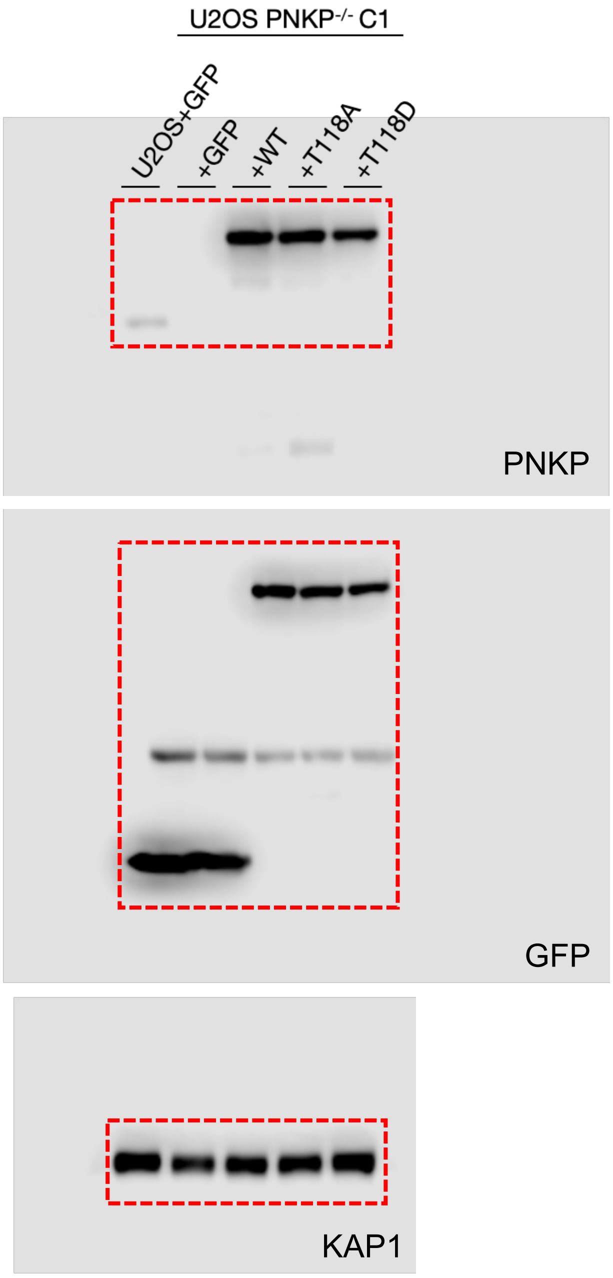

Figure 5-figure supplement A-Source Data 1.
Original membranes corresponding to Figure 5-figure supplement, panel A. Regions surrounded with red dashed line represent cropped areas, respectively. Annotations represent employed antibodies, respectively.
