# Supplementary material for: CDK-mediated phosphorylation of PNKP is required for end-processing of single-strand DNA gaps on Okazaki fragments and genome stability
Source: eLife. 2025 Mar 27;14:e99217. doi: 10.7554/eLife.99217 (PMC11949490; doi:10.7554/eLife.99217)
Supplement: Figure 6—figure supplement 1—source data 3. — Indicated number of cell extracts harvested from U2OS WT and PNKP−/− C1 cells were incubated with TAMRA or 6-FAM-labeled oligonucleotide duplex harboring a single-strand break (SSB) GAP structure. Arrows indicate the positions of the TAMRA-labeled phosphatase substrates (top: 1/2) and 6-FAM-labeled kinase substrates (bottom: 2/2). [file elife-99217-fig6-figsupp1-data3.pdf]

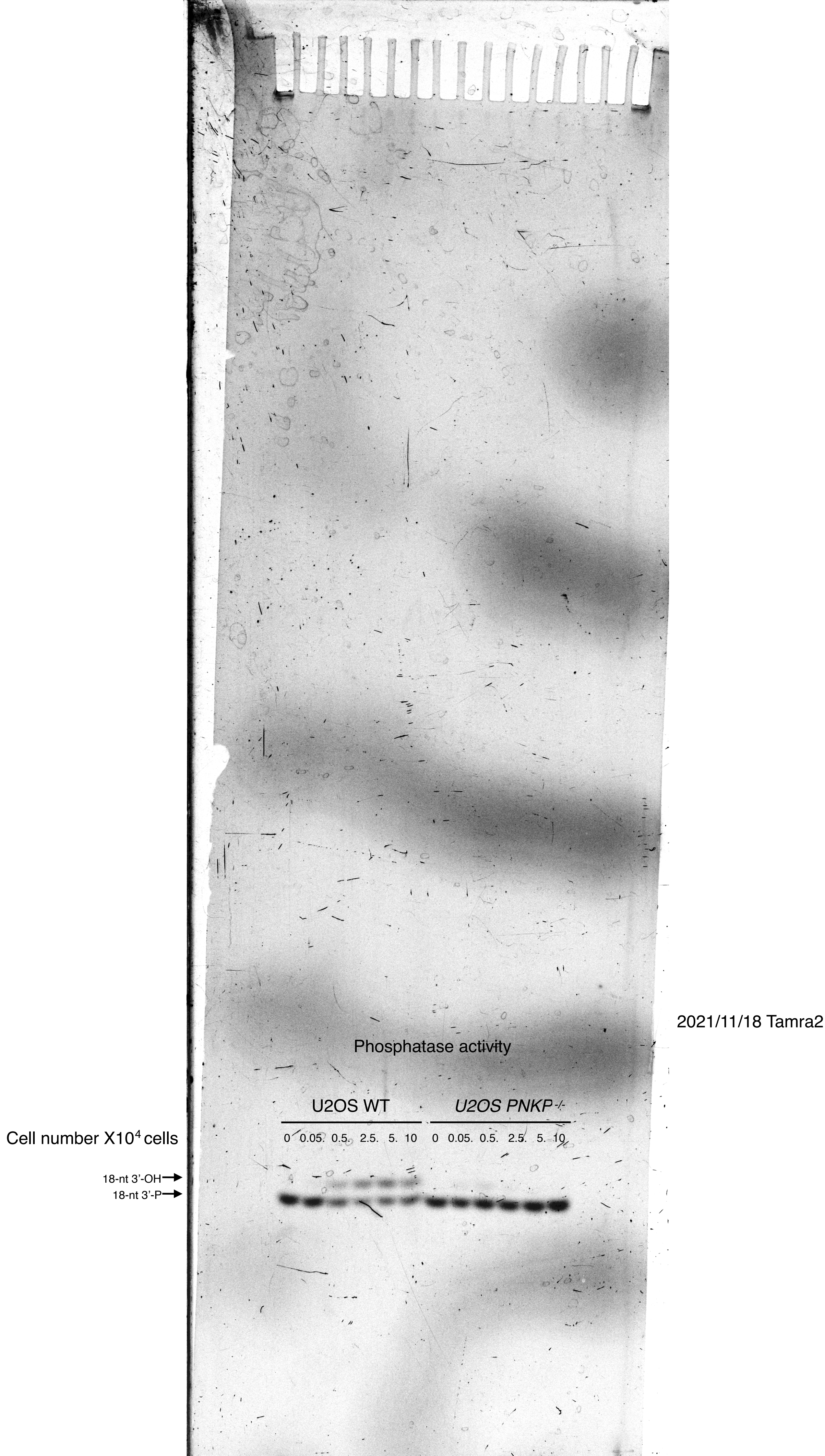
Phosphatase activity
U2OS WT
*U2OS PNKP-/-*
Cell number X10$^4$ cells
0 0.05. 0.5. 2.5. 5. 10
0 0.05. 0.5. 2.5. 5. 10
18-nt 3'-OH
18-nt 3'-P
2021/11/18 Tamra2

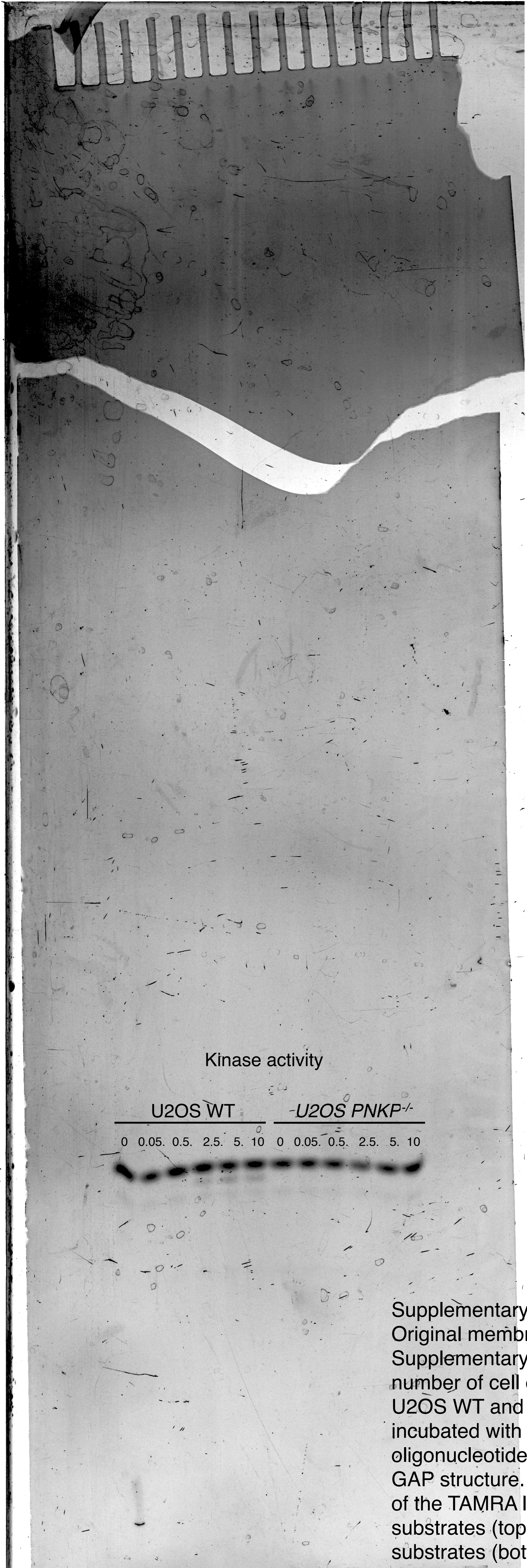


Supplementary Figure 6B, Source Data 1. Original membranes corresponding to Supplementary Figure 6, panel B. Indicated number of cell extracts harvested from U2OS WT and PNKP−/− C1 cells were incubated with TAMRA or 6-FAM labelled oligonucleotide duplex harboring a SSB GAP structure. Arrows indicate the positions of the TAMRA labelled phosphatase substrates (top) and 6-FAM labelled kinase substrates (bottom).
