# Supplementary material for: CDK-mediated phosphorylation of PNKP is required for end-processing of single-strand DNA gaps on Okazaki fragments and genome stability
Source: eLife. 2025 Mar 27;14:e99217. doi: 10.7554/eLife.99217 (PMC11949490; doi:10.7554/eLife.99217)
Supplement: Figure 6—figure supplement 1—source data 5. — Regions surrounded with red dashed line represent cropped areas. [file elife-99217-fig6-figsupp1-data5.pdf]

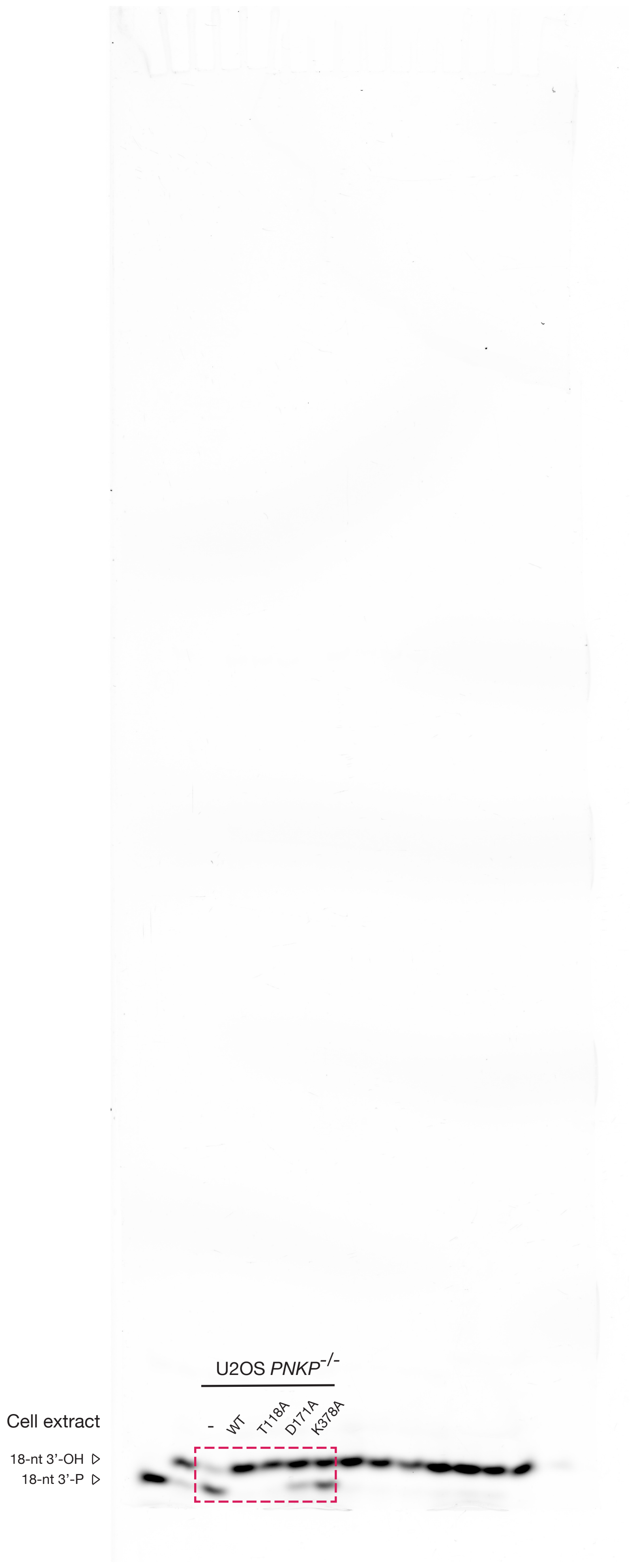
U2OS PNKP-/-
Cell extract
-
WT
T118A
D171A
K378A
18-nt 3'-OH
18-nt 3'-P

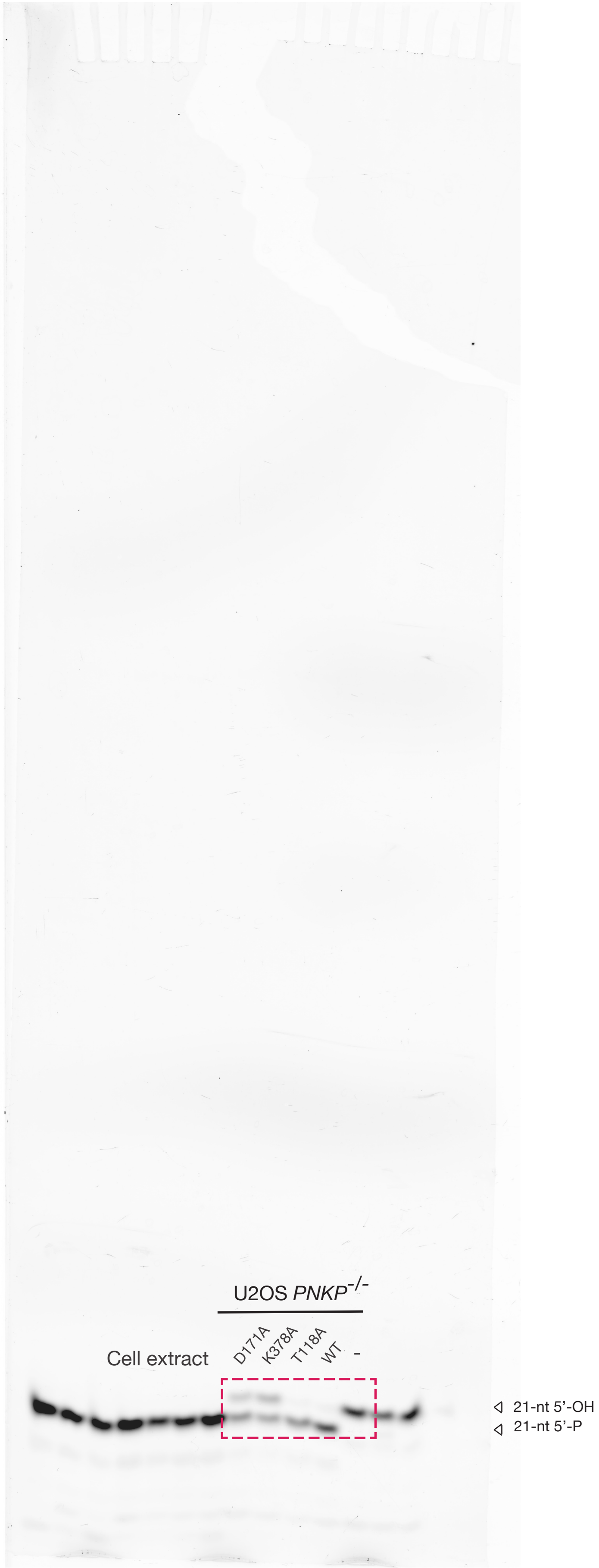

Figure 6-supplement C-Source Data1. Original gell corresponding to Figure 6-supplement, panel C. Regions surrounded with red dashed line represent cropped areas.
